# Targeting epigenetic regulators as a promising avenue to overcome cancer therapy resistance

**DOI:** 10.1038/s41392-025-02266-z

**Published:** 2025-07-18

**Authors:** Jiawei Song, Ping Yang, Canting Chen, Weiqun Ding, Olivier Tillement, Hao Bai, Shuyu Zhang

**Affiliations:** 1 Laboratory of Radiation Medicine, West China School of Basic Medical Sciences & Forensic Medicine, Sichuan University, Chengdu, China; 2 Department of Pathology, Stephenson Cancer Centre, College of Medicine, University of Oklahoma Health Sciences Center, Oklahoma City, OK USA; 3 Institut Lumière Matière, UMR 5306 CNRS-UCBL, Université Claude Bernard Lyon 1, Villeurbanne Cedex, France; 4 The Second Affiliated Hospital of Chengdu Medical College, China National Nuclear Corporation 416 Hospital, Chengdu, China; 5 Medical College of Tibet University, Lasa, China

**Keywords:** Epigenetics, Drug development, Molecular medicine

## Abstract

Cancer remains one of the leading health threats globally, with therapeutic resistance being a long-standing challenge across chemotherapy, radiotherapy, targeted therapy, and immunotherapy. In recent years, the association between epigenetic modification abnormalities and therapeutic resistance in tumors has garnered widespread attention, spurring interest in the development of approaches to target epigenetic factors. In this review, we explore the widespread dysregulation and crosstalk of various types of epigenetic modifications, including DNA methylation, histone modifications, and non-coding RNA changes, which interact through complex regulatory networks in tumors. Clinically, single-targeted therapy based on epigenetic modification usually has its limited effect against cancer. However, the combination of epigenetic drugs with other treatment modalities, such as chemotherapy, targeted therapy, or immunotherapy, shows potential for synergistically enhancing efficacy and reducing drug resistance. Therefore, we evaluate the possibility and potential mechanisms of targeting epigenetic modifications to overcome resistance in cancer therapy, and discuss the challenges and opportunities in moving epigenetic therapy into clinical practice. Moreover, the application of multi-omics technologies will aid in identifying core epigenetic factors from complex epigenetic networks, enabling precision treatment and overcoming therapeutic resistance in tumors. Furthermore, the development of spatial multi-omics technologies, by providing spatial coordinates of cellular and molecular heterogeneity, revolutionizes our understanding of the tumor microenvironment, offering new perspectives for precision therapy. In summary, the combined application of epigenetic therapies and the integration of multi-omics technologies herald a new direction for cancer treatment, holding the potential to achieve more effective personalized treatment strategies.

## Introduction

Cancer remains one of the leading causes of mortality worldwide, with the therapeutic resistance being a significant impediment to successful therapy.^1^ Despite advancements in chemotherapy, radiotherapy, immunotherapy, and targeted therapy, among all the possible reasons causing failure of anti-cancer treatments, development of therapeutic resistance accounts for up to 90% of cancer-associated deaths.^2^ The therapeutic resistance in cancer can be broadly classified into two categories: intrinsic (or de novo) and acquired resistance.^3^ Intrinsic resistance refers to the primary resistance exhibited by some cancers due to pre-existing genetic alterations or cellular states, which render them unresponsive to certain cytotoxic drugs and drug combinations from the outset. On the other hand, acquired resistance emerges during treatment as a result of an evolutionary process in which cancer cells adapt to survive therapeutic pressures. The acquired resistance can also arise through therapy-induced selection of pre-existing genetic alterations within the original malignancies, in which process epigenetic regulation plays a key role.^4^

The therapeutic resistance mechanism of cancer is multifaceted, involving genetic mutations, epigenetic alterations, cellular plasticity and so on. The mechanisms by which tumors develop resistance to various treatment modalities share both commonalities and differences. Almost all cancer hallmarks are closely related to tumor therapeutic resistance. Currently, a substantial amount of evidence indicates that there is an abnormal expression and activity of various epigenetic modifiers in tumors, leading to aberrant epigenetic modifications that are highly correlated with the malignant phenotype and therapeutic resistance of tumors.^5^ Epigenetic modifications, such as DNA methylation, histone modifications, and non-coding RNA regulation, are heritable changes in gene expression that do not involve alterations to the underlying DNA sequence. These modifications play a crucial role in the regulation of oncogenes and tumor suppressor genes expression and have been implicated in the development of cancer and resistance to therapy.^6^

In this review, we focus on the recent progress and provide an overview of the widespread epigenetic modification abnormalities in cancer. Then, the impact of epigenetic regulators on cancer therapeutic resistance will be discussed, exploring the mechanisms by which aberrant epigenetic regulations contribute to tumor resistance to treatment, and how targeting these regulators may offer a promising strategy to overcome the resistance. We will also highlight the roles of specific epigenetic modifiers (including their writers, erasers and readers), and their potential as therapeutic targets in cancer treatment. Furthermore, we underscore the importance of the interplay between different epigenetic modifications, which is a relatively unexplored area that could hold the key to understanding and overcoming therapy resistance. The future of epigenetic research in cancer therapy is promising but challenging. Among the various epigenetic modifications present in tumors, identifying which one is the core driver of malignant phenotypes is the most critical concern in clinical practice. Understanding the crosstalk between these epigenetic modifications will not only enhance our knowledge of cancer biology but also pave the way for the development of novel, targeted therapies that can effectively overcome resistance mechanisms. Furthermore, by leveraging multi-omics technologies, we can identify the core drivers among numerous epigenetic factors, which will enable a targeted approach to overcoming therapeutic resistance in cancer treatment, thus revolutionizing our ability to combat this complex disease. While the application of single-targeted epigenetic drugs alone in clinical oncology has not yet yielded the anticipated therapeutic outcomes, our review reveals the immense potential of combining epigenetic therapies with other treatment modalities to overcome therapeutic resistance. Rather than replicating several excellent reviews on the epigenetic modifications in relation to cancer, our aim here is to provide a novel perspective on the drivers of resistance in cancer therapy.

## Epigenetic regulators in cancer

**Fig. 1:**
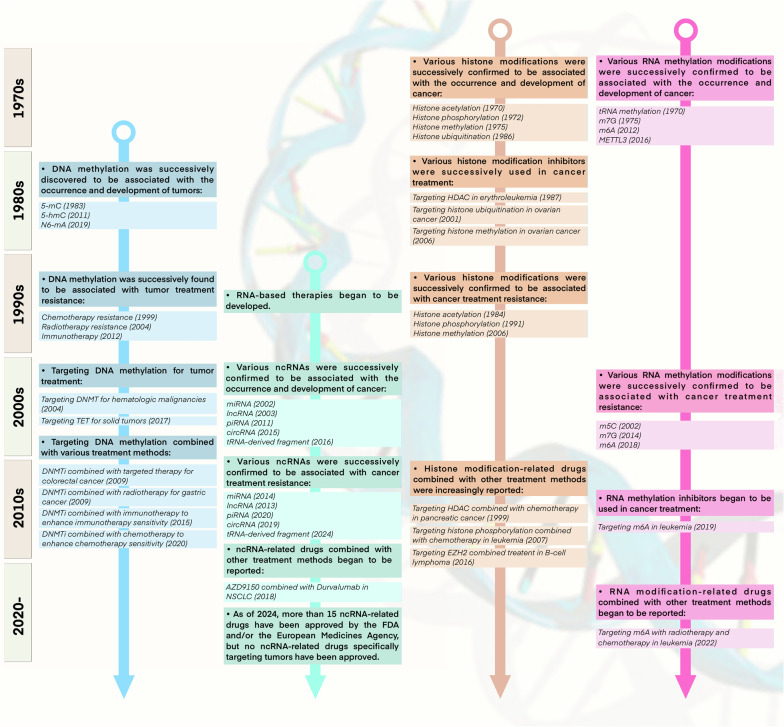
Evolution of combination of epigenetic modifications and cancer therapy. It delineates the historical milestones in the combination of epigenetic regulation with cancer therapy. The progression of the combination of epigenetics and cancer treatment is depicted from four aspects: the association of different epigenetic mechanisms with cancer, the relevance of epigenetics to cancer treatment resistance, and the application of epigenetic inhibitors in preclinical research and clinical trials. Distinct epigenetic mechanisms are noted by different colors

Epigenetics is fundamentally characterized by a diverse array of covalent modifications to histone proteins and nucleic acids, which collectively govern chromatin architecture and gene expression.^7^ These epigenetic modifications are reversible and subject to dynamic regulation, being initially established and later removed by specialized chromatin-modifying enzymes termed ‘writers’, ‘erasers’ and ‘readers'.^8^ Currently, the primary mechanisms of epigenetic regulation involve covalent modifications, including histone modification, DNA methylation, RNA modification, and non-coding RNAs^9^ (Fig. 1). These epigenetic patterns are intrinsically related to the occurrence, progression and treatment of tumors.^10^

### Histone modifications

**Fig. 2:**
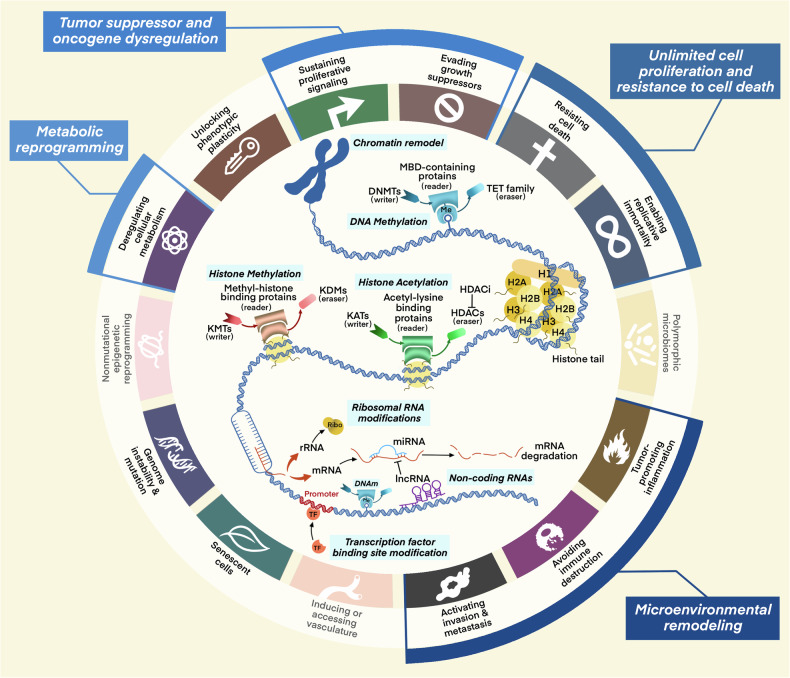
Hallmarks of cancer regulated by epigenetics. It illustrates the impact of epigenetic mechanisms on the hallmarks of cancer. The epigenetic elements are presented sequentially in the order of chromosome, DNA, and RNA, with a concise description of their functions and regulatory roles. Furthermore, the figure emphasizes the principal hallmarks influenced by epigenetic modifications, specifically highlighting the dysregulation of tumor suppressors and oncogenes, the promotion of unlimited cell proliferation and resistance to apoptosis, microenvironmental remodeling, and metabolic reprogramming, which are mentioned in the review. DNMT DNA methyltransferase, TET ten-eleven translocation, HDAC histone deacetylase, KDM lysine demethylase, KMT lysine methyltransferase, KAT lysine acetyltransferase, MBD methyl-CpG binding domain

Histone modifications are key regulatory mechanisms in epigenetics that modulate chromatin structure and gene expression by adding or removing specific chemical groups on histones.^11–13^ These modifications mainly include acetylation, methylation, phosphorylation, and ubiquitination. Since the first isolation and discovery of histone acetylation in 1964,^14^ various forms of histone modifications, including such as methylation, phosphorylation, and ubiquitination have been gradually revealed (Fig. 1). In recent years, many novel histone modifications have been discovered, such as citrullination, crotonylation, succinylation, propionylation, butyrylation, 2-hydroxyisobutyrylation, and 2-hydroxybutyrylation.^15–22^ These histone modifications are essential for preserving the integrity of chromatin architecture, regulating DNA transcription, replication, repair, and recombination, have a close connection with the onset and development of various cancers^23–26^ (Fig. 2). In particular, representative histone modifications such as acetylation, methylation, phosphorylation, and ubiquitination have been extensively studied in the field of cancer therapy.^27^ These modifications are not only related to the development of cancer but also to therapeutic resistance in cancer treatment.^28–30^ Within this treatise, we will discuss the intricate role of histone modifications in oncogenesis and their pivotal impact on the resistance to a spectrum of therapeutic modalities.

### DNA methylation

DNA methylation represents an epigenetic modification, entailing the attachment of a methyl group to specific bases within the DNA molecule.^31^ This modification can trigger alterations in DNA conformation, DNA stability, chromatin structure, and the interplay between DNA and proteins, thereby exerting a regulatory influence on gene transcription. Specifically, DNA methylation serves as a physical barrier that hinders transcription proteins from binding to genes.^32^ Methyl-CpG-binding domain (MBD) proteins act as attractors, recruiting a variety of cofactors including histone deacetylases (HDACs) and chromatin reorganization proteins to the methylated DNA loci. Subsequently, compacted heterochromatin is formed, which functions to repress transcriptional activity.^33^. DNA methylation predominantly occurs at the fifth carbon of cytosine, forming 5-methylcytosine (5mC). In mammals, this modification is predominantly witnessed in CpG islands.^34^ Moreover, 5mC can be oxidized to yield derivatives such as 5-hydroxymethylcytosine (5-hmC), 5-formylcytosine (5-fC), and 5-carboxylcytosine (5-caC). These 5mC derivatives have been found to be associated with active gene expression and contribute to cell development and the pathogenesis of diseases.^35^ Recent investigations have demonstrated that 5mC derivatives are closely related to the tumors development^36^ (Fig. 2). Apart from the aforementioned forms, there exist other manifestations of DNA methylation, including N6-methyladenine (N6-mA) and 4-methylcytosine (4-mC).^37,38^ Given its influence on regulation of DNA transcription, DNA methylation is integral to in a diverse array of biological processes including tumorigenesis, aging, and disease occurrence.^39–41^ Here, we will delve into the intricate and multifaceted role that DNA methylation plays in cancer progression and its implications regarding resistance to various therapeutic interventions.

### RNA modifications

To date, over 100 distinct chemical modifications have been discovered on RNA within eukaryotic organisms. The study of RNA modifications can be traced back to the 1950s. Pseudouridine (Ψ) was the first type of RNA modification identified in 1950,^42^ and in 1965, the sequencing of yeast alanine tRNA confirmed 10 types of modifications. In 1975, RNA methylation at m^6^A was first observed^43–45^ (Fig. 1). Currently, RNA modifications mainly include N1-methyladenosine (m^1^A), 5-methylcytosine (m^5^C), N6-methyladenosine (m^6^A), 7-methylguanosine (m^7^G), pseudouridine (Ψ), and adenosine-to-inosine (A-to-I) editing.^46^ These modifications impact RNA stability, translation efficiency, and protein interactions, thereby influencing cell fate. Furthermore, it has been demonstrated that the abnormalities in RNA modifications are closely associated with the occurrence and development of various cancers (Fig. 2). For example, the m6A modification stands as the most prevalent form of methylation occurring in mRNA, and its abnormal changes are intimately connected with tumor proliferation, growth, invasion, and metastasis.^47^ m^6^A modification affects gene expression, transcription regulation, and immune evasion through influencing the structure and function of mRNA, thus becoming a new target for tumor treatment.^48–50^ In addition, m^7^G and m^5^C can also affect tumor occurrence and development through various mechanisms.^51,52^ RNA modifications exert significant regulatory influence on the occurrence and development of tumors, including affecting gene expression, cell cycle regulation, cell migration, and invasion capabilities. These revelations offer novel therapeutic targets and strategic avenues for oncological intervention.

### Non-coding RNAs

It is widely acknowledged that ncRNAs are implicated in oncogenesis due to somatic genomic instability and the accumulation of mutations.^53,54^ Non-coding regions constitute over 97% of the entire genome. These regions encompass non-coding RNA genes, which possess the potential for transcription, as well as regulatory elements that are not transcribed. Besides, these regions also incorporate repetitive sequences and other segments that remain unelucidated.^55^ ncRNAs refer to RNA molecules which are not involved in protein - encoding and constitute an essential part of the transcriptome.^56^ They execute pivotal regulatory functions within a multitude of cellular processes via post-transcriptional mechanisms, exerting a profound influence on gene transcription and translation, cell proliferation, differentiation, senescence, apoptosis, and both genetic and epigenetic pathways^57–60^ (Fig. 2). The diversity of ncRNAs is extensive, including ribosomal RNA (rRNA), transfer RNA (tRNA), microRNA (miRNA), long non-coding RNA (lncRNA), and small interfering RNA (siRNA).^61^ With the continual advancements and implementation of high-throughput sequencing technologies, additional categories of non-coding RNAs, such as PIWI-interacting RNA (piRNA),^62,63^ tRNA-derived fragments (tRFs or tsRNA),^64^ and circular RNA (circRNA)^65^ have also been identified in recent years. Non-coding RNAs possess the inherent capacity to modulate cellular activities through interactions with DNA/chromosomes, other RNAs, and proteins, thereby forging a highly intricate ncRNA network.^66^ Virtually all epigenetic processes, including histone modifications, DNA modifications, and chromatin architecture, fall under the regulatory purview of ncRNAs. Consequently, ncRNAs have a vital impact on the interplay of epigenetic modifications. Our empirical study focuses on ncRNAs with regulatory functions in cancer, such as miRNAs, lncRNAs, piRNAs, and circRNAs, rather than the housekeeping ncRNAs customarily engaged in basic cellular functions.

### Others

#### Chromatin remodeling

Chromatin remodeling stands as one of the significant mechanisms for regulating gene expression, exerting its influence on it by altering the configuration and composition of chromatin. Chromatin remodeling complexes (including SWI/SNF, BAF/PBAF, and others) harness the energy derived from ATP hydrolysis to translocate nucleosomes or modify the interaction between histones and DNA, thereby governing the accessibility and expression of genes.^67^ In the occurrence and development of tumors, chromatin remodeling plays a pivotal role. Studies have shown that mutations or dysregulation of chromatin remodeling factors can lead to abnormal gene expression, thereby promoting tumorigenesis and the subsequent advancement of malignancies.^68^ For instance, the SWI/SNF complex participates in tumor therapeutic resistance and progression, and mutations within its subunits can impact the responsiveness of tumor cells to treatment.^69^ In addition, chromatin remodeling is also related to tumor microenvironment composition and immune evasion, affecting the survival and metastasis of cancer cells.^70,71^ Chromatin remodeling is also involved in the regulation of DNA damage repair pathways in tumor cells, such as the chromatin modifications mediated by poly ADP-ribose polymerase 1 (PARP1) during DNA repair.^72^ In summary, chromatin remodeling plays a pivotal role in tumor initiation, progression, and therapeutic response. A deeper understanding of its mechanisms not only aids in elucidating the molecular foundations of tumorigenesis but also provides a theoretical basis and potential targets for the development of innovative anti-cancer therapies.

#### Ribosomal RNA modifications

Ribosomal RNA modification constitutes a significant post-transcriptional modification mechanism within the biological landscape, being ubiquitously present across a diverse array of organisms. These modifications enhance the efficiency of protein translation by ribosomes through altering the local spatial structure of rRNA molecules.^73^ Three categories of chemical modifications are manifested in rRNA: ribose methylation (Nm), the isomerization of uridine to pseudouridine (Ψ), along with base modifications such as methylation (mN), acetylation (acN), and aminocarboxypropylation (acpN).^74,75^ These modifications have endured throughout the evolutionary process and are predominantly mediated by specific enzymes including Fibrillarin and small nucleolar RNAs (snoRNAs). rRNA modifications are of paramount importance for the functionality of ribosomes. They also participate in regulating the assembly of ribosomal subunits and translation functions.^76^ rRNA modifications not only affect the structure and function of ribosomes but may also be related to the occurrence of diseases. For instance, the methylation modification of rRNA engages in tumor growth by regulating ribosomal translation.^77^ In summary, rRNA modifications are integral to regulating translation efficiency, optimizing protein synthesis, and affecting the growth of organisms as well as the onset of diseases. Research into these modifications is conducive to deepening our comprehension of ribosomal function and its regulatory mechanisms.

#### Transcription factor binding site modifications

Transcription factor binding site modification refers to the chemical alterations that transpire when transcription factors (TFs) bind to specific sequences on DNA. These modifications may include DNA methylation, acetylation, phosphorylation, and various post-translational modifications of histones.^78^ Such chemical modifications possess the capacity to alter the interaction between DNA and histones, thereby exerting an impact on the binding affinity, stability, and efficiency of transcription factors in the modulation of gene expression.^79^ Modifications of transcription factor binding sites are of great significance in regulating gene expression. They are capable of transforming the chromatin structure, either facilitating or impeding the access of transcription factors to their target genes, thus enabling a precise regulation of gene activity. For example, histone acetylation is usually associated with gene activation, while methylation may be related to gene silencing.^80,81^ In the genesis and progression of tumors, alterations to transcription factor binding sites also exert a significant influence. Abnormal modification patterns may lead to gene expression dysregulation, thereby promoting tumor growth and spread. For example, abnormal methylation of certain transcription factors could cause the inactivation of tumor suppressor genes, while abnormal acetylation in some enhancer regions may promote the expression of oncogenes.^80^ In addition, modifications of transcription factor binding sites may also affect the process of chromatin remodeling, which is a dynamic change in the structure of chromatin within the cell nucleus, and is crucial for gene expression regulation, DNA replication, DNA repair, and cell division, among other nuclear activities.^82^ Overall, transcription factor binding site modification constitutes one of the crucial mechanisms for gene expression regulation, and its abnormalities may be linked to the emergence and progression of diverse diseases, including tumors. Consequently, delving into the patterns and regulatory mechanisms of these modifications is of paramount importance for comprehending the gene regulatory network and devising novel treatment strategies.

## Abnormal epigenetic landscapes drive cancer therapeutic resistance

**Fig. 3:**
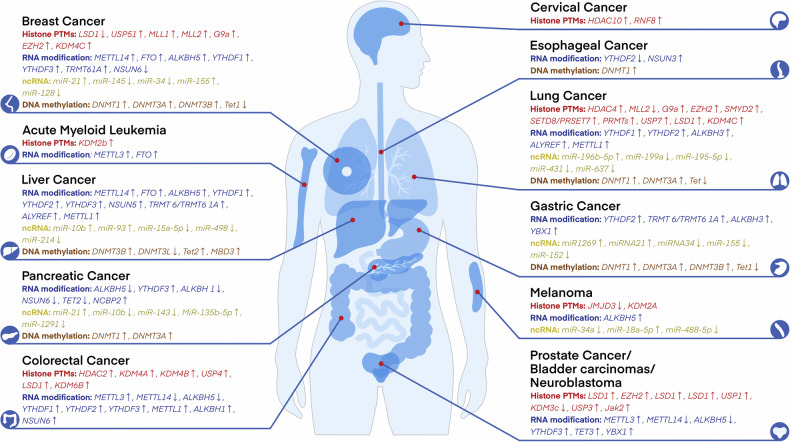
Abnormal epigenetic landscapes in various cancers. Aberrant expression of epigenetic regulators is presented across various cancer types. Distinct colors are used to denote different epigenetic pathways, while arrows indicate the direction of expression changes, either up-regulation or down-regulation

Alterations in the patterns of post-translational modifications (PTMs) have been extensively linked to cancer, whether considering the global level across the entire genome or the genetic information and functional status of cells.^27,83^ Over the past several decades, a multitude of histone modifications have been successively verified to bear a connection with the initiation and progression of cancer. Furthermore, during the last two decades, there has been an extensive and vigorous endeavor in the pharmacological targeting of these pathways for the purpose of intervening in cancer, which resulted in the occurrence of a number of novel cancer therapies^84^ (Fig. 1). Subsequently, the aberrant expression of histone modification regulators in diverse types of cancers will be expounded upon below (Fig. 3).

### Histone post-translational modifications

#### Histone acetylation and deacetylation

Histone acetylation, a sophisticated epigenetic modification, plays a vital physiological role in the regulation of gene transcription by modulating the chromatin structure. Histone acetylation entails the addition of the acetyl groups (-COCH3) to lysine residues on histones. Acetylation can neutralize the positive charge of lysine residues, thereby diminishing the binding affinity between DNA and histones, leading to a relaxation of chromatin structure, thereby allowing transcriptional regulatory factors to bind and promoting gene expression.^85^ Histone acetylation is governed by two competing families of enzymes: histone lysine acetyltransferases (KATs), which add acetyl groups, and HDACs, which remove acetyl groups.^86^ The KAT family is categorized into two main types: type A and type B. Type A KATs are primarily located in the nucleus and can be further subdivided into three major families: the GNAT superfamily, the MYST family, and the CBP/p300 family.^87^ In contrast, type B KATs are mainly present in the cytoplasm and modify free histones.^88^ The HDAC family encompasses 18 distinct enzymes, categorized into four primary classes based on their sequence homology to yeast proteins. Class I, resembling the Rpd3-like enzymes, includes HDAC1, HDAC2, HDAC3, and HDAC8. Class II, akin to the Hda1-like enzymes, is bifurcated into two subclasses: Class IIa, which consists of HDAC4, HDAC5, HDAC6, HDAC7, and HDAC9, and Class IIb, comprising HDAC6 and HDAC10. Class III, the Sir2-like enzymes, encompasses SIRT1 through SIRT7. Lastly, Class IV is singular, containing only HDAC11.^89^

Histone acetylation is a crucial factor in the evolution and advancement of malignancies. The imbalance between HAT and HDAC activities leads to abnormal changes in histone acetylation levels, which may disrupt gene transcription regulation and participate during the appearance and growth of tumors.^90^ Specifically, in human and mouse tumor samples, the levels of H4K16 acetylation (H4K16ac) and H4K20 trimethylation (H4K20me3) have been significantly reduced, and these changes have been confirmed as biomarkers for tumor progression.^91,92^ Alongside the variations in histone acetylation levels, the expression of enzymes related to histone acetylation is also altered in cancer. These alterations are not only significant markers of cancer development but also serve as potential biomarkers and therapeutic targets in clinical practice. Specifically, general control of general nucleotide synthesis 5 (GCN5), which is one type of KATs, activation of this was detected in human glioma, colon cancer, breast cancer and lung carcinoma.^93–95^ Conversely, in solid tumors including ovarian, gastric, and esophageal cancer, the p300-CBP-associated factor (pCAF) is commonly diminished.^83^ Additionally, the aberrant expressions of KAT4, KAT5, MYST1, MYST3, MYST4, KAT2A, KAT2B, and p300 has been noted in colorectal cancer (CRC) as well. In contrast, overexpression of KAT2A, KAT2B, KAT4, and MOF is a characteristic feature of malignant kidney tumors.^96,97^ In contrast, KAT2B is downregulated in gastric cancer cells and appears to be positively correlated with the CDKN1A tumor suppressor mRNA levels.^98^ In addition, upregulation of lysine acetyltransferase 7 (KAT7) was observed in multiple breast cancer cell lines, which was found to enhance the PI3K/AKT signaling pathway and confer radioresistance by activating the transcription of PIK3CA^99^ (Fig. 3).

In addition to histone KATs, HDACs are also frequently dysregulated in tumors. For example, HDAC1 and HDAC2 expression levels are moderately elevated in papillary thyroid carcinoma tissues compared with normal tissues.^100^ Besides, HDAC1 is reported to be expressed in a significant proportion of cancers, such as gastric carcinoma, prostate cancer, lung cancer, breast cancer, and colon cancer^101^ (Table 1). However, HDAC2 is overexpressed in hepatocellular carcinoma (HCC), CRC and glioma.^102,103^ Furthermore, colon and breast cancers are notably characterized by elevated HDAC3 expression levels. In contrast, neuroblastoma cells are distinguished by a significant abundance of HDAC8.^6^ The activity of class III HDACs, specifically SIRT1, SIRT4, and SIRT7, is found to be heightened in myeloid leukemia, prostate and ovarian carcinoma, as well as non-melanoma skin cancers. Conversely, a reduction in SIRT2 expression has been documented in gliomas, gastric carcinomas, and melanomas^104^ (Fig. 3). Overall, aberrant histone acetylation landscape is intricately implicated in the pathogenesis of cancer and provide a novel way for cancer treatment.

**Table 1:** Epigenetics landscape in cancers

Cancer	Regulator alteration	Mechanism	Ref.
Colorectal cancer	HDAC2↑	Upregulate the levels of c-fos and c-Jun.	^806^
	USP4↑	Reduce the expression of p53 and promote DDR	^807^
	KDM6B↑	Regulate Wnt signaling, interacts with p53	^808^
Melanoma	JMJD3↓	Upregulate the EMT	^809^
Lung cancer	G9a↑	Reduce cell proliferation	^810^
	EZH2↑	Promote progression and metastasis	^811^
	SMYD2↑	Repress p53 function	^812^
	SETD8/PRSET7↑	Deregulate PCNA expression	^813^
	PRMTs↑	Promote EMT	^814^
	USP7↑	Stabilize CHK1 and β-catenin through its DUB activity and promotes DDR	^815–817^
	LSD1↑	Regulate chromatin remodeling	^818^
	KDM4C↑	Promote TGF-β2 transcription	^120^
Cervical cancer	HDAC10↑	Regulate DNA mismatch repair	^819^
	RNF8↑	Promote the accumulation of p53-binding protein 1 (53BP1) and breast cancer type 1 susceptibility protein (BRCA1) at DSBs	^820^
Breast cancer	LSD1 ↓	Regulate several TGF-β1 signaling pathway that are critically involved in cell proliferation, survival, and epithelial-to-mesenchymal transition	^821^
	USP51↑	Interact with and stabilize DiGeorge syndrome critical region 8 and promote the recruitment of DGCR8 and its partner RNF168 at DSBs	^806^
	MLL1↑	Promote proliferation	^822^
	MLL2↑	Upregulate c-Myc	^823^
	G9a↑	Repress FBP1 to promote cancer stem cell	^824^
	EZH2↑	Repress CDKN1B to promote proliferation	^825^
Prostate cancer, bladder carcinomas, neuroblastoma	LSD1↑	Activate AR-mediated growth signals	^826^
	EZH2↑	Inhibit cell proliferation	^111^
	LSD1↑	Regulate chromatin remodeling	^818^
	LSD1↑	Promote AR-dependent transcription	^827^
	USP1↑	Deubiquitinate and stabilize inhibitor of DNA binding 1 (ID1) and CHK1 to promote DDR	^828^
	USP3↑	Activate the ATR-CHK1 signaling pathways and lead to the enhancement of DDR	^829^
	Jak2↑	DNA repair structure, transcription and chromatin compaction	^124^
Acute myeloid leukemia	KDM2b↑	Impair Hoxa9/Meis1-induced leukemic transformation	^830^
Colorectal cancer	METTL3↑	Enhance tumor growth and metastasis	^203^
	METTL14↓	Regulate YAP 1 to promote cancer growth by down-regulating SP 1	^831^
	ALKBH5↓	Inhibited invasion metastasis	^832^
	YTHDF1↑	Wnt/β-catenin pathway.	^242^
	YTHDF2↑	Cell proliferation and invasion	^241^
Melanoma	ALKBH5↓	Loss of ALKBH5 inducing G1 to S phase transition arrest	^229^
Lung cancer	YTHDF1↑	Promote CDK2 and CDK4 expression	^243^
	YTHDF2↑	Promote the cancer growth	^245^
	ALKBH3↑	Result in the senescence induction and cell cycle arrest	^266^
	ALYREF↑	Inhibit the expression of YAP to promote invasion	^296^
	METTL1↑	Promote cell proliferation, migration, and invasion	^299^
Breast cancer	METTL14↑	Improve expression of IGF2BP2, leading to the malignant progression	^833^
	FTO↑	Inhibit the stabilization of the Wnt-ligand WNT5A	^225,833^
	ALKBH5↑	Increase of the expression of NANOG	^227^
	YTHDF1↑	Promote cell proliferation and EMT	^241^
	YTHDF3↑	Promote translation of oncogenes	^248^
Prostate cancer bladder carcinomas neuroblastoma	METTL3↑	Promote cancer progression via AFF4/NF-κB/MYC signaling network	^200^
	METTL14↓	Inhibit Notch1 expression and stability and promote the development	^282^
	ALKBH5↓	Decrease WIF-1 and inhibit tumorigenesis	^233^
	YTHDF3↑	Promote the translation of ITGA6	^251^
Acute myeloid leukemia	METTL3↑	Promote the translation of c-MYC, BCL2 and PTEN mRNAs	^199^
	FTO↑	Degradation of ankyrin repeat, ASB2 and RARA	^248^
	FTO↑	Inhibit apoptosis	^806^
	ALKBH5↑	Correlates with poor prognosis	^227^
	YTHDF1↑	Promote cell proliferation and EMT	^241^
	YTHDF3↑	Promote tumor growth	^251^
	TRMT 6/TRMT6 1A↑	Increase PPARδ translation	^260^
	ALYREF↑	Upregulate eIF4A3 expression resulting in abnormal cell cycle and mitosis	^295^
Pancreatic cancer	ALKBH5↓	Linked to poor prognosis	^234^
	YTHDF3↑	Stabilize MYC mRNA to promote cancer progression	^252^
	NCBP2↑	Activate the c-JUN/MEK/ERK pathway	^322^
Esophageal cancer	YTHDF2↓	Promote apoptosis	^834^
	NSUN3↑	Enhance metastasis by stimulating the translation of mitochondrial mRNA	^277^
Gastric cancer	YTHDF2↑	Promote apoptosis	^247^
	TRMT 6/TRMT6 1A↑	Promote the proliferation of cancer cells	^835^
	ALKBH3↑	Promote the proliferation of cancer cell	^258^
Pancreatic cancer	DNMT1↑	Maintain the methylation status after DNA replication,	^836^
Gastric cancers	DNMT1↑	Promote the proliferation and distant metastasis of gastric cancer cells	^837^
	DNMT3A↑	Promote the distant metastasis of gastric cancer cells	^838^
	DNMT3B↑	By promoting DNA methylation and inhibiting the expression of MYH11, the inhibitory effect of MYH11 on TNFRSF14 transcription is weakened and gastric cancer progression is promoted	^839^
Lung cancer	DNMT1↑	Act as a TSG to stabilize DNA methylation patterns	^178^
	DNMT3A↑	Promote the proliferation and distant metastasis of lung cancer cells	^838^
	Tet↓	Affects the Wnt/β - catenin signaling pathway and the expression levels of key genes CTNNB1 and MMP7, inhibits the migration and invasion of lung cancer cells	^840^
Breast cancer	DNMT1↑	Repression of estrogen receptor (ER) expression	^837^
	DNMT3A↑	Mediated hypermethylation of DPT and promoter leads to down-regulation of DPT expression in breast cancer	^841^
	DNMT3B↑	Regulating multiple cancer promoting signaling pathways	^842^
	Tet1↓	Promote the proliferation and distant metastasis of breast cancer cells	^843^
Liver cancer	DNMT3B↑	Promoting the proliferation and invasion of liver cancer cells	^844^
	DNMT3L↓	Inhibit cell proliferation and metastasis	^845^
	Tet2↑	Affects the expression of E-cadherin in a non-demethylation dependent manner	^846^
	MBD3↑	Inhibiting the transcription and protein expression of tumor suppressor gene TFPI2 promotes the growth and metastasis of liver cancer cells	^193^
	DNMT1↑	Increased DNA methylation and reducing RORA expression to affect SLC2A3 transcription and glycolysis	^847^
Gastric cancer	miR1269 ↑	Regulate of the AKT signaling pathway and the Bax/Bcl-2 signaling pathway	^848^
	miRNA21 ↑	Inhibit cell apoptosis, promote cell proliferation and migration	^355^
	miRNA34 ↓	Promote apoptosis, senescence, and cell cycle arrest and repress gastric cancer cell proliferation, migration and metastasis	^849^
	miR-155 ↓	Inhibit cell migration, invasion, and adhesion	^361^
	miR-152 ↓	Suppress gastric cancer cell proliferation and motility	^850^
Pancreatic cancer	miR-21 ↑	Stimulate invasion, intravasation and metastasis	^851^
	miR-10b ↓	Inhibits migration and invasion via regulating E2F7	^852^
	miR-143 ↓	Promote cell apoptosis and inhibit the migration and invasion of pancreatic cancer cells	^853^
	miR-135b-5p↑	Promote malignant progression by regulating GPRC5A	^854^
Lung cancer	miR-196b-5p ↑	Promote lung cancer cell migration, proliferation, and cell cycle	^855^
	miR-199a ↓	Inhibit the proliferation, infiltration, and migration of lung cancer cells, inhibit tumor angiogenesis, increase the apoptosis	^856^
	miR-195-5p ↓	Inhibit cell proliferation, migration, and invasion via FOXK1	^857^
	miR-637 ↓	Inhibit cell proliferation, migration, and invasion	^858^
Breast cancer	miR-21 ↑	Promote invasion, angiogenesis and metastasis	^859^
	miR-145 ↓	Inhibit proliferation and migration by directly or indirectly regulating TGF-β1 expression	^860^
	miR-34 ↓	Inhibit breast cancer migration and invasion through targeting Fra-1 oncogene	^861^
	miR-128 ↓	Inhibit cell motility and invasive capacity	^363^
Liver cancer	miR-10b ↑	Boosts the proliferation, migration, and invasion by targeting SLC38A2	^862^
	miR-93 ↑	Promote cell proliferation via targeting MAP3K2	^863^
	miR-15a-5p ↓	Inhibit cell migration, apoptosis, and growth by targeting E2F3	^864^
	miR-214 ↓	Impede DNA replication and tumorigenesis	^865^
Melanoma	miR-34a ↓	Led to downregulation of ULBP2, diminishing tumor cell recognition by NK cells	^866^
	miR-488-5p ↓	Inhibit proliferation, migration, anchorage independent growth and lead to induction of apoptosis	^867^
Gastric cancer	circCACTIN ↑	Promote proliferation, migration, invasion and EMT of gastric cancer by sponging miR-331-3p and regulating TGFBR1 expression	^379^
	circNHSL1 ↑	Promote cell mobility and invasion, as well as in vivo tumorigenesis and metastasis through the miR-1306-3p/SIX1/vimentin axis	^380^
	circFGD4 ↓	Suppress gastric cancer cell viability, colony formation, migration, induced EMT, and tumorigenesis and metastasis in vivo by modulating the miR-532-3p/APC axis to inactivate the β-catenin signaling.	^381^
	circRNA_0005529 ↑	Facilitate growth and metastasis of gastric cancer via regulating miR-527/Sp1 axis	^868^
Pancreatic cancer	circFOXK2 ↑	Promotes growth and metastasis by complexing with RNA-binding proteins and sponging miR-942	^390^
	circBFAR ↑	Promote proliferation, invasion, and migration via the miR-34b-5p/MET/Akt axis	^391^
	circRNF13 ↑	Promote proliferation, angiogenesis, invasion and glycolysis by acting as a miR-654-3p sponge	^869^
	circATG7 ↑	Accelerate cell proliferation and metastasis via miR-766-5p/ATG7	^870^
	circRREB1 ↑	Promote metabolic reprogramming and stemness maintenance	^871^
Lung cancer	circSLC25A16 ↑	Accelerate the glycolysis and proliferation via miR-488-3p /HIF-1α	^385^
	circTP63 ↑	Facilitate cell cycle progression by upregulating FOXM1	^386^
	circPRKCI ↑	Promote proliferation and tumorigenesis as a sponge for both miR-545 and miR-589	^387^
	circLIFRSA ↓	Inhibit cell growth and proliferation while promote apoptosis via the miR-1305/PTEN axis	^872^
	circNDUFB2 ↓	Inhibit growth and metastasis of cancer cells via destabilizing IGF2BPs and activating anti-tumor immunity	^258^
Breast cancer	circCDYL ↑	Promote autophagic level in BC cells via the miR-1275-ATG7/ULK1 axis	^383^
	circHSDL2 ↑	Promote the division, movement, and invasion of breast cancer cells act as a sponge for miR-7978 to affect ZNF704 expression	^384^
	circSEPT9 ↑	Promote the proliferation, migration and invasion, inhibit apoptosis and autophagy	^873^
	circRNF20 ↑	Promote the proliferation and Warburg effect (aerobic glycolysis) act as miR-487a sponge	^874^
	circBCBM1 ↑	Promote the proliferation and migration act as an endogenous miR-125a sponge	^875^
Liver cancer	circMTO1 ↓	Inhibit cell proliferation and invasion act as the sponge of miR-9	^376^
	circTRIM33-12 ↓	Inhibit tumor proliferation, migration, invasion and immune evasion act as the sponge of miR-191	^377^
	circRHOT1 ↑	Promote HCC growth and metastasis by initiation of NR2F6 expression	^378^
	circASH2 ↓	Inhibit HCC metastasis by altering tumor cytoskeleton structure	^876^
	circIPO11 ↑	Drive the self-renewal of liver CSCs and promote the propagation of HCC via activating Hedgehog signaling pathway	^877^
Gastric cancer	RUNX1-IT1 ↓	Inhibit cell invasion and migration by inhibiting the maturation of miR-20a	^878^
	H19 ↑	Promote EMT and metastasis via activating Wnt/β-catenin signaling	^879^
	LINC00152 ↑	Promote migration and invasion of cancer cells through activating ERK/MAPK signaling pathway	^880^
	UCA1 ↑	Promote cell proliferation and inhibit cell apoptosis via the miR-145/MYO6 axis	^881^
	CCAT5 ↑	Promote growth and metastasis via mediating Wnt/β-catenin signaling	^882^
Pancreatic cancer	LINC00941 ↑	Increase cell proliferation and metastasis by binding ANXA2 and activating FAK/AKT signaling	^883^
	CYTOR ↑	Promote cell proliferation and migration by modulating the miR-205-5p/CDK6 axis	^884^
	HOTAIR↑	Promote cell proliferation, alter cell cycle progression and inhibit apoptosis	^885^
	BCAN-AS1 ↑	Promote tumor growth and metastasis by stabilizing c-Myc	^886^
	NEAT1 ↓	Inhibit the proliferation, migration and invasion through spongy miR-146b-5p/traf6	^887^
Lung cancer	MALAT1 ↑	Promote cellular proliferation, EMT, and angiogenesis by acting as a ceRNA to sponge miRNAs	^338^
	ADPGK-AS1 ↑	Promote cancer cell growth by switching macrophage metabolic and phenotypic state	^888^
	MEG3 ↓	Affect the immunity and autophagy of NSCLC cells via regulating the miR-543/IDO signaling pathway	^340^
	SLCO4A1-AS1 ↓	Inhibit migration and invasion by sequestering the TOX4-NTSR1 signaling axis	^889^
	LINC01123 ↑	Promote proliferation and aerobic glycolysis through miR-199a-5p/c-Myc axis	^890^
Breast cancer	HOTAIR ↑	Promote cell proliferation, invasion and migration and inhibit apoptosis and G1 phase block	^891^
	ROPM ↑	Increase breast cancer stem cell properties via activating PI3K/AKT, Wnt/β-catenin, and Hippo/YAP signaling	^892^
	SPINT1-AS1 ↑	Increase proliferation and migration by sponging miR-let-7a/b/i-5p	^893^
	LINC02273 ↑	Promote cancer metastasis by epigenetically increasing AGR2 transcription	^894^
	PRBC ↑	Promote autophagy and progression of breast cancer through modulating PABPC1-mediated mRNA stabilization	^895^
Liver cancer	HULC ↑	Accelerate the growth of human liver cancer stem cells by upregulating CyclinD1 through miR675-PKM2 pathway via autophagy	^896^
	TLNC1 ↑	Enhance the growth and metastasis of hepatoma cells via inhibit p53 signaling	^897^
	TUG1 ↑	Promote migration, invasion, and glycolysis by regulating the miR-524-5p/SIX1 axis	^898^
	MINCR ↑	Promote proliferation and inhibit apoptosis by regulating microRNA-107/β-catenin	^899^
Gastric cancer	piR-651 ↑	Facilitate cell proliferation and invasion, restrained cell apoptosis and the percentage of arrested cells in G0/G1 phase	^408^
	piR-823 ↓	Inhibit tumor cell growth in vivo and in vitro	^407^
	PIWIL1 ↑	Promote cell proliferation, migration, metastasis, and tumorigenesis	^900^
Lung cancer	PMLCPIR ↑	Promote cell proliferation and decrease apoptosis	^901^
	piR-651 ↑	Influence cell proliferation, apoptosis, migration and invasion	^902^
	piR-55490 ↓	Inhibit the growth of lung carcinoma by suppressing mTOR signaling	^903^
	piR-211106 ↓	Inhibit the proliferation and promote the apoptosis through pyruvate carboxylase	^904^
Pancreatic cancer	piR-017061 ↓	Inhibit pancreatic cancer cell growth in vitro and in vivo	^905^
Breast cancer	piR-2158 ↓	Suppress cell proliferation, migration, epithelial-mesenchymal transition (EMT) and stemness	^906^
	piRNA-36712 ↓	Inhibit cancer cell proliferation, invasion and migration	^412^
	piR-YBX1 ↓	Inhibit proliferation and metastasis by the MAPK signaling pathway	^907^
	piR-651 ↑	Facilitate cell proliferation and invasion, restrain cell apoptosis and the percentage of arrested cells in G0/G1 phase	^408^
	piRNA-823 ↑	Promote cell proliferation and colony formation	^908^
Liver cancer	piR-017724 ↓	Inhibit the proliferation, migration and invasion by affecting the downstream protein PLIN3	^909^

#### Histone methylation and demethylation

Histone methylation is instrumental in the establishment and preservation of heterochromatin architecture, thereby mediating the repression of gene transcription. Histone methylation primarily occurs mainly on the arginine (Arg/R) and lysine (Lys/K) residues of histone H3 and H4. It is catalyzed by histone methyltransferases (HMTs), which transfer a methyl group from S-adenosylmethionine (SAM) to histones.^105^ HMTs primarily consist of histone lysine methyltransferases (HKMTs) and protein/histone arginine methyltransferases (PRMTs).^106^ The main KMTs include the SET1, SET2, MLL, Suv39, and EZH families. PRMTs can be categorized into three types based on their catalytic activity: Type I PRMTs (PRMT1, PRMT2, PRMT3, PRMT4, PRMT6, and PRMT8), Type II PRMTs (PRMT5 and PRMT9), and Type III PRMTs (PRMT7).^107^ Correspondingly, there were also histone demethylases have been identified, the enzymes of the histone-lysine demethylase group are divided into two main families: KDM1, which includes two members (LSD1/KDM1A and LSD2/KDM1B), and JmjC-containing HDMTs, which consist of several subfamilies with more than 30 proteins totally.^108^

Furthermore, a spectrum of studies has shown that alterations of histone methylation within various tumors are strongly linked to the prognosis and development of cancer. For example, H3K27me3 has been accessed as a prognostic indicator in patients with prostate, breast, ovarian, pancreatic and esophageal cancer.^109^ High levels of H3K27me3 correlate with poor prognosis in esophageal cancers.^110^ The variations in histone methylation levels, orchestrated by HMTs and demethylases, are frequently altered in tumors. Specifically, EZH2 was detected to be elevated in both primary and metastatic prostate cancers. Moreover, patients presenting with EZH2 overexpression showed a significantly lower survival rate compared to those having low EZH2 expression.^111^ KMT1C (G9a) is upregulated in breast cancer, colon cancer and gastric cancer,^112–114^ which is indicative of poor prognosis of various tumors. Besides, NSD1, NSD2, and NSD3 increase in glioblastoma bladder and prostate cancer.^115–117^ Histone demethylation enzymes are pivotal in tumor initiation and progression by modulating chromatin structure and gene expression. For instance, LSD1, a member of the histone-lysine demethylase family, exhibits overexpression in a wide range of human cancers, among which are leukemia and solid tumors. Similarly, JmjC domain-containing enzymes, another class of histone demethylases, are implicated in cancer pathogenesis. Overactivation of KDM4B has been noted in prostate cancer, while KDM4C overexpression was initially identified in esophageal, lung, and breast cancers.^118–120^ Additionally, the overexpression of KDM5B has been noted in prostate cancer. Interestingly, a single cancer type may exhibit dysregulation of multiple histone-lysine demethylases simultaneously. For example, in breast cancer, concurrent overactivation of HDMTs such as KDM5C, KDM5B, KDM4A, or KDM4B has been documented^121^ (Fig. 3). These results emphasize the elaborate relationship between histone methylation and cancer development, highlighting the potential of these enzymes as therapeutic targets for cancer treatment (Table 1).

#### Histone phosphorylation and dephosphorylation

In contrast to histone methylation, histone phosphorylation facilitates the unwinding of chromatin architecture and promotes gene transcription. In addition, histone phosphorylation is essential for chromosome condensation and segregation during mitosis. This dynamic and reversible modification occurs at serine, threonine, and tyrosine residues and is mediated by protein kinases. This process is distinct from the more stable methylation marks, as phosphorylation is transient, inducible, and often specific to particular pathways.^122^ Kinases such as PKA, PKC, AMPK, and JAK2 can add phosphate groups to proteins of histone, thereby influencing chromatin architecture and gene transcription. Conversely, histone dephosphorylation is carried out by phosphatases, including PP1, PP2A, PP2B (calcineurin), PP2C, PP4, PP5, PP6, and PP7. The readers of these phosphorylated histone marks primarily consist of 14-3-3 proteins and BRCT domain-containing proteins, which recognize and respond to the phosphorylation status of histones, further modulating cellular signaling and transcriptional regulation.^123^

The changes in histone phosphorylation in tumors mainly involve the H3S10 site and related enzymes. Elevated levels of H3S10ph, a phosphorylation marker at serine 10 of histone H3, have been identified in a wide range of cancers including invasive breast cancer, esophageal squamous cell carcinoma, gastric carcinoma, spongioblastoma, melanoma, and nasopharyngeal cancer. The presence of increased H3S10ph is not only more frequent in these cancers but also correlates with a worse prognosis, highlighting its potential as a clinical indicator.^124–127^ Besides, many H3S10 kinases, including MSK1/2, PIM1, CDK8, and AURORA kinases, are overexpressed in various types of cancer.^128^

#### Histone ubiquitination

As mentioned before, methylation, acetylation and phosphorylation modifications add small chemical groups to histones. In contrast, ubiquitination covalently attaches a larger 76-amino acid ubiquitin molecule to histone.^129^ The process of ubiquitination begins with the activation of ubiquitin by ubiquitin-activating enzymes (E1s), which transfer the activated ubiquitin to ubiquitin-conjugating enzymes (E2s). Finally, ubiquitin is transferred from E2 to the substrate by ubiquitin ligases (E3s).^130^ The E3 ligase include RNF168, RNF8, RING1A/1B, BMI1 and BRCA1/BARD1. And deubiquitinating enzymes contain USP3 USP44, BRCC36, BAP1 and MYSM1.^131^

In cancer, the process of histone ubiquitination often goes awry, which may result in alterations in the transcription of tumor suppressor genes and carcinogenic genes, thereby facilitating the proliferation and differentiation of cancer. For instance, the level of H2BK120ub1 is frequently reduced in tissues of breast cancer, lung cancer, and colorectal cancer compared to normal tissues.^132,133^ The widespread reduction of H2BK120ub1 is observed in approximately 70% of primary breast and colon cancer specimens, and is linked to a worse prognosis.^134^ Furthermore, the expression of enzymes associated with histone ubiquitination is also dysregulated in tumors. BMI1 exhibits elevated expression levels and facilitates the self-renewal capacity of cancer cells in acute myeloid leukemia (AML) and various solid tumor malignancies. Besides, RNF20 and RNF40 have been observed to be downregulated in seminoma, basal-like breast cancer, and colorectal cancer.^135–137^ And USP22 was found to overexpressed in prostate cancer.^138^ These investigations reveal that enzymes associated with histone ubiquitination are aberrantly regulated across diverse tumor types and exhibit a strong correlation with tumor proliferation, advancement, and metastatic dissemination. These observations underscore the intricate involvement of histone ubiquitination in oncogenesis and suggest its promising utility for therapeutic interventions and prognostic evaluation (Fig. 3).

#### Histone lactylation, citrullination and crotonylation

Histone lactylation attenuates the chromatin compaction facilitating the attachment of transcription factors and enhancing gene transcription, thereby modulating a series of physiological activities such as embryogenesis, cell metabolism and signal transduction. Histone lactylation refers to the process by which lactic acid modifies the lysine residues on histones, a post-translational modification known as lysine lactylation (Kla or Klac). Histone lactylation is linked to numerous physiological and pathological processes, including pulmonary fibrosis, tumors, cardiovascular diseases. We will elucidate the role of histone lactylation in tumorigenesis. Recently, alanyl-tRNA synthetase (AARS1) was found to function as a lactate sensor that modulates global lactylation and increases the lactylation of p53, contributing to tumorigenesis.^139^ Moreover, there is a study indicated that extent of histone lactylation is connected to an unfavorable prognosis for those suffering from clear cell renal cell carcinoma (ccRCC).^140^ Studies have shown that an increase in histone lactylation promotes liver metastasis of colorectal cancer cells. Furthermore, the level of histone lactylation is also increased in prostate cancer and lung adenocarcinoma.^21,141^ In ocular melanoma, the increase in histone lactylation leads to enhanced proliferation and migration of ocular melanoma cells by promoting the transcription of the m^6^A-modified recognition protein YTHDF.^141^ Histone lactylation not only promotes cancer but also acts as tumor suppressors in some contexts. In non-small cell lung cancer (NSCLC), the increased level of histone lactylation leads to the inhibition of glucose uptake and glycolysis, as well as the reduction of cell proliferation and migration. In uveal melanoma, histone H3K18la modifies nuclear enlargement and induces cell cycle arrest.^142^ The progression of UM was further inhibited. As for histone lactylation-related enzymes, recent studies have shown that SIRT2, a deacetylase, can inhibit the proliferation and migration of neuroblastoma cells.^143^ Besides, SIRT3 has the ability to impede the proliferation of HCC cells by modulating the level of Cyclin E2 lactate modification^144^ (Fig. 3).

Histone citrullination modulates gene expression by diminishing the hydrogen bond complement within chromatin, leading to chromatin decondensation, and it exerts a pivotal influence on cellular division, programmed cell death and tumor progression. Moreover, histone citrullination a post-translational modification mediated by the peptidyl-arginine deiminase (PAD) enzyme family, entails the conversion of arginine residues within histones to citrulline.^145,146^ This modification involves the genesis of neutrophil extracellular traps (NETs) and is closely related to tumors. In cancer, histone citrullination is linked to the tumor microenvironment, proliferation, metastasis, and drug resistance of cancer cells. PAD2, PAD4 and citrullinated histones are highly expressed in prolactinomas. PAD4 exhibits substantial expression in a range of malignant tissues, however, it is notably absent or present at significantly lower levels in both normal tissues and benign tumors.^147^ Furthermore, heightened levels of PAD4 have been detected in a multitude of solid malignancies and concomitantly noted to be upregulated in the peripheral blood of individuals afflicted with lung carcinoma^148^ (Fig. 3).

Significantly, histone crotonylation induces a heightened relaxation of chromatin architecture and exerts a more potent stimulatory effect on gene expression compared to acetylation. Furthermore, the equilibrium between histone crotonylation and acetylation, as well as other acylation modifications, imparts functional implications on gene expression. Thereby, histone crotonylation plays a pivotal role in the developmental trajectory of embryonic stem cells, cellular metabolism, the DNA damage response, and tumor progression. Histone crotonylation refers to the addition of a crotonyl group to a lysine residue. Histone crotonylation was discovered in 2011.^149^ The function of histone crotonylation has been thoroughly investigated in the last decades and is closely related to the transcription and replication of genes.^150–152^ This modification plays a role in a variety of biological processes, including gene expression regulation, cell signaling and is related to the occurrence and development of a variety of diseases, especially cancer.^153–155^ Histone crotonylation is intricately linked to the etiology, progression, metastasis, and therapeutic response of tumors.^156,157^ Recently, the p300-regulated lysine crotonylome was characterized by a quantitative proteomics study, which also showed that p300-targeted Kcr substrates are potentially linked to cancer.^158^ This implies that crotonylation could serve as an oncogenic factor that advances tumor progression. In the EDRN database, 4.5% of tumor biomarkers have been identified as crotonylated, and 32 crotonylated proteins are connected with tumor genes.^159^ Histone crotonylation is also associated with metabolic regulation in diverse types of tumors (Fig. 3). For instance, levels of histone crotonylation are decreased in liver, gastric, and renal cancers, while they are increased in thyroid, esophageal, pancreatic, and lung cancers. Elevated levels of crotonylation can inhibit the motility and proliferation of liver cancer cells.^160^ Collectively, the aberrant expression of histone post-translational modifications mentioned above in various cancers holds significant impact on tumor progression. As the research spectrum broadens, the biological functions of these novel modifications and their roles in tumors are gradually being revealed, providing novel perspectives and potential candidates for tumor diagnosis and treatment (Table 1).

### DNA methylation

#### 5-mC

5mC represents a chemical modification on DNA and constitutes the most prevalent form of DNA methylation.^161^ The process of DNA methylation is catalyzed by a family of DNA methyltransferases (DNMTs), with these enzymes transferring a methyl group from SAM to the fifth carbon of a cytosine, thereby giving rise to 5mC.^162^ DNMT3A and DNMT3B have the ability to form a new methylation pattern to unmodified DNA and are hence known as de novo Dnmt.^163^ In mammals, DNMT3A and DNMT3B are the enzymes responsible for de novo methylation, whereas DNMT1 primarily maintains the methylation pattern during the process of DNA replication.^164^ The proteins involved in erasing DNA methylation primarily encompass the TET family proteins (TET1, TET2, and TET3).^165^ Meanwhile, “readers” refer to proteins capable of recognizing and binding to methylated DNA, such as the MBD protein family, the UHRF (ubiquitin-like with PHD and RING finger domains) protein family, and proteins containing zinc finger domains. These proteins contribute to the regulation of gene expression by binding to methylated DNA.^166^ In the following text, we will delve into the specific details regarding the functions and roles of these enzymes and proteins involved in DNA methylation, as well as their implications in cancer development.

#### DNMT1

DNMT1 serves as a crucial enzyme in the maintenance of the genome’s methylation patterns, and its dysregulated expression is intimately linked to the occurrence and development of a diverse range of malignancies. In numerous types of cancer, including breast cancer, lung cancer, colorectal cancer, pancreatic cancer, gastric cancer, and cervical cancer, the expression of DNMT1 is frequently upregulated,^167^ and this upregulation is associated with the enhancement of tumor cell proliferation, migration, and invasive potential. For example, in breast cancer, DNMT1 promotes the occurrence and development of tumors by suppressing the expression of tumor suppressor genes through methylation.^168^ In lung cancer, the inhibition of DNMT1 can reduce cell proliferation and increase apoptosis, and its activity is related to the invasiveness and metastatic potential of tumors.^169^ In CRC, the overexpression of DNMT1 is related to the progression of tumors and may be associated with the invasiveness and metastatic potential of tumors.^170^ The inhibition or downregulation of DNMT1 exerts a significant anti-cancer effect in certain tumors. Overall, DNMT1 plays a multifaceted and intricate role on the pathogenesis and progression of malignancies, including processes such as cellar proliferation, apoptosis, invasion, and metastasis. Crucially, the dysregulation of DNMT1 expression can confer resistance to various treatment modalities by promoting cancer stem cell properties, such as the aforementioned malignant phenotypes. For instance, DNMT1 downregulates FOXO3a, thereby enhancing the chemoresistance of breast cancer stem cells.^168^ In conclusion, DNMT1 conspicuously emerges as a pivotal factor in tumorigenesis and tumor progression, with its profound and far-reaching impacts on multiple aspects of cancer biology. Given its capacity to confer treatment resistance as well, further in-depth investigations into DNMT1 are both necessary and justified to explore potential therapeutic strategies for more effectively managing and combating various cancers (Fig. 3).

#### DNMT3A

DNMT3A, a highly significant enzyme, assumes a crucial role in the regulation of gene expression. It undertakes the responsibility of appending methylation marks onto DNA molecules, thereby exerting a regulatory influence on gene activity. DNMT3A occupies a central position in the origin and progression of a broad spectrum of cancers, with its impact being particularly pronounced in AML. In the context of AML, DNMT3A manifests a notably high mutation frequency, which is closely intertwined with an unfavorable prognosis and resistance to therapeutic interventions.^171^ Abnormal expression of DNMT3A in tumors is usually characterized by upregulation, which is associated with enhanced abilities of tumor cells to proliferate, migrate, and invade. For example, in AML, mutations in DNMT3A can result in altered de novo DNA methylation patterns, affecting gene expression and promoting the occurrence and development of leukemia.^172^ In NSCLC, increased expression of DNMT3A is associated with the tumor’s potential for invasion and metastasis, and its inhibition can reduce cell proliferation and increase apoptosis.^173^ In colorectal cancer, overexpression of DNMT3A is associated with the activation of MEK/ERK signaling pathway, leading to malignant characteristics such as high invasiveness and mobility of the tumor.^174^ The abnormal activity of DNMT3A can also lead to resistance to cancer treatment.^175^ Given its role in tumors, DNMT3A has emerged as a prime target for cancer treatment. Treatment strategies centered around DNMT3A, such as the application of DNMT3A inhibitors, may well constitute a novel direction for future cancer therapy.

#### DNMT3B

Similar to DNMT3A, DNMT3B also plays a significant part in the de novo synthesis of DNA methylation.^176^ The deviant expression of DNMT3B is related to the progression and resistance phenotype of various malignant tumors (Fig. 3). In a variety of tumors, such as breast cancer, lung cancer, colorectal cancer, pancreatic cancer, gastric cancer, and cervical cancer, the expression of DNMT3B is often upregulated,^177^ and this upregulation is associated with enhanced tumor cell proliferation, migration, and invasive capacity.^178^ For example, in AML, mutations in DNMT3B can lead to changes in de novo DNA methylation patterns, affecting gene expression and boosting the occurrence and development of leukemia.^179^ However, in some cases, the downregulation or loss of function of DNMT3B is also related to the occurrence of tumors. I In a mouse model with a knockout of the *Dnmt3b* gene, the absence of DNMT3B accelerates the development of lymphoma, indicating that DNMT3B may act as a tumor suppressor gene in normal cells, inhibiting tumor development by stabilizing DNA methylation patterns.^180^ It is worth highlighting that the abnormal expression of DNMT3B varies among different types of cancer, which implies that both its overexpression and silencing can exert an impact on gene expression. In summary, DNMT3B plays a multifaceted and complex role in the occurrence and development of tumors, and interventions targeting DNMT3B may potentially constitute a novel direction for tumor treatment.

#### TETs

TET proteins constitute a category of enzymes that play a crucial role in oxidizing 5mC to 5-hydroxymethylcytosine (5hmC) and subsequent oxidation products, thereby indirectly facilitating DNA demethylation. Aberrations in DNA methylation patterns represent a defining characteristic of cancer. The activity and expression of TET enzymes, which is involved in removing this epigenetic mark, has also emerged as an important tumor suppressor mechanism in cancer.^181^ The functionality and expression of TET enzymes, which play a pivotal role in the removal of these epigenetic modifications, have also been recognized as a crucial tumor-suppressive mechanism in oncogenesis. Diminished expression of TET proteins and decreased levels of 5hmC are prevalent features across number cancer types, encompassing gastric carcinoma, prostate cancer, hepatocellular carcinoma, pulmonary neoplasms, and breast cancer, as well as glioblastoma multiforme and cutaneous melanoma.^182–185^ For example, mutations in the *TET2* gene are associated with a poor prognosis for AML, and low expression or inactivation of *TET2* is common in a variety of tumors, while activation of *TET2* can suppress tumor development.^186^ Besides, under certain circumstances, high expression of TET proteins may increase chemoresistance. For instance, in ovarian cancer, high expression of TET proteins may enhance chemoresistance, and elevated expression levels of TET1 contribute to chemoresistance, possibly by modulating the DNA damage response and repair systems.^187^ Interestingly, tumors can acquire resistance to DNMT inhibitors by modulating the expression of TET proteins. Deletion of the DNMT1 gene rendered cancer cells susceptible to TET2 upregulation following exposure to DNMT inhibitors. This elevation in TET2 expression coincides with the development of resistance to DNMT inhibitors under conditions of DNMT1 deficiency.^188^ The aforementioned results suggest that mutations in TET proteins and alterations in their expression can lead to the epigenetic disruption of 5hmC and 5mC patterns. Nevertheless, the exact influence of altered TET activity on the initiation, advancement, and sustenance of these malignancies remains predominantly enigmatic and represents a domain that is presently under vigorous scientific investigation.

#### MBD proteins

The MBD proteins family stands out as primary contenders for deciphering DNA methylation, as they can recruit chromatin remodelers, HDACs, and methylases to methylated DNA, which participates in gene repression.^189^ The MBD protein family includes multiple members, such as MBD2 and MBD3, which affect gene expression by regulating DNA methylation and histone modifications to, thereby participating in the formation and progression of tumors.^190^ MBD2 exhibits significant differences in expression levels and functions across different types of cancer (Fig. 3). In lung and breast cancer, MBD2 is highly expressed and closely related to tumor progression and metastasis.^191^ In gastric cancer, however, MBD2 expression is reduced, and its downexpression may affect the biological characteristics of the tumor.^192^ Additionally, MBD3 plays a significant role in the occurrence and development of cancer, with higher expression in liver cancer tissue compared to adjacent normal liver tissue, and its expression level is negatively correlated with patient prognosis, such as overall survival, disease-free survival, and metastasis-free survival.^193^ This suggests that high expression of MBD3 may be associated with the development of liver cancer and a worse prognosis. Mutations in the MBD4 gene heighten the risk and intricacy of various cancers by affecting DNA repair mechanisms, increasing mutational burden, and promoting the formation of specific mutational spectra.^194^ Given the important role of the MBD protein family in tumor development, targeting MBD proteins for treatment may represent a new direction for future cancer therapy.^166^ Through in-depth investigations into the expression patterns and functions of MBD proteins in different cancer types, scientists can devise more precise diagnostic tools and treatment approaches aimed at curbing tumor growth and dissemination by capitalizing on the specific mechanisms of these proteins.^195^ Furthermore, comprehending how MBD proteins impact DNA repair and mutational spectra can assist us in gaining a better understanding of the genetic underpinnings of tumors and furnishing new strategies for cancer prevention and treatment.

### RNA modifications

#### N6-methyladenosine (m^6^A)

In RNA molecules, m^6^A, a crucial epigenetic modification, involves the addition of a methyl group to the nitrogen atom at the sixth position of adenosine. This modification is prevalent among diverse various RNA species, particularly in the 3’-untranslated regions (3’-UTRs) and near mRNA stop codons.^196^ The core methyltransferase complex, often referred to as the “writer,” is composed of methyltransferase-like 3 (METTL3) and methyltransferase-like 14 (METTL14), with METTL3 acting as the catalytic subunit. This complex is supported by additional proteins, including Wilms tumor 1-associated protein (WTAP), VIR-like m^6^A methyltransferase associated (VIRMA), RNA-binding motif protein 15 (RBM15), and zinc finger CCCH-type containing 13 (ZC3H13), which contribute to the co-transcriptional deposition of m^6^A on nascent pre-mRNAs. Demethylation, the reversal process, is carried out by enzymes known as “erasers,” such as Fat mass and obesity-associated protein (FTO) and AlkB homolog 5 (ALKBH5).^197^ The expression of m^6^A regulators is frequently dysregulated in cancer, and they exert significant influence on cancer development and progression.

##### METTL3

Undoubtedly, METTL3 is the most studied type of m^6^A writer enzyme, which is active in complex with METTL14 and the splicing regulator WTAP.^198^ Emerging research has demonstrated that METTL3 enhances tumor proliferation and metastasis. METTL3 mRNA and protein exhibit substantial expression levels in acute myeloid leukemia.^199^ In bladder cancer, METTL3 is significantly overexpressed in patient-derived samples and facilitates the advancement of bladder cancer through the AFF4/NF-κB/MYC signaling cascade.^200^ In addition, Studies have documented that METTL3 is markedly elevated in HCC and correlates with reduced overall survival in HCC patients.^201^ Moreover, evidence indicates that elevated METTL3 expression is linked to advanced pathological stages in pancreatic ductal adenocarcinoma (PDAC).^202^ And its function as a tumor suppressor has also been established in distinct subgroups of breast and colorectal cancers as well as glioblastomas.^203,204^ In summary, the expression of METTL3 in tumors is complex, and it may play different roles in various types of cancers (Fig. 3), acting as an oncogene to promote tumor development in some cases, and as a tumor suppressor in others. These observations imply that METTL3 represents a promising therapeutic target for oncological interventions, but its exact role and mechanisms in cancer treatment require further research and exploration.

##### METTL14

The expression of METTL14 patterns closely mirror those of METTL3, and it has been implicated in playing dual roles as both an oncogene and a tumor suppressor.^198^ More studies have demonstrated the role of METTL14 as a tumor suppressor.^205–207^ Downregulation of METTL14 promotes cancer cell growth, invasion, migration and forecasts poor prognosis in patients with HCC and CRC contributes to progression and metastasis.^208–210^ Besides, the expression of METTL14 was observed to be elevated in CRC tissues, and survival analysis revealed that the METTL14 expression level had a significantly link to the improved prognosis of CRC. Similarly, METTL14 has been proposed a potential indicator for the diagnosis and prognosis of endometrial cancer.^49,211^ In the tissue samples of gastric cancer (Fig. 3), METTL14 was found to be under-expressed. This low expression of METTL14 played a role as a prognostic factor associated with poor survival in those suffering from gastric cancer.^210^ Moreover, the knockout of METTL14 is capable of triggering the Wnt and PI3K - Akt signaling, which in turn promotes the growth and invasion of gastric cancer cells.^212^ On the other hand, METTL14 plays an oncogenic role in stimulating the development and progression of tumors in some cases.^213–215^ The overexpression of METTL14 decreases PERP mRNA and protein levels and enhances tumor cell migration and colony formation.^214^ In addition, METTL14 mediates the expression of downstream genes CXCR4 and CYP1B1, thus facilitating tumor growth and development.^216^ The expression of METTL4 in tumors is complex, contributing to tumor development, metastasis, and metabolism, and it may become a potential target for cancer therapy.

##### FTO

The tumorigenic function of FTO in malignancies r was initially established in research on melanoma, wherein particular FTO variants were linked to an elevated risk of developing melanoma.^217^ Over the past few years, the involvement of FTO in oncogenesis has also been progressively explored. The upregulation of FTO is connected to enhanced tumor proliferation and progression, which is attributed to its capacity to diminish m^6^A methylation in oncogenes. This reduction in m^6^A residues stabilizes oncogenes, thereby promoting the translation of proteins that contribute to tumorigenesis.^218^ Recent studies on gastric cancer have shown that FTO was also highly expressed in the tumor region and promotes the occurrence of gastric cancer by promoting the proliferation, migration and lymph node metastasis of gastric cancer cells. Decreased expression of FTO protein is related to poor clinical outcomes in gastric cancer patients, suggesting that FTO exerts a regulatory influence on the progression and metastatic dissemination of gastric cancer.^219^ FTO is elevated expression in NSCLC tissues and cellular models, while m^6^A content is reduced.^220,221^ On the other hand, the antitumor effects of FTO have been gradually explored. Expression of FTO is reduced in melanoma.^222^ And upregulation of FTO overexpression inhibits CSC proliferation and tumorigenic potential in vitro cultures and in vivo xenograft murine models.^223–225^ Besides, in ovarian cancer, FTO is downregulated and promotes tumorigenesis and self-renewal by affecting the m^6^A modification levels of specific genes.^223^ The role of FTO may vary in different tumors, and additional investigations are required to delineate its specific mechanisms of action in tumor development.

##### ALKBH5

The dual function of ALKBH5 in modulating tumor growth is evident in colon cancer, lung cancer, renal cell carcinoma, and osteosarcoma, highlighting the complex nature of each m^6^A regulator in disease onset and progression.^203,226^ ALKBH5 exhibits high expression levels in hepatocellular carcinoma (HCC), gastric cancer and breast cancer (Fig. 3). Moreover, the upregulation of ALKBH5 is associated with a worse prognosis in patients with these types of cancer. Additionally, ALKBH5 contributes to poor survival rates in glioblastoma multiforme (GBM) by regulating ADAM19 and the transcription factor FOXM1.^227,228^ Besides, in bladder cancer, osteosarcoma, and multiple myeloma, the expression of ALKBH5 is also upregulated, primarily promoting the development and progression of tumors by affecting m^6^A modification.^229–231^ Oppositely, ALKBH5 is downregulated in prostate cancer and is pivotal in regulating the Wnt signaling pathway. It achieves this by decreasing the RNA methylation of Wnt inhibitory factor 1 (WIF-1), thereby inhibiting the tumorigenesis of PC.^232,233^ Moreover, reduced levels of ALKBH5 are correlated with an unfavorable prognosis in PDAC.^234^ In summary, ALKBH5 plays a multifaceted role in cancer, with its expression levels and impact on tumorigenesis varying across different cancer types. Its ability to modulate the m^6^A modification pathway and influence key signaling mechanisms, such as the Wnt pathway, underscores the complexity of its function in both promoting and suppressing tumor growth, depending on the cancer context. The prognostic significance of ALKBH5 is further highlighted by its association with patient outcomes in various malignancies.

##### YTHDF1

YTHDF1 has been identified as an oncogenic factor in various types of cancer. Comprehensive analysis from The Cancer Genome Atlas (TCGA) and Gene Expression Omnibus (GEO) databases, along with immunohistochemical studies, have revealed that YTHDF1 is consistently overexpressed in liver and breast cancers, correlating with poorer overall survival rates and advanced pathological stages.^235,236^ Recent research has extended these findings, demonstrating upregulation of YTHDF1 in melanoma and ovarian cancer as well.^237–239^ This protein stands out as an independent prognostic marker, being of vital importance in the regulation of cell cycle progression and metabolic pathways in liver cancer.^240^ The primary mechanism through which YTHDF1 fosters tumorigenesis involves facilitating the translation for m^6^A-modified mRNAs that are crucial for cell proliferation and the epithelial-mesenchymal transition (EMT).^241^ In the context of CRC, YTHDF1 has been shown to enhance the initiation and progression of the disease by activating the Wnt/β-catenin signaling pathway.^237,242,243^ These insights underscore the value of YTHDF1 as a probable therapeutic target in cancer treatment strategies.

##### YTHDF2

YTHDF2 exhibits a complex and context-dependent role in cancer biology, with its function varying across different types of research (Fig. 3). In the context of colon cancer, high levels of YTHDF2 are associated with increased malignancy, as it enhances the expression and translation of mRNAs that drive cell proliferation and invasion.^241^ In HCC, YTHDF2 expression is elevated and closely linked to the severity of the disease.^244^ Besides, in lung cancer, YTHDF2 interacts with the m^6^A modification site on the 3’-untranslated region (3’-UTR) of 6-phosphogluconate dehydrogenase (6PGD) mRNA, thereby promoting 6PGD mRNA translation and contributing to the proliferation of lung cancer cells.^245^ Conversely, in esophageal cancer, YTHDF2 is typically under expressed. Interestingly, when YTHDF2 is upregulated in esophageal and gastric cancers, it suppresses neoplastic cell proliferation and triggers programmed cell death, indicating a protective role against these malignancies.^246,247^ These findings highlight the dual nature of YTHDF2 in cancer, where its role as a promoter or suppressor of cancer progression is profoundly contingent upon the specific tumor subtype and cellular milieu. Understanding these nuances is crucial for formulating targeted strategies that can exploit the differential effects of YTHDF2 in cancer treatment.

##### YTHDF3

YTHDF3 is of significance in promoting the translation of oncogenic genes within diverse cancers. In CRC, YTHDF3 is notably overexpressed and has been linked to poorer patient survival outcomes.^236,248^ Its function in breast cancer is characterized by the m^6^A-dependent promotion of oncogene translation. Additionally, YTHDF3 also enhances the stability of m^6^A-modified ZEB1 mRNA, thereby facilitating metastasis of liver cancer.^244^ Moreover, YTHDF3 is frequently overexpressed in HCC, with higher expression levels correlating with an increased risk of cancer recurrence in patients.^249^ Furthermore, recent research has implicated YTHDF3 in tumorigenesis. It has been revealed to promote the translation of Integrin subunit α 6 (ITGA6) mRNA, which is instrumental in the development of bladder cancer.^250,251^ In the context of pancreatic cancer, YTHDF3’s role is further underscored by its interaction with ZDHHC20, which mediates S-palmitoylation of YTHDF3. This post-translational modification stabilizes MYC mRNA, thereby contributing to the progression of pancreatic cancer.^252^ These findings highlight YTHDF3 as a key factor in the regulation of cancer development and progression, underscoring its potential as a therapeutic target.

#### N1-methyladenosine(m^1^A)

The significance of N1-methyladenosine (m^1^A) modifications in cancer progression and prognosis has garnered increasing attention in recent research. The regulators associated with m^1^A modifications encompass a range of proteins, including TRMT6/TRMT61A, ALKBH1, ALKBH3, and the YTH-domain family proteins.^253^ Emerging evidence suggests that the levels of m^1^A methylation and the expression of m^1^A-related RNAs could serve as innovative biomarkers for predicting cancer prognosis. In CRC, the content of m^1^A is notably higher in patients compared to healthy individuals, indicating its potential diagnostic and prognostic value.^254^ To assess the m^1^A modification profile personalized patient contexts, the m^1^A score has been developed. In cervical cancer and oral squamous cell carcinoma (OSCC), patients exhibiting a high m^1^A score have been observed to increased lymphatic invasion, reduced survival, and a poorer response to immunotherapy.^255^ The m^1^A level has demonstrated the potential to predict the prognosis of various cancers. Above results highlight the importance of m^1^A modifications within the complex landscape for cancer biology and their prospective utility as therapeutic targets and prognostic indicators.

##### TRMT6/TRMT61A

The TRMT6/61A complex is of great significance in a variety of biological systems, especially in the context of cancer. Current research indicates that TRMT6/61A is upregulated in bladder cancer and is associated with an increase in m^1^A modification levels.^256^ Additionally, TRMT6/61A has been found to enhance the proliferation of gastrointestinal and breast cancer cells, exerting an oncogenic effect.^257–260^ TRMT6 exhibits an upregulated trend in certain types of cancer, particularly bladder cancer, and is associated with the degree of malignancy and the unfolded protein response. These findings highlight the potential role of TRMT6 in tumor development and suggest that it may serve as a target for future cancer treatments.

##### ALKBH1

When exploring the role of ALKBH1 across different cancers, we have found that expression levels of ALKBH1 have a significant correlation with patient prognosis. There is a significant negative correlation between the overexpression of ALKBH1 and overall survival rates in CRC. Recent research has highlighted that ALKBH1 is not only overexpressed in CRC but also plays a pivotal role in cancer metastasis.^261^ Besides, ALKBH1 typically acts as an oncogene, promoting tumorigenesis, as evidenced in glioma and gastric cancer.^261^ Furthermore, in lung cancer, the upregulation of ALKBH1 in both tissue and cellular contexts has been shown to enhance cell invasion and migration.^262^ However, in the context of pancreatic cancer, low levels of ALKBH1 expression are associated with a particularly poor prognosis^263^ (Fig. 3). As our comprehension of the mechanisms through which ALKBH1 operates in diverse cancers deepens, new therapeutic strategies may be developed in the future to target the expression and function of ALKBH1, thereby improving patient treatment outcomes (Table 1).

##### ALKBH3

Recent progress has revealed ALKBH3’s function as a cancer-promoting factor in various cancers. In prostate cancer, ALKBH3 serves as a critical biomarker for early detection and histopathological classification. Numerous studies have demonstrated that ALKBH3 expression level in prostate cancer are significantly elevated.^264,265^ Besides, ALKBH3 has been identified as a facilitator of neoplastic cell proliferation in gastrointestinal (GI) malignancies.^257,258^ Heightened the expression of ALKBH3 in lung adenocarcinoma (LUAD) has been correlated with post-recurrence survival.^266^ ALKBH3 enhances the glycolytic activity of neoplastic cells by modulating the expression of m^1^A-modified ATP5D mRNA.^267^ ALKBH3 has been documented to modulate the cell cycle.^268^ Moreover, Silencing of ALKBH3 induces the senescence and halts the cell cycle in lung cancer and urothelial carcinomas by upregulating the expression of cell cycle arrest proteins p27 and p21.^266^ ALKBH3 could produce tRNA-derived small RNAs (tDRs) through demethylation of m^1^A-tRNA thereby enhancing the growth and invasive potential of cancer cells.^269^ In summary, the multifunctionality of ALKBH3 in cancer development suggests that it may become a potential target for future cancer therapies, particularly in strategies that target its expression and function.

#### 5-Methylcytosine (m^5^C)

In the context of cancer, the m^5^C modification is intricately linked to the proliferation, migration, invasion, and therapeutic resistance of tumor cells.^270,271^ The biological function of m^5^C is closely associated with the proteins that modulate their presence: the writers, erasers, and readers. M^5^C writers, such as DNMT2 and the NSUN family proteins, are responsible for the establishment of methylation marks. Readers, including ALYREF and YBX1, are proteins that identify and attach to these methylation sites. Conversely, m^5^C erasers, such as the TET family proteins and ALKBH1, are involved in the removal of these methylation marks, creating a dynamic equilibrium between the two opposing processes.^272^ The role of m^5^C RNA modification in gastric cancer is predominantly oncogenic, with elevated m^5^C levels being indicative of a poor prognosis and reduced overall survival rate.^273^ The oncogenic impact of m^5^C modulation may also extend to immune suppression, as gastric cancer patients with lower levels of m^5^C modulation levels have been observed to exhibit higher immune activity, along with longer progression-free survival and overall survival.^273^ These insights highlight the complex interaction between m^5^C modification and cancer biology, highlighting its potential as a therapeutic target and prognostic marker. Additionally, absence of DNMT2 is associated with alterations in mRNA expression and methylation patterns and the suppression of cell proliferation and migration.^274^

M^5^C-related enzymes are frequently dysregulated in tumors, and the NSUN family proteins, which consists of NSUN1-7, plays a significant role in this context.^275^ Recent investigations have demonstrated that NSUN3 expression is significantly elevated in individuals diagnosed with low-grade glioma and head and neck squamous cell carcinoma (HNSCC). Moreover, it has been elucidated that NSUN3-facilitated m5C methylation of tRNA potentiates metastatic progression by augmenting the translational efficiency of mitochondrial mRNA.^276^ The immune cell infiltration associated with NSUN3 predominantly encompasses CD8^+^ T lymphocytes and M2-polarized macrophages,^273,277,278^ which suggests its role in modulating the tumor microenvironment. High levels of m^5^C were negatively related to prognosis of patients with glioma.^279,280^ Besides, overexpression of NSUN5 has been associated with tumorigenesis in HCC.^281^ Research has suggested that NSUN6 functions as a protective agent against triple-negative breast cancer (TNBC), pancreatic carcinoma, testicular cancer, thyroid malignancies, and ovarian cancer, while serving as a risk factor for CRC.^282–285^ These findings underscore the complex and context-dependent roles of NSUN family proteins in the biology of various cancers.

The m^5^C demethylase identified to date include the TET family proteins and ALKBH1. TET2 is primarily responsible for catalyzing the conversion of m^5^C to hm^5^C, thereby facilitating the removal of m^5^C modifications in RNA.^286^ Studies have measured TET2 expression across a spectrum of cancer types, revealing its upregulation in patients with low-grade glioma.^287^ In contrast, TET2 expression is reduced in clear cell renal cell carcinoma (ccRCC), ovarian cancer, and prostate adenocarcinoma.^288,289^ The specific role of TET3 in mediating m^5^C elimination remains to be fully elucidated. Nonetheless, some research has suggested that elevated TET3 expression in prostate cancer may correlate with a poorer prognosis, emphasizing the intricate and context-dependent roles of TET enzymes in the realm of cancer biology.^290^ Additionally, research has indicated that ALKBH1 is upregulated in a variety of cancers, including gastric, head and neck, and liver cancers. High expression levels of ALKBH1 have been associated with poor prognosis in multiple tumor types. Moreover, ALKBH1 is markedly overexpressed in advanced tumors that are high metastatic, and exhibit high malignancy, underscoring its potential role as a biomarker for aggressive cancer behavior.^291^

Readers, or proteins that bind to m^5^C sites, include ALYREF and YBX1. ALYREF has been identified as a significant oncogenic factor, correlating with poor prognosis in patients across various cancer types, such as HCC, glioblastoma, glioma, and neuroblastoma.^292–294^ In HCC patients, increased levels of ALYREF are associated with the upregulation of eIF4A3 expression, as well as disruptions in the cell cycle and mitosis.^295^ Within the context of lung adenocarcinoma, ALYREF, in conjunction with NSUN2, enhances the m^5^C modification of Yes-Associated Protein (YAP) mRNA, a factor whose high expression in tumors is linked to increased invasiveness and metastatic potential.^296–299^ YBX1, another m^5^C reader, plays a multifaceted role in cancer development, with its oncogenic effects observed in gastric cancer, bladder cancer, glioblastoma, CRC, cholangiocarcinoma, prostate cancer, epithelial ovarian cancer, and cervical cancer.^272,297,300–303^ In summary, the expression of m^5^C and its related enzymes in tumors is closely related to the occurrence, development, and prognosis of tumors, providing new strategies for the diagnosis and treatment of tumors.

#### 7-Methylguanosine (m^7^G)

The m^7^G modification occurs in a specific subset of tRNAs, serves to stabilize these modified tRNAs and is essential for the efficient translation of mRNA.^304^ The methylation process of m^7^G modification is primarily catalyzed by methyltransferases such as METTL1 and RNMT, which are writer proteins that add m^7^G modifications to RNA molecules, including tRNA and mRNA. Reader proteins that recognize m^7^G modifications include eIF4E, QKI, and NCBP2, which are involved in regulating RNA maturation, nuclear export, and translation processes. However, no demethylase for m^7^G has been identified to date.^305^ A growing body of evidence implicates m^7^G in the development of various human diseases, particularly cancer.^306^ Disruptions in m^7^G levels are intricately linked to the onset and advancement of malignancies, as they regulate the transcription of numerous oncogenes and tumor suppressor genes.^307^ Abnormalities in m^7^G modification may lead to changes in tumor cell proliferation, migration and invasion.^308^

##### METTL1

The writer of the m^7^G modification is primarily METTL1, which forms a complex with WDR4. METTL1 and WRD4 are upregulated in diverse kinds of cancer, including esophageal, liver, colorectal, lung, and nasopharyngeal carcinomas.^309–311^ In bladder cancer cells and lung cancer cells, METTL1 notably enhances cellular proliferation, migration, and invasion.^299^ Besides, the expression of WDR4 is significantly correlated with advanced-stage prostate cancer.^312^ And elevated expression of METTL1 or WDR4 indicates a less favorable prognosis in patients with osteosarcoma.^313^ The above results highlight their potential as prognostic biomarkers and therapeutic targets in cancer.

##### Quaking proteins (QKI)

Recent research has revealed that the QKI protein, which is downregulated in a spectrum of malignant tumors such as lung, gastric, and CRC,^314–317^ and serves as a tumor suppressor in various human cancers, encompassing oral, colon, gastrointestinal, and prostate cancer^318,319^ (Fig. 3). Specifically, in NSCLC, QKI-6 has been shown to inhibit EMT processes by modulating the EGFR/SRC/STAT3 signaling pathway, thereby upregulating the expression of AGR2.^320^ QKI is suppressed in a variety of tumors, and its expression levels are strongly correlated with the aggressiveness, metastatic potential, and clinical outcomes of these tumors. In summary, functioning as intrinsic m7G-recognizing proteins within mRNA, QKIs orchestrate the regulation of target mRNA metabolism and modulate cellular chemoresistance, making them as promising candidates for therapeutic intervention.^321^

##### Nuclear cap binding protein subunit 2 (NCBP2)

NCBP2 exhibited significant upregulation in pancreatic cancer tissues compared with normal tissues. Overexpression of NCBP2 was associated with poor prognosis, especially in early-stage patients with pancreatic cancer.^322^ There is a study indicated that the expression of NCBP2 is upregulated in various types of malignant tumors, including OSCC. It has been confirmed that NCBP2 indeed suppresses the migration, invasion, and proliferation of OSCC cells.^323^ However, the specific mechanisms of NCBP2 in tumorigenesis still require further exploration.

### Non-coding RNAs

#### LncRNAs

Long non-coding RNAs (lncRNAs) represent a category of non-protein-coding transcripts exceeding 200 nucleotides in length. This extensive definition encompasses a diverse and highly variable array of transcripts that vary in their biogenesis, genomic origin, and mechanisms of action.^324^ LncRNAs play essential regulatory roles in numerous cellular processes, such as gene expression, cell differentiation, and development.^325^ They are capable of exerting an impact on DNA, RNA, and proteins to modulate gene expression by alterations in chromatin structure, transcription, and post-transcriptional processing.^326,327^

LncRNAs are increasingly recognized as pivotal regulators that are implicated in gene expression as well as a wide array of physiological and pathological processes.^328,329^ In the context of cancer, they are capable of regulating the growth, differentiation, invasiveness, and metastasis of cancer cells.^330^ Recent research has demonstrated that lncRNAs display elevated levels of expression and are frequently linked to diverse varieties of tumors. The dysregulation and mutations of these lncRNAs are significantly correlated with tumorigenesis, metastasis, and tumor progression.^331^ Furthermore, lncRNAs demonstrate specific expression patterns in certain cancer types and can be detected in circulating blood and/or urine.^327^ As such, lncRNAs represent a novel class of potential molecular indicators and therapeutic targets for oncological interventions.

Genome-wide RNA sequencing (RNA-Seq) analysis has identified numerous lncRNAs that are either upregulated or downregulated in breast cancer. LncRNAs implicated in breast cancer include HOTAIR, ANRIL, ZFAS1, HOTAIRM1, PVT1, MALAT1, and LNP1, among others.^332^ HOTAIR suppresses tumor suppressor genes like PGR, PCDH10, PCDHB5, and JAM2, promoting breast cancer development, and is overexpressed in colorectal, hepatocellular, gastrointestinal, and NSCLC.^333–335^ Besides, ANRIL is also upregulated in breast cancer. In lung cancer, lncRNAs such as MALAT1, CCAT2, HOTAIR, AK126698, HNF1A-AS1, SOX2-OT, MEG3, ANRIL, H19, CARLo-5, MVIH, PVT1, EVADR, SPRY4-IT1, GAS5, PANDAR, BANCR, and TUG1 are involved.^336,337^ MALAT1 is overexpressed in lung cancer, enhancing cell proliferation, EMT, and angiogenesis.^338^ In addition, CCAT2 is also overexpressed in NSCLC, increasing its invasiveness.^339^ MEG3 is downregulated in NSCLC, influencing immunity and autophagy via the miR-543/IDO pathway.^340^ Besides, In HCC, several lncRNAs, including MALAT1, HULC, HEIH, DILC, and HOTAIR, are upregulated, with HULC promoting HCC growth metastasis and drug resistance.^341,342^ However, lncRNA DILC has been identified as a tumor suppressor gene that can suppress the stemness of tumor cells.^343^ A vast number of lncRNAs have been identified in various other types of cancers, and more details are presented in figures and tables. Many lncRNAs are abnormally expressed in different tumors (Fig. 3), with some being cancer-specific. They are stable in body fluids and can be detected in the plasma and urine of cancer patients, reflecting the severity of the disease. These characteristics render lncRNAs promising non-invasive biomarkers and therapeutic targets for cancer treatment, although challenges and the need for validation still exist for their clinical application.

#### miRNAs

miRNAs are small non-coding RNAs with a length of approximately 22 nucleotides (nt), which are widely recognized to play a significant role in the post-transcriptional regulation of mRNA.^344^ The typical biogenesis process of miRNA involves three distinct stages: primary miRNA (pri-miRNA), precursor miRNA (pre-miRNA), and miRNA duplex formation.^345,346^ Subsequently, one strand of the miRNA duplex is integrated into the RNA-induced silencing complex (RISC), which triggers the decay of mRNA and translational suppression by interacting with the complementary sequences in the 3’-untranslated region (3’-UTR) of target gene mRNA.^347,348^ Notably, miRNA-mediated gene expression control is critical for the cellular response to the environmental stresses, like starvation, hypoxia, oxidative stress, and DNA damage.^346,349^

Undoubtedly, miRNAs stand out as the most intensively studied category of ncRNAs, especially within the context of cancer research. In 2002, the first evidence showing the role of miRNAs in human disease was reported^350^ (Fig. 1). Since then, a substantial amount of research has indicated that aberrant regulation of miRNA expression is an intricately linked to the progression of tumors.^351,352^ The underlying mechanisms encompass chromosomal aberrations (such as the amplification or deletion of miRNA genes), alterations in transcriptional regulation, epigenetic modifications, and flaws in the miRNA biogenesis machinery.^353^ Over the past two decades, the association between miRNAs and diverse types of cancers has been the subject of extensive investigation. Drawing upon the evidence regarding miRNAs, a multitude of potential cancer biomarkers for both diagnosis and prognosis have been proposed, thereby offering a novel vantage point for cancer screening.

In 2002, the deletion and low-expression of miR-15 and miR-16 cluster in chronic lymphocytic leukemia were demonstrated, which initially shed light on the role of miRNAs in the progression of cancers.^354^ Concurrently, a decrease in these two miRNAs was noted in cancerous tissues compared to normal tissues. Over the past years, miRNAs have been reported to be implicated in almost all known cancer processes. miR-21 demonstrates a potential oncogenic function and targets tumor inhibitor proteins in almost all types of cancer, including glioblastoma, head and neck cancer, ovarian cancer, B-cell lymphoma, HCC, cervical cancer, and lung cancer.^355^ Likewise, miR145 is also highly expressed in numerous malignancies and plays a profound role in cancer initiation. miR-145 is overexpressed in colon cancer, ovarian cancer and so on^356,357^ (Fig. 3). Conversely, miRNA-34 exhibits tumor-suppressive effects in various types of cancer, including gastric cancer, CRC, prostate cancer, breast cancer. miR-34 is epigenetically downregulated or silenced in colorectal cancer tissues and cell lines, miR-34 regulates several different target genes and signaling pathways, inducing apoptosis, senescence, and cell cycle arrest and repressing gastric cancer cell proliferation, migration and metastasis, thus contributing to the suppression of carcinogenesis and cancer progression.^358^

Additionally, miR-155 has been identified as being dysregulated in several types of human tumors, and it is regarded as functioning either as an oncogene or a tumor suppressor, depending on tumor system. high expression of miR-155 has been shown in B-cell lymphomas, colon cancer and lung cancer as an oncogenes,^359,360^ whereas low expression in ovarian cancer and melanoma as an oncosuppressor-miR.^360,361^ There are many miRNAs are also dysregulated during the development and progression of cancer. For example, miR-148a and miR-152 are downregulated in gastric cancer tissues^362^; miR-128 is significantly downregulated in breast and lung cancer tissues^363,364^; and miR-375 is significant overexpressed in lung cancer.^365^ The information presented in Fig. 3 and Table 1 further enriches our understanding of these miRNA-mediated cancer-related phenomena, emphasizing the need for continued research in this significant area.

#### CircRNAs

CircRNAs fall within the classification of ncRNA molecules, which were initially identified in pathogens back in the 1970s.^366^ Typically, circRNAs are generated from precursor mRNA (pre-mRNA) through the back-splicing process, also referred to as alternative splicing.^367^ CircRNA can be categorized into three types: exonic circular RNA (ecircRNA), which consists solely exons, ciRNA, which originates from intron lariat 3 and exon-intron circRNA (EI-ciRNA), which is made up of both exon and intron sequences.^368^ CircRNAs represents a single-stranded RNA that differentiates itself from linear RNA on account of its covalently closed structure. Lacking 5’ caps and 3’ poly(A) tails, this unique structural feature endows circRNAs with enhanced stability and a remarkable resistance to RNase R. This unique form grants circRNAs greater stability and resistance to RNase R. They possess the ability to bind miRNAs, functioning as sponges, and thereby partake in a diverse range of physiological functions, such as cell cycle regulation, intercellular communication, as well as transcriptional and translational regulation.^369,370^

In the past, circRNAs were regarded as by-products resulting from aberrant splicing. CircRNAs were extensively discovered in mammalian transcriptomes in 2012 (Fig. 1), which subsequently led to their attracting substantial attention in the realm of cancer research.^371,372^ They are crucial in tumorigenesis, invasion, metastasis, and chemoresistance, and may serve as novel diagnostic biomarkers and anticancer targets. Research indicates that circRNAs are differentially expressed in various tumors, including colon, ovarian, gastric, esophageal cancers, and gliomas.^373,374^ They regulate the expression of oncogenes and tumor suppressor genes through various mechanisms, including acting as miRNA sponges to adsorb miRNAs or binding to RNA-binding proteins (RBPs) to form complexes, thereby affecting gene transcription.^375^ CircRNAs have been associated with a range of physiological states and cellular attributes, such as stemness and pluripotency, and are thus potentially involved in initiating and perpetuating oncogenesis. Furthermore, circRNAs have been linked to various clinical parameters, including tumor grade, size, metastatic stage, and malignancy aggressiveness.

Recent studies have revealed that abnormal expression of circRNAs are widespread across nearly all types of cancer and play crucial roles in cancer pathogenesis (Fig. 3), functioning either as tumor suppressors or oncogenes.^373^ For example, circMTO1 is downregulated in HCC and suppress HCC progression by sponging oncogenic miR-9 to promote p21 expression.^376^ A similar ceRNA mechanism also applies to circTRIM33-12, which is also down-regulated in HCC tissues and cell lines.^377^ Conversely, circRHOT1 is significantly upregulated in HCC, which promote proliferation and metastasis.^378^ Additionally, multiple circRNAs are found to be dysregulated in gastric cancer. For the past several years, circRNAs were regarded as potential targets in the clinical treatments of gastric cancer. There were studies indicated that circCACTIN and circNHSL1 are upregulated in gastric cancer, which promoted cell migration and invasion,^379,380^ and circFGD4 is downregulated in gastric cancer.^381^ In addition, recent studies have indicated that a variety of circRNAs are involved in the progression of breast cancer, primarily by acting as miRNA sponges,^382^ circCDYL and circHSDL2 were found upregulated the breast cancer cell lines and clinical tissues,^383,384^ which indicate a potential prognostic marker for breast cancer. Besides, circRNAs also play regulatory roles in the progression and metabolism of lung cancers. Another study has identified a novel exon-derived circRNA, circSLC25A16, which has the ability to accelerate the glycolysis and proliferation of NSCLC cells.^385^ The circTP63 and cercaria were also found to be overexpressed in lung cancer.^386,387^ Dysregulation of circRNAs has also been found in other types of cancer. circLPAR3 and hsa_circRNA6448-14 are upregulated in esophageal cancer.^388,389^ CircFOXK2 and circBFAR were found to be overexpressed in pancreatic cancer (PC).^390,391^ These circRNAs have emerged as hot topics in current cancer research due to their significant roles in tumor development, as well as their potential as biomarkers and therapeutic targets. As research continues to advance, an increasing number of circRNAs will be identified to play a crucial part in the diagnosis and treatment of cancer.

#### piRNAs

piRNAs constitute a distinct class of small silencing RNAs in the animal kingdom, distinguishing themselves from miRNAs and siRNAs. They possess 2ʹ-O-methyl-modified 3ʹ ends and form complexes with PIWI proteins, in contrast to miRNAs and siRNAs which pair with AGO proteins.^392–394^ The PIWI/piRNA complex is known for regulating transposon silencing and reproductive development by controlling gene expression at the transcriptional or post-transcriptional level.^395,396^ PIWI protein/piRNA deletion or disruption can reactivate transposons, potentially leading to germ cell tumors. In contrast, PIWI proteins are highly expressed in various other tumors, including gastric, colon, liver, glioma, and bladder cancers.^397–400^ piRNAs regulate gene expression in cancer analogous to normal cells, dependent on PIWI proteins, and can also exert epigenetic control by interacting with regulatory factors or through direct regulating their expression.^401^ Additionally, piRNA can also bind to target genes, thereby degrading mRNA, altering its stability, or inhibition of its translation.^402,403^

Around 2010, it was initially reported that piRNAs exhibited abnormal expressions in cancer (e.g. upregulation of piR-651 in several cancer cell lines^404^). Current studies indicated that piRNA and PIWI are significantly abnormally expressed in gastric, breast, kidney, colon, and lung cancers, and are involved in the initiation, progression, metastasis and therapy resistance of cancers, which may be the potential diagnostic tools, prognostic markers, and therapeutic targets for cancers.^402^ A study has analyzed the expression patterns of piRNAs in tumor tissues by means of whole-transcriptome piRNA sequencing or PIWI-interacting/bound RNA sequencing, which has unveiled an abnormal expression profile of piRNAs/PIWI between tumor tissues and normal tissues.^405^

Among these piRNAs, abnormal expression of pir-651 and pir-823 is the most widely associated with various cancer types.^406^ It has been proved that in gastric cancer, the expression of piR-651 is significantly higher than in adjacent normal tissue,^404^ while the expression of piR-823 is markedly reduced.^407^ Concurrently, the expression of PIWIL1 is upregulated in gastric cancer cells. Moreover, these aberrant changes are associated with the progression of gastric cancer and poor prognosis. Similarly, it has been reported that piR-651 was overexpressed in breast cancer,^408^ and it has the ability to boost the proliferation and migration, while suppressing apoptosis of breast cancer cells by facilitating DNMT1-mediated PTEN promoter methylation.^408^ In addition, other piRNAs such as piR-4987, piR-20365, piR-20485, piR-20582,^409^ piR-021285^410^ and piR-932^411^ are highly expressed in breast cancer, while the expression of piR-36712 is significantly lower in breast cancer patients compared to normal tissue.^412^ Notably, piR-021285 mediates the hypermethylation of AATF, ARHGAP11A, PIP4K2B, THAP10, and other related oncogenes in breast cancer tissue, thereby promoting the progression of breast cancer, highlighting the importance of crosstalk between various epigenetic modifications.^410^ In various types of tumors, the abnormal expression of piRNAs has been widely reported, such as piR-823, piR-54265, piR-18849, and piR-19521 in colorectal cancer,^413,414^ and piR-651 and piR-55490 in lung cancer.^415,416^ For more details, please refer to (Fig. 3) and the Table 1. It is worth highlighting that circulating piRNAs in the blood of tumor patients are regarded as potential cancer biomarkers for detection and prognosis. Furthermore, machine learning-based diagnostic methods for CRC utilizing piRNAs have already been developed.^417^ As we further investigate the intricate regulatory mechanisms and functions of piRNAs in the context of cancer, it is expected that they will offer more valuable insights and possibilities for improving cancer diagnosis, prognosis, and treatment strategies in the future, highlighting the importance of further in-depth investigations in this promising area.

## Mechanisms of cancer therapy resistance by epigenetic networks

Cancer therapy resistance (or therapeutic resistance) pertains to the diminished sensitivity of tumor cells towards various treatment modalities, including chemotherapy, radiotherapy, targeted drugs, and immunotherapy.^418^ This resistance is one of the main reasons for treatment failure in cancer patients, leading to diminished responsiveness, rapid disease progression, relapse, metastasis, and ultimately, patient mortality.^419^ Therapy resistance can be roughly categorized into intrinsic and acquired resistance.^2^ The intrinsic resistance is mediated by the inherent or endogenous characteristics that are exist in tumor cells or tissues prior to initial treatment, such as effective DNA damage repair mechanisms, tumor growth kinetics and stem cell like properties, that offer cancer cells survival benefits and ability to adapt to primary therapeutic stress.^420,421^ This can manifest as an unresponsive initial reaction to therapies, even at elevated doses. Differently, acquired resistance refers to the development of resistance to cancer therapies after an initial response, mainly based on factors such as genomic instability (mutations), tumor cells heterogeneity, cellular plasticity, and adaptive responses, which can manifest as local tumor recurrence or distal metastases following clinical remission.^422^

**Fig. 4:**
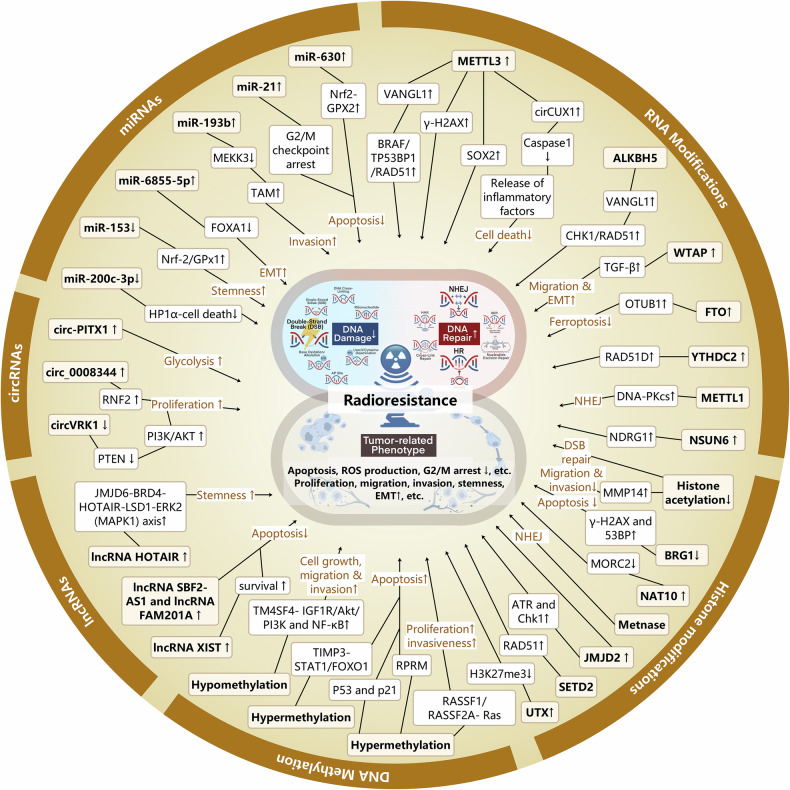
Mechanisms of epigenetics impacting on radiotherapy resistance. It delineates the mechanisms by which epigenetic factors contribute to radiotherapy resistance, primarily through the modulation of DNA damage repair processes and tumor-related phenotypes. DNA damage repair pathways play a role in radiotherapy resistance. Among them the DNA double-strand break repair pathways, specifically homologous recombination repair and nonhomologous end joining, are crucial in conferring radioresistance. Furthermore, the activation of these DNA damage repair pathways enhances resistance to radiotherapy. Besides, tumor-associated phenotypes, including cell cycle regulation, apoptosis, autophagy, EMT, and cell proliferation, may serve as potential targets for enhancing radiosensitivity through epigenetic modifications. HR homologous recombination repair, NHEJ nonhomologous end joining, BER base excision repair, MMR mismatch repair, EMT epithelial-mesenchymal transition

**Fig. 5:**
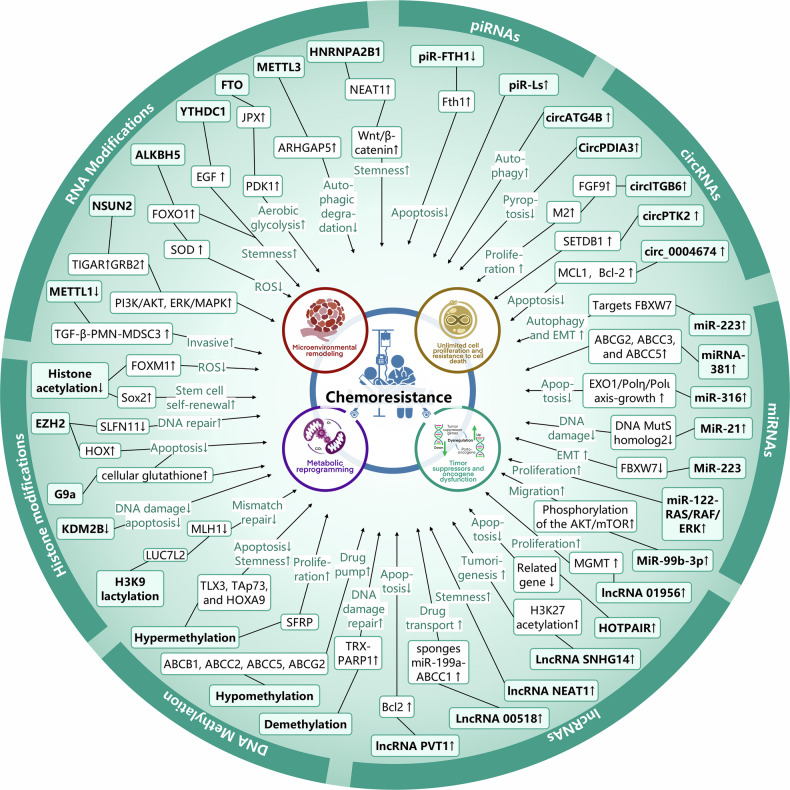
Mechanisms of epigenetics impacting on chemotherapy resistance. It depicts the pathways of epigenetic regulation of chemotherapy resistance, which can be summarized into the following four aspects: dysregulation of tumor suppressor gene and oncogene, metabolic reprogramming, unlimited cell proliferation and resistance to cell death, and microenvironment remodeling

**Fig. 6:**
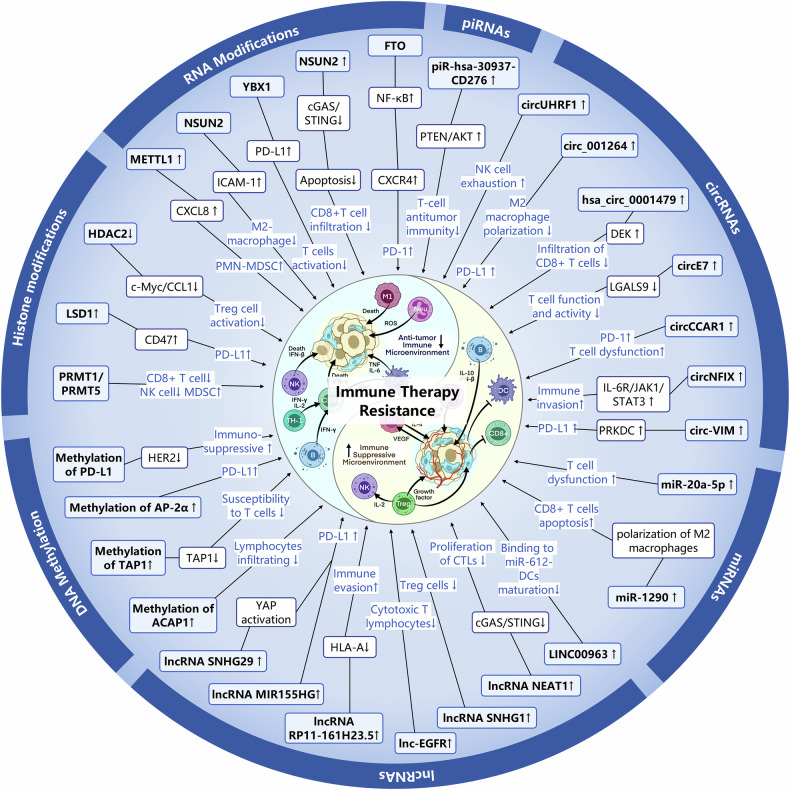
Mechanisms of epigenetics impacting on immunotherapy resistance. It illustrates the mechanisms by which epigenetic modifications influence resistance to immunotherapy. Epigenetic processes predominantly modulate the activity of immune cells and the expression of the immune checkpoint proteins, specifically, PD-1 and PD-L1. Particularly, the activity of CD8^+^ T cells, Tregs, and MDSCs, along with the expression of PD-1, are principal targets for epigenetic regulation in the context of immunotherapy resistance. Tregs regulatory T cells, MDSCs myeloid-derived suppressor cells, NK nature killer cell, DC dendritic cell, M1 M1 macrophages, M2 M2 macrophages

**Fig. 7:**
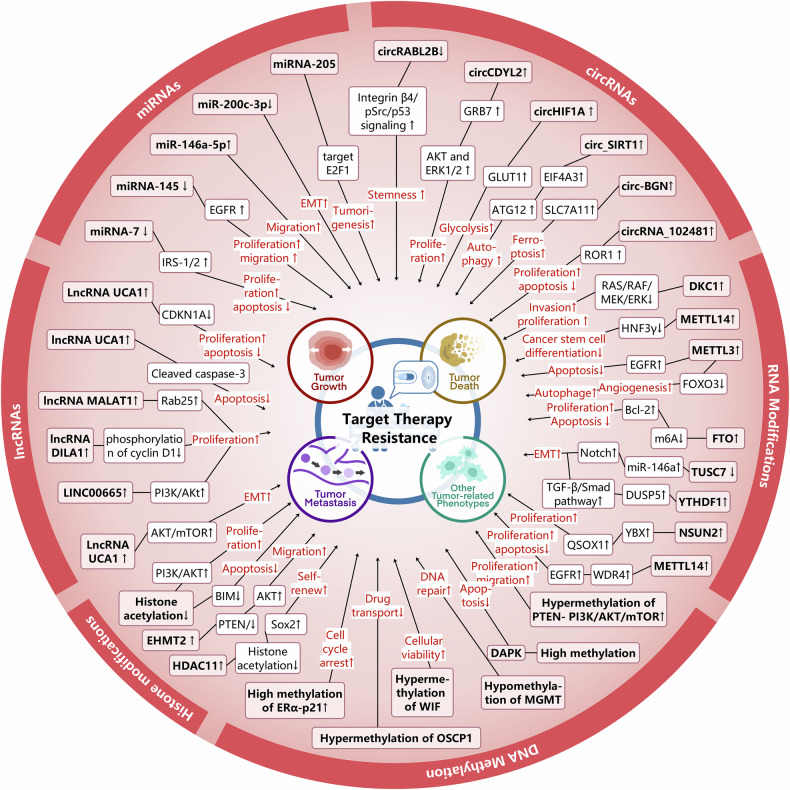
Mechanisms of epigenetics impacting on targeted therapy resistance. It delineates the mechanisms by which epigenetic factors contribute to targeted resistance. The factors could be summarized into the following four aspects: tumor growth, tumor metastasis, tumor death and other tumor-related phenotypes including glycolysis, cancer stemness and drug transport

In recent years, the mechanisms by which cancer cells develop resistance to various treatment methods have been gradually revealed, and it has been comprehensively summarized in several reviews.^418,423,424^ Additionally, various forms of epigenetic regulations have been successively verified to be associated with cancer treatment resistance (Figs. 4–7). In general, the decreased responsiveness of cancer cells are related to diverse mechanisms, which typically entail the interactions of genetic factors, nongenetic elements and microenvironment.^423^ It is widely acknowledged that genetic factors play a predominant role in the development of therapeutic resistance in cancer cells.^2^ One of the hallmark characteristics of cancer cells is genomic instability, which means that as cancer cells proliferate, various mutations will accumulate in genome, thereby inducing a range of phenotypes to swiftly adjust their transcriptional and/or metabolic program to cope with and endure the therapeutic pressure.^425^ However, accumulative evidence also supports those nongenetic factors, including epigenetic regulation, also play a significant role in the therapeutic resistance of cancer. The notion that a single cancer genome is capable of producing a multitude of phenotypic states, and cancer cells can shift between these states without fundamental genomic alterations is gaining greater acknowledgement.^426^

When cancer cells are subjected to different therapeutic pressures such as chemotherapeutic drugs, targeted agents, ionizing radiation, and monoclonal antibodies, they can gradually evolve different mechanisms of resistance.^3,427,428^ Due to the diverse mechanisms of action of various chemotherapeutic drugs, the mechanisms of chemotherapeutic resistance in cancer are quite complex. Tumor cells primarily achieve resistance to traditional chemotherapy drugs through mechanisms such as alteration of drug metabolism, reduction of drug absorption, increased drug pumping out, efficient DNA damage repair, resistance to cell death, EMT, cancer stem cells, and the tumor microenvironment.^429^ In addition to the aforementioned factors, tumor cells can gradually acquire resistance to targeted drugs through heterogeneity, plasticity, and adaptability.^427^ Unlike chemotherapy, the mechanism of radiotherapy is relatively straightforward, mainly relying on the destruction of biomolecules such as DNA by ionizing radiation. Therefore, tumor cells can mitigate the damage of ionizing radiation through chromatin remodeling, and achieve resistance to radiotherapy by relying on efficient DNA damage repair and growth dynamics (cell cycle regulation, cell death resistance and metabolic reprogramming).^420^ The resistance of tumor cells to immunotherapy mainly arises from the tumor’s regaining of immune evasion capabilities through various mechanisms, coupled with the suppression of immune cells within the microenvironment.^430^ The factors mentioned above are all intricately regulated by epigenetic modifications. In this context, we will focus on the contribution of epigenetic modifications to tumor treatment resistance.

### Radiosensitivity

Radiotherapy is a pivotal modality in cancer treatment.^431^ However, the resistance exhibited by numerous tumor cells significantly diminishes its therapeutic efficacy.^432,433^ It is well established that radiotherapy exerts a substantial cytotoxic effect on proliferating cells. Nonetheless, tumor cells that endure radiation exposure concurrently activate a variety of pro-survival signaling pathways, such as ATM, ATR, AKT, ERK and NF-κB-mediated DNA damage checkpoints, DNA damage repair, inhibition of apoptosis, and cancer stem cell-related stem-like pathways. The configuration of compact chromatin enhances the protection of tumor cells against radiotherapy. Notably, radioresistant pathways are subject to epigenetic regulation.^434–436^ In recent years, epigenetic mechanisms in the cancer cells radioresistance have received increasing attention. Moreover, the integration of radiotherapy with epigenetic pharmacological agents has been implemented in clinical treatment.^434,437^

#### Histone modifications in radiosensitivity

Histone modifications have a vital function in controlling gene expression and chromatin structure, which modulate DNA damage response and repair pathways that are crucial to radioresistance. Currently, evidence shows that histone acetylation is closely related to radiotherapy resistance.^438^ The level of histone acetylation can affect DNA damage repair and thereby regulate radioresistance. For example, treatment with the HDAC inhibitor PCI-24781 leads to increased acetylation levels, which reduce the accuracy of DSB damage repair in SiHa cervical cancer and WiDr colon cancer cells, thereby reducing radiotherapy resistance.^439^ Conversely, decreased acetylation levels promote radiotherapy resistance. In addition to affecting DNA damage repair, histone acetylation levels also influence tumor angiogenesis and migration processes. HDAC inhibitors can suppress the expression of MMP14 in GBM, and the inhibition of MMP14 helps to reduce tumor angiogenesis, inflammation, cancer cell invasion, and metastasis, thereby promoting tumor radiotherapy resistance.^440,441^ Furthermore, histone acetylation affects DNA damage repair in cancer cells by regulating chromatin remodeling. Targeting BRG1 chromatin remodeling enzymes may enhance the radiosensitivity of cancer cells. BRG1-BRD increases the sensitivity of cancer cells to radiotherapy by disrupting the γ-H2AX and 53BP1 pathways, leading to reduced DNA repair efficiency, G2-M checkpoint defects, and increased apoptosis.^442^ Additionally, acetylation of the chromatin remodeling protein MORC family CW-type zinc finger 2 (MORC2) can activate the G2 checkpoint and induce DNA damage, thereby improving the radiosensitivity of breast cancer cells^443^ (Fig. 4).

Contrarily to histone acetylation, increased level of histone methylation leads to chromatin condensation, resulting in gene silencing.^444^ Specifically, the methylation of H3K36 and H3K27me3 may participate in the cellular response to radiation through regulation of the DNA repair pathways of homologous recombination (HR) and nonhomologous end joining (NHEJ).^445–447^ For instance, the trimethylation of H3K36 mediated by SETD2 is crucial for the activation of the ATM kinase and the recruitment of 53BP1 to DNA double-strand breaks (DSBs). Furthermore, overexpression of JMJD2A leads to a decrease in H3K36 methylation, reducing HR repair efficiency, which may weaken the radiation resistance of tumors.^448,449^ On the other hand, Metnase, a methyltransferase for H3K36, can promote NHEJ repair, thereby enhancing the radiation resistance of tumor cells.^450^ In addition, the dynamics of H3K27me3 are also related to radiotherapy sensitivity. Studies have shown that the H3K27 demethylase inhibitor GSKJ4 can disrupt radiation-induced DSB repair and reduce the radiation resistance of tumor cells. Meanwhile, the histone demethylase UTX has been found to enhance radiotherapy resistance^444^ (Fig. 4). Overall, histone methylation contributes significantly to modulate the repair of DNA damage and DSBs, thereby affecting the radiation resistance of cancer cells.

#### DNA methylation in radiosensitivity

DNA methylation exerts a substantial influence on the radioresistance of cancer cells. Researches have revealed that the DNA methylation status within tumors is intimately linked to radiosensitivity, and aberrant alterations in DNA methylation can impact the efficacy of tumors to radiotherapy. Precisely, the hypomethylation state of certain genes may facilitate the radioresistance of tumor cells by inducing specific signaling pathways. For example, in CRC, the hypomethylation of the DSTN gene potentiates the resistance for cancer to radiotherapy though activating the Wnt/β-Catenin signaling pathway.^451^ Moreover, DNA methylation can also affect the radiosensitivity of tumor cells by influencing genes related to DNA damage repair. For genes that promote DNA damage repair (e.g., *ATM/ERCC1*), DNA methylation can increase the sensitivity of tumors to radiation, while demethylation leads to radiotherapy resistance in tumor cells^452,453^ (Fig. 4). For genes that inhibit DNA repair (e.g., *RASSF1A*), DNA methylation results in radiotherapy resistance in tumor cells.^454^ DNA methylation profile of aggressiveness‐associated genes modulates the radiosensitivity in distinct manners. The elevated expression of tumor proliferation-suppressing genes serves to restrain the proliferation of tumor cells and consequently trigger radiosensitivity in the context of radiotherapy. The SERPINB5 and HIC1 genes were observed to be hypermethylated within radioresistant cells,^455,456^ whereas the TM4SF4 and miR24 genes manifested hypomethylation in these same radioresistant cells.^457^ DNA methylation can also affect cell cycle-related genes, thereby altering radiotherapy sensitivity. Radiosensitivity varies across different phases of the cell division cycle. It is worth noting that the radiosensitivity varies across different cell division cycles. Cells in the S phase exhibit resistance to irradiation, while those in the M and G2 phases display sensitivity to it. When radiotherapy is applied, cells in the sensitive phases like M or G2 are selectively eradicated.^458^ Therefore, hypermethylation of genes that promote cell entry into the G2/M phase can lead to radioresistance, such as hypermethylation of promoter site of the RPRM, which promotes radioresistance in nasopharyngeal carcinoma (NPC) cells.^459^ CCND2 is hypermethylated in radioresistant cell lines of HNSCC, and hypomethylated in radiosensitive cells.^460^ Besides, in nasopharyngeal carcinoma, hypermethylation of p53 and p21 can inhibit cell cycle arrest and reduce apoptosis, thereby leading to radiotherapy resistance.^461^ Furthermore, certain genes may also impact changes in related pathways, leading to radiotherapy resistance. The methylation-induced silencing of the tumor suppressor genes *RASSF1* and *RASSF2A*, coupled with the subsequent activation of the Ras/PI3K/Akt signaling axis, constitutes the underlying mechanism of radioresistance in individuals with OSCC.^461^ Hypomethylation of the TM4SF4 upregulates its expression and the secretion of IGF, thereby activating the IGF1R/Akt/PI3K and NF-κB signaling cascades to drive cell proliferation, migration, and invasion in lung adenocarcinoma cells exhibiting intrinsic radioresistance.^462^ Besides, hypermethylation of TIMP3 activates the STAT1/FOXO1 pathway, leading to radiotherapy resistance^463^ (Fig. 4). In summary, DNA methylation wields a profound and intricate influence on the radioresistance of cancer cells through multiple dimensions, such as impinging on signaling pathways, DNA damage repair genes, cell proliferation and cycle-related genes, as well as related pathways. A profound comprehension of these mechanisms holds substantial promise for devising more targeted strategies to surmount radioresistance and enhance the therapeutic effectiveness of radiotherapy in oncological interventions.

#### RNA modifications in radiosensitivity

During radiotherapy, RNA modifications play a crucial role in regulating DNA damage repair, cancer stemness, apoptosis and G2/M arrest, all of which are relevant to radiosensitivity of cancer cells.^464,465^ Ionizing radiation can alter the levels of RNA modifications and the activity of related enzymes, which in turn may modulate the sensitivity of cancer cells to radiation.^466,467^

Research has demonstrated that the m^6^A methyltransferase METTL3 serves as a critical element in enhancing radioresistance by promoting DNA damage repair and inhibiting cell death pathways. In the context of GBM, exposure to IR has been shown to upregulate METTL3 expression. Notably, overexpressed METTL3 interacts with SOX2 transcripts, stabilizing them and thereby contributing to heightened DNA damage repair capabilities, which in turn bolsters the resistance of glioma stem-like cells to γ-irradiation.^468^ Additionally, METTL3-mediated m^6^A modification of mRNA impedes the degradation of H2A histone family member X (H2AX) mRNA, leading to increased H2AX expression and facilitating both DNA damage repair and cell survival, and then promote radiation resistance.^469^ In pharyngeal squamous cell carcinoma, METTL3 has been identified as a key inducer of radioresistance. It achieves this by enhancing the stability of circCUX1, which in turn binds to caspase1 mRNA and suppresses its expression. This inhibition of caspase1 leads to a decrease in programmed cell death, thereby conferring radioresistance.^469,470^ Additionally, in lung adenocarcinoma, METTL3 expression is upregulated, contributing to the stabilization of VANGL1. This upregulation activates the BRAF/TP53BP1/RAD51 pathway, reducing DNA damage and fostering radioresistance.^471^ Conversely, METTL3 also enhances radioresistance by modulating the invasiveness and migratory capabilities of cancer cells. In gastric cancer, the upregulation of WTAP, a subunit of the METTL3 complex, boosts TGF-β expression. This increase in TGF-β promotes EMT and the migration of gastric cancer cells, ultimately leading to radioresistance in gastric cancer^472^ (Fig. 4).

RNA demethylases also function as a central component in radiation sensitivity. Recent studies have shed light on the distinct roles of m^6^A demethylase ALKBH5 in radioresistance compared to METTL3. Contrary to METTL3, ALKBH5 does not have an opposing effect; instead, it enhances radiotherapy resistance in GBM stem cells by modulating HR.^469,473^ Furthermore, the transcription factor FOXM1 has been implicated in DNA damage repair. The upregulation of FOXM1 induced by IR is mitigated by ALKBH5 inhibition, suggesting an alternative pathway through which ALKBH5 may mediate radioresistance in GBM.^474–476^ In addition, the m^6^A demethylase FTO has been implicated in the development of radioresistance. This enzyme is found to be upregulated in radioresistant NPC, where it enhances the radioresistance of the cancer cells,^477^ This enhancement is achieved by promoting the activity of the deubiquitinase OTUB1, which in turn mediates anti-ferroptosis, a process that stabilizes the expression of SLC7A11 and is crucial in human cancer.^478^ Beyond its role in DNA DSB repair, excision repair also contributes to radioresistance. FTO is capable of demethylating β-catenin mRNA in cervical squamous cell carcinoma, thereby stabilizing β-catenin expression and activating the excision repair gene, ERCC1, which is a key factor in conferring resistance to radiotherapy.^479^ The biological functions of m^6^A modification extend beyond its “writing” (writers) and “erasing” (erasers) processes; its “reading” (readers) proteins also play a crucial role in regulating radiation sensitivity. YTHDF3, as an m^6^A reader, can recognize m^6^A modifications and promote the m^6^A-dependent translation of the DNA repair protein RAD51 paralog 4 (RAD51D), thereby affecting the role of hepatocyte nuclear factor 1-α (HNF1α) in promoting radioresistance in cervical cancer cells^480^ (Fig. 4). This discovery reveals the significant role of m^6^A in the regulation of radiation therapy sensitivity, providing new molecular mechanisms and promising anticancer targets of radiotherapy.

Beyond the realm of m^6^A, both N4-methylcytosine (m^5^C) and N7-methylguanosine (m^7^G) have been implicated in their association with radiosensitivity. The expression of the m^5^C writer NSUN6 has been found to be clinically correlated with radioresistance and unfavorable prognosis in cervical cancer.^481^ NSUN6 facilitates the m^5^C modification of NDRG1 mRNA, thereby enhancing its stability and activating the HR pathway, which in turn augments the radioresistance of cervical cancer.^481^ On the other hand, the m^7^G writer METTL1 is crucial for nonhomologous end-joining repair and plays a role in the radioresistance of HCC by modulating the DNA-dependent protein kinase catalytic subunit or DNA ligase IV.^482^ Additionally, METTL1 expression in tumor tissue is significantly associated with a poor prognosis for HCC patients undergoing radiotherapy. In the context of HCC, gene networks related to m^7^G and radiotherapy resistance, as well as prognostic models, have been established.^483^ In summary, RNA modifications play a crucial role in the occurrence, development, and the formation of radiotherapy resistance in cancer (Fig. 4). By precisely regulating RNA modifications, we can reduce the resistance of tumors to radiotherapy and thereby enhance the effectiveness of cancer treatment. This regulatory strategy not only targets the biological characteristics of cancer but also may offer new perspectives and approaches for cancer therapy.

#### Non-coding RNAs in radiosensitivity

##### LncRNAs

The role of lncRNAs in cancer drug resistance has now been firmly established.^484^ Emerging evidence suggests the involvement of lncRNAs in radiation therapy response. LncRNAs alter the radiosensitivity of cancer cells by regulating radiation-related pathways, including DNA damage repair, cell cycle, cancer stem cell phenotype, and apoptosis.^485^ For example, the downregulation of the lncRNA XIST expression inhibits the viability and survival of NSCLC cells, facilitates apoptosis, and enhances sensitivity to ionizing radiation. Conversely, XIST promotes the radioresistance of NSCLC cells by modulating the expression of miR-16-5p and WEE1. Therefore, it may serves as a novel target for NSCLC radiotherapy,^486,487^ Additionally, lncRNA SBF2-AS1 and lncRNA FAM201A also promote radiotherapy resistance in NSCLC by reducing cellular apoptosis^488^ (Fig. 4).

Furthermore, lncRNAs are capable of regulating the stemness of cancer cells, thereby impacting radiosensitivity. LncRNA HOTAIR can uphold the stemness of liver cancer stem cells (LCSCs) and further intensify their radioresistance through the JMJD6-BRD4-HOTAIR-LSD1-ERK2 (MAPK1) axis.^489^ LncRNA HOTAIR also exerts a pivotal influence in DNA damage repair by promoting recruitment of EZH2 to the MYC promoter and upregulating the transcription of DDR molecules such as KU70, KU80, DNA-PKs, and ATM, which increases DNA repair in breast cancer and leads to radioresistance.^490^ In addition to targeting ATM, lncRNA HOTAIR can also interact with ATR to reduce DNA damage and promote radiotherapy resistance in CRC^334^ (Fig. 4).

LncRNAs modulate intracellular autophagy levels, thereby affecting the radiosensitivity of cancer cells. There was a study indicated that LINC-RA1 maintained the stability of H2B K120 monoubiquitination (H2BK120ub1) by interacting with H2B and suppressing the engagement between H2Bub1 and ubiquitin-specific protease 44 (USP44), thereby suppressing autophagy and consequently fostering radioresistance in glioma.^491^ Another crucial function of lncRNAs is to act as a sponge for miRNAs, exerting pivotal functions in radioresistance through the modulation of genes or proteins at both the transcriptional and post-translational levels. LncRNA SP100-AS1 induces radioresistance in CRC through sponging miR-622 and enhancing the stability of ATG3.^492^ LncRNA FGD5-AS1 promotes the radiotherapy resistance in breast cancer cells by upregulating MACC1 expression through competitive binding with miR-497-5p.^493^ Besides, lncRNA also induce radioresistance via activating multiple signaling pathway, including PI3K/AKT/mTOR^494^ Wnt /β-Catenin^495^ YAP1/AKT.^496^

In summary, the mechanisms through which lncRNAs contribute to radiotherapy resistance are complex and diverse, presenting potential targets for adjunctive radiotherapy treatments in the future. Continued exploration of these molecular mechanisms is indispensable for devising novel strategies to enhance the efficacy of radiotherapy in cancer patients.

##### miRNAs

miRNAs play an intricate and pivotal role in tumor radiotherapy resistance. They exert an impact on the sensitivity of tumor cells towards radiotherapy by regulating a diverse array of molecular mechanisms and signaling pathways. Specifically, miRNAs modulate the DNA damage repair process, either promoting or inhibiting radiotherapy resistance. For instance, miR-200c-3p is downregulated in radiotherapy-resistant prostate cancer cells, where it promotes DNA damage repair by targeting HP1α, reducing cell death, and thus contributing to radiotherapy resistance.^497^ Additionally, miRNAs are involved in regulating the activation of cell cycle checkpoints, autophagy processes, and apoptosis, thereby affecting the efficacy of radiotherapy. miR-450a-5p inhibits autophagy to enhance the sensitivity of esophageal squamous cell carcinoma to radiotherapy.^498^ In head and neck cancer, miR-630 induces anti-apoptotic effects through the Nrf2-GPX2 molecular axis, enhancing radiotherapy resistance.^499^ miRNAs can also foster radiotherapy resistance by targeting specific signaling pathways, such as miR-193b-3p in nasopharyngeal carcinoma, which accelerates tumor-associated macrophages (TAM) activation by directly repressing mitogen-activated protein/ERK kinase kinase 3 (MEKK3), promoting the invasion and radiotherapy resistance of nasopharyngeal cancer cells.^500^ In breast cancer, miR-21 causes G2/M phase arrest and reduces apoptosis in cancer cells, which is related to radiotherapy resistance.^501^ Moreover, miRNAs also induce EMT in tumor cells, such as miR-6855-5p in pancreatic cancer, which promotes radiotherapy resistance by inducing EMT through the suppression of FOXA1.^502^ It is widely acknowledged that the level of reactive oxygen species (ROS) constitutes an important factor in radiosensitivity. Recent studies have demonstrated that miRNA could modulate ROS production.^503^ In glioma stem cells, the downregulation of miR-153 leads to the upregulation of its putative target Nrf2, resulting in the accumulation of glutathione peroxidase 1 (GPx1). This antioxidant enzyme increases radiation resistance by downregulating ROS levels.^504^ miRNAs exhibit a multifaceted and intricate role in the development of resistance to radiotherapy (Fig. 4). A comprehensive investigation into the mechanisms through which miRNAs influence radiotherapy resistance is crucial for the advancing novel therapeutic strategies and enhancing radiotherapy efficacy.

##### CircRNAs

CircRNAs play a role in the regulation of cancer resistance of radiotherapy via diverse mechanisms, among which functioning as miRNA sponges to modulate radiosensitivity is a prominent aspect. In gliomas, circ_0008344 can function as a molecular for miR-433-3p and promote the transcription of RNF2, thereby promoting radiotherapy resistance.^505^ In esophageal squamous cell carcinoma, circVRK1 positively regulates PTEN by operating as a molecular sink for miR-624-3p, and the upregulated PTEN suppresses the functionality of the PI3K/AKT signaling cascade, leading to increased radiotherapy sensitivity in esophageal squamous cell carcinoma.^506^ Additionally, circRNAs affect tumor radioresistance by influencing glycolysis. For example, circ-PITX1 promotes glycolysis in gliomas and induces radiotherapy resistance.^507^ CircRNAs also affect tumor radiotherapy resistance by influencing apoptosis, autophagy and EMT. In NSCLC, upregulated circ-0086720 inhibits cell survival and reduces apoptosis, thereby increasing its radiotherapy resistance.^508^ In CRC, circ-ZNF609 promotes advancement and radioresistance of prostatic carcinoma cells by accelerating the glycolytic action of the miR-501-3p/HK2 axis.^509^ In CRC, the upregulation of circ-0055625 promotes cell migration, proliferation, migration, and further induces radiotherapy resistance in CRC.^510^ CircRNAs also induce radiotherapy resistance by binding to related molecules. In hypopharyngeal squamous cell carcinoma (HPSCC), circCUX1 binds to caspase1 and inhibits its expression, leading to reduced release of inflammatory factors and the development of resistance to radiotherapy.^470^ During cell development, different cell cycles exhibit varying sensitivities to radiation, Specifically, the G2/M phase exhibits the highest sensitivity to radiation, whereas the S phase demonstrates radioresistance. CircRNAs can also regulate cell cycles and thereby influence radiotherapy sensitivity. In nasopharyngeal carcinoma, low expression of circFIP1L1 leads to an increased proportion of the S phase during cell development, resulting in radiotherapy resistance in nasopharyngeal carcinoma.^511^ Currently, research on the role of circRNAs in tumor radiotherapy remains in its nascent stage, and numerous aspects of their regulatory mechanisms remain obscure. It is evident that the majority of circRNAs act as miRNA sponges to activate or inhibit signaling pathways. We believe that further research could place greater emphasis on other molecular mechanisms, such as post-transcriptional modifications and translation mechanisms (Fig. 4). A thorough understanding of the molecular mechanisms of circRNAs will aid in identifying novel and effective diagnostic and therapeutic targets.

### Chemoresistance

Chemotherapy is one of the cornerstones of cancer treatment, however, its effectiveness is frequently undermined by the phenomenon of chemoresistance, which diminishes the therapeutic outcomes for cancer patients.^512^ Chemoresistance is primarily characterized by the survival and sustained proliferation of tumor cells following multiple rounds of chemotherapy drugs, leading to tumor recurrence and metastasis, ultimately affecting patients’ survival and quality of life. Frequently employed chemotherapy agents encompass alkylating agents (cyclophosphamide), antimetabolites (5-fluorouracil and cytarabine), DNA crosslinking agents (cisplatin and carboplatin), anthracycline antibiotics (including doxorubicin, idarubicin, and mitoxantrone), antimicrotubular agents (paclitaxel and docetaxel), topoisomerase inhibitors (etoposide), nucleoside analogs (gemcitabine), DNA methyltransferase inhibitors (5-azacytidine), and proteasome inhibitors (bortezomib, melphalan, and carfilzomib).^513^ The development of chemoresistance is not due to a single factor but involves the complex and interwoven effects of multiple factors, including oncogene activation, impaired DNA repair function, hypoxia in the tumor microenvironment, and changes in cellular metabolism.^514^ Epigenetic modifications are notably significant in the context of chemoresistance. These modifications operate through distinct mechanisms, allowing for the control of gene expression and cellular phenotype without altering the fundamental DNA sequence, thus influencing the effectiveness of chemotherapeutic agents. For instance, epigenetic alterations such as DNA methylation and histone modification can lead to the silencing of tumor suppressor genes, thereby facilitating the development of chemoresistance.^515^ Furthermore, during chemotherapy, epigenetic reprogramming may transform the originally transient transcriptional state into a stable drug-resistant state.^516^ In the ensuing discussion, we will elaborate on the specific impacts of the epigenetic modifications on chemoresistance.

#### Histone modifications in chemoresistance

Recently, a multitude of researches underscored the significant role of histone PTMs in shaping chemoresistance to cancer therapy, influencing processes such as apoptosis, EMT, DDR, and the intricate dynamics of cancer stem cell behavior.^517,518^

In the realm of histone acetylation, HDACs have emerged as pivotal factors in chemotherapy resistance. HDACs mitigate cancer chemotherapy resistance by inhibiting the proliferation of cancer stem cells though regulating oncogene promoters. Domatinostat (a histone deacetylase inhibitors (HDACi)) have been shown to sensitize pancreatic cancer to gemcitabine/taxol by targeting the cancer stem cell compartment through the modulation of FOXM1.^519^ Additionally, inhibitors of HDAC11 are found to attenuate the self-renewal capacity of lung adenocarcinoma stem cells and overcome resistance to chemotherapy agents by downregulating Sox2, thereby offering novel pathways for enhancing the efficacy of cancer treatments^520^ (Fig. 5).

The correlation between histone methylation and resistance to cancer chemotherapy has been thoroughly investigated, revealing its influence on chemotherapy resistance through the modulation of DSBs repair and the induction of apoptosis. For instance, the overexpression of EZH2, a histone methyltransferase, has been shown to stimulate chemoresistance in glioblastoma, small-cell lung cancer, and HNSCC.^521,522^ Regarding the underlying mechanisms, EZH2-mediated gene silencing of SLFN11, a crucial factor in DNA damage repair, via H3K27 hypermethylation, leads to chemoresistance in small-cell lung cancer cells.^521^ Additionally, histone methylation alterations modulate tumor cell apoptosis and proliferation to influence chemotherapy resistance. Reports indicate that targeting EZH2 can modulate the H3K27me3 level of HMGA2 to inhibit PI3K/Akt phosphorylation, thereby suppressing cancer cell proliferation and reducing the resistance of CRC to oxaliplatin.^523^ In addition, some histone demethylases are also implicated in the development of radiation resistance. The level of G9a is positively correlated with cisplatin resistance, as it inhibits apoptosis by modulating glutathione levels, thereby increasing cisplatin resistance.^524^ Furthermore, KDM2B and GSK can enhance the chemoresistance of glioblastoma by promoting DNA damage repair.^525^ The upregulation of KDM6 can also increase the chemoresistance of lymphoma by upregulating the expression of BCL-6, which reduces apoptosis.^526,527^ Similarly, a Jumonji inhibitor, JIB-04, may serve as a potential therapeutic agent for chemo-resistant NSCLC refractory to taxane and platinum-based chemotherapy, through upregulation of pro-apoptotic genes^528^ (Fig. 5).

In addition to histone acetylation and methylation, other histone modifications also affect chemoresistance. It has been reported that reduced levels of histone H2AK119ub1 in ovarian cancer inhibit apoptosis, leading to chemoresistance.^529^ Deubiquitinating enzymes also affect the sensitivity of cancer cells to chemotherapeutic drugs through various mechanisms. For instance, USP7 contributes to chemoresistance in cervical cancer by maintaining the stability of the Chk1 protein and reducing DNA damage responses.^530^ Additionally, USP22 induces resistance to cisplatin in lung adenocarcinoma by regulating DNA damage repair mediated by γH2AX and apoptosis mediated by Ku70/Bax.^531^ Histone lactylation has also been found to affect chemoresistance. H3K9 lactylation activates LUC7L2 transcription, which reduces the expression of MutL homolog 1 (MLH1), thereby inhibiting mismatch repair (MMR), and ultimately leading to resistance to temozolomide (TMZ) in GBM^532^ (Fig. 5). Overall, histone modifications are closely related to chemoresistance, which has been well demonstrated in preclinical studies. Furthermore, the combination of histone modification inhibitors with traditional chemotherapeutic drugs has been used to improve therapeutic outcomes.

#### DNA methylation in chemoresistance

DNA methylation constitutes a crucial mechanism within the domain of epigenetics, which exerts its regulatory function on gene expression through the attachment of methyl groups to DNA molecules. The role of DNA methylation in cancer chemotherapy resistance has been widely studied and concerned. DNA methylation has the potential to influence the expression of specific genes, thereby contributing to the development of drug resistance in tumor cells. In bladder cancer, merely 35% of metastatic bladder cancer patients initially show a response to cisplatin chemotherapy, and in the end, most bladder cancer patients who are sensitive to cisplatin chemotherapy develop resistance.^533^ High methylation of TLX3, TAp73, and HOXA9 promotes the anti-apoptotic effects of bladder cancer cells, while high methylation of SOCS3, STST3 and SOX2 promotes the development of cancer stem cells, leading to cisplatin resistance.^534,535^ Hypermethylation of the DNA repair protein MLH1 hypermethylation results in platinum resistance in ovarian cancer through reduced MLH1/c-Abl apoptotic signaling.^536^ Additionally, MLH1 hypermethylation causes an increase in drug resistance in CRC and is responsible for enhanced resistance by means of the loss of DNA mismatch repair capability, which causes genomic instability^537^ (Fig. 5). Besides apoptosis, DNA methylation plays a significant role in causing drug resistance in cancer cells through the modulation of the expression of transporters and metabolism-related genes. In HCC, the hypomethylation of drug delivery gene (ABCB1, ABCC2, ABCC5 and ABCG2) promoters can enhance the expression of these genes. This, in turn, boosts the cells’ ability to pump out drugs, consequently reducing the concentration of sorafenib within tumor cells and attenuating the cytotoxicity of sorafenib and hypomethylation and subsequent upregulation of ABCB1 and ABCG2 promote paclitaxel resistance in ovarian cancer.^538^ Beside, DNA hypomethylation of around the transcriptional start site of human organic cation transporter-1 (hOCT1, gene *SLC22A1*) decreases the uptake of quinine-inhibitable sorafenib by hepatoma cells, ultimately leading to sorafenib resistance in liver cancer cells^539^ (Fig. 5). Overall, DNA methylation plays a multifaceted role in various cancers by impacting gene expression patterns and contributing to the development of drug resistance. This underlines its critical importance in the pursuit of understanding and potentially surmounting the challenges posed by chemotherapy.

#### RNA modifications in chemoresistance

Epigenetic modifications of RNA are intricately linked to the sensitivity to various chemotherapy drugs.^540^ In particular, m^6^A modification is pivotal in modulating apoptosis, cancer cell migration, and invasion, thereby affecting chemotherapy resistance. In recent years, the connection between m^6^A modification regulators and chemoresistance has garnered significant attention and progress.^476,541–543^ HNRNPA2B1 is an RNA-binding protein that is upregulated in gastric cancer that has developed resistance to chemotherapy drugs. This protein stabilizes lncRNA NEAT1 through m^6^A modification, thereby activating the Wnt/β-catenin signaling pathway. The activation of this pathway endows gastric cancer cells with stem cell characteristics and enhances their resistance to 5-fluorouracil (5-FU).^544^ METTL3, an m^6^A “writer,” affects chemotherapy resistance by regulating autophagic degradation. LncRNA ARHGAP5-AS1 can recruit METTL3 to promote m^6^A modification of ARHGAP5 mRNA, maintaining its stability in the cytoplasm and preventing it from being degraded by autophagy, which leads to resistance to cisplatin.^545^ Additionally, METTL3 can induce chemotherapy resistance by triggering DNA damage repair. In breast cancer cell lines MCF-7 and MDA-MB-231, METTL3 augments resistance to doxorubicin (ADR) by improving the efficiency of HR and alleviating ADR-induced DNA damage.^546^ In MCF-7 cells that have developed resistance to ADR, the expression level of METTL3 is elevated. Furthermore, METTL3 also promotes the maturation of pri-miR-221-3p through m^6^A modification, which is associated with ADR resistance.^547,548^ In addition to m^6^A “writers,” “erasers” such as ALKBH5 also play an important role in chemotherapy resistance. ALKBH5 promotes the demethylation of WIF-1 mRNA, enhances its transcription, and inhibits the Wnt signaling pathway in AsPC-1 and PANC-1 cells, thereby increasing the sensitivity of pancreatic cancer cells to gemcitabine.^549^ Moreover, ALKBH5 affects chemotherapy resistance by regulating the stemness of cancer cells. Through m^6^A demethylation, ALKBH5 also stabilizes FOXO1 mRNA, leading to elevated levels of FOXO1 protein. This upregulation increases the expression of superoxide dismutase 2 (SOD2), diminishing intracellular ROS, and preserving the stemness of cancer cells as well as resistance to doxorubicin.^228^ In the treatment of ovarian cancer, elevated levels of ALKBH5 were identified to enhance the multiplication of epithelial cells and tumor growth, leading to resistance to cisplatin.^550^ Furthermore, the interaction between ALKBH5 and the homeobox A10 can inhibit the degradation of the JAK2/STAT3 signaling pathway mediated by YTHDF2, a key factor in the development of cisplatin resistance.^550^ Concurrently, another demethylase, FTO, through its interaction with lncRNAs adjacent to X-inactive specific transcript, facilitates the demethylation process of phosphoinositide-dependent kinase-1, which not only promotes aerobic glycolysis in glioma cells but also strengthens their resistance to temozolomide.^551^ The role of m^6^A reader proteins in chemoresistance is gradually being recognized by the scientific community. The latest research highlights the importance of elevated YTHDF1 in preserving the stem cell-like characteristics of cisplatin-resistant ovarian cancer cells. By promoting the translation of TRIM29 mRNA, YTHDF1 enhances the colony formation, spheroid formation capabilities, and invasiveness of cisplatin-resistant SKOV3/DDP and A2780/DDP cells^239^ (Fig. 5). This finding is significant for understanding the mechanisms of ovarian cancer therapy resistance and developing new therapeutic strategies.

In addition to the aforementioned m^6^A modification, m^5^C and m^7^G modifications also play significant roles in chemoresistance. In esophageal squamous cell carcinoma (ESCC), the m^5^C methyltransferase NSUN2 can upregulate the expression of TIGAR and GRB2, which increases the production of glutathione, reduces cell apoptosis, and promotes cell proliferation, collectively leading to chemoresistance in ESCC.^552^ Studies have also found that m^5^C reader proteins are associated with chemoresistance. In ovarian cancer patients, YBX1 modulates the expression of multiple downstream targets, including AKT, thereby promoting tumor proliferation and enhancing resistance to paclitaxel.^302^ Compared to non-resistant HCC cases, YBX1 expression is increased in chemotherapy-resistant HCC, indicating that YBX1 plays a key role in the observed chemoresistance in HCC.^552^ The m^7^G modification affects chemoresistance by regulating DNA damage repair, cancer migration, and invasion^309,321,553,554^ (Fig. 5). The m^7^G “writer” METTL1 suppresses the expression of S100A4 through m^7^G modification and upregulates miR-149-3p via a p53-dependent mechanism, thereby reducing the chemoresistance of CRC cells to cisplatin.^548^ Furthermore, the silencing of METTL1, which also methylates tRNAs at the variable loop of several tRNAs, has been shown to increase the sensitivity of cancer cells to 5-FU by increasing cell death^555,556^ (Fig. 5). These findings provide novel insights into the role of chemoresistance due to RNA modifications, which pave the way for developing new strategies to overcome resistance.

#### Non-coding RNAs in chemoresistance

##### LncRNAs

LncRNAs are frequently dysregulated in a diverse range of malignancies and engage in interactions with numerous RNAs and proteins, thereby exerting an impact on chemoresistance. LncRNAs regulate chemoresistance in cancer through a variety of molecular pathways including suppression of apoptosis, DNA damage response, multidrug efflux, EMT, as well as functioning as competitive endogenous RNA. When integrated with other regulatory mechanisms, these processes converge to form a highly intricate network of signaling that ultimately drives chemoresistance.^557^ LncRNAs can serve as molecular sponges for miRNAs, modulating miRNA activity and consequently activating signaling pathways associated with chemotherapeutic drug resistance, such as Wnt/β-catenin,^558^ PTEN/AKT,^559^ PTEN/PI3K^560^ and MAPK/ERK pathways. Additionally, the same lncRNA could regulate drug resistance in tumor cells through multiple mechanisms. For example, the lncRNA PVT1 promotes anti-apoptosis and chemotherapy resistance in gastric cancer by upregulating Bcl2 expression.^561^ Meanwhile, silencing lncRNA PVT1 with RNAi may hinder gastric cancer progression by increasing paclitaxel sensitivity.^562^ Additionally, PVT1 has been reported to act as a miRNA sponge, modulating miRNAs and their downstream signaling pathways to promote tumor drug resistance.^563^ For instance, VT1 may enhance cisplatin resistance in gastric cancer via the miR-3619-5p/TBL1XR1 axis and the miR-30a-5p/YAP1 pathway.^564,565^ Besides, lncRNAs can bind with RBPs to influence the DDR signaling pathway, regulating genes linked to cancer chemoresistance.^566^ Specifically, lncRNA SNHG12 enhances X-linked inhibitor of apoptosis protein (XIAP) transcription and stability by interacting with the RNA-binding protein Hu antigen R (HuR), promoting tumor growth and cisplatin resistance in NSCLC cells.^325^ LncRNA can also interact with other DDR-related protein to affect chemoresistance, including che-1, RBMX (RAN-binding motif protein X chromosome), PARP-1, YB-1 (Y-box protein 1).^567–569^ They also regulate efflux transporter activity, as seen with lncRNA linc00518 sponging miR-199a to affect ABCC1 expression, causing doxorubicin resistance in breast cancer cells.^90^ Recent studies indicate that lncRNAs are involved in the regulation of cancer-related stemness.^570,571^ Another study indicated that lncRNA NEAT1 was overexpressed in TNBC tissues and cell lines, and the silencing of NEAT1 was able to decrease stem cell populations, such as ALDH^+^, CD44^+^/CD24^−^, and SOX2^+^, and enhances the chemosensitivity of TNBC cells, including cisplatin and taxol.^572,573^ In addition, LncRNA SNHG14 contributed to trastuzumab tumorigenesis and resistance in breast cancer through the regulation of PABPC1 expression by H3K27 acetylation.^574^ Another lncRNA, HOTAIR, has been positively correlated with tamoxifen resistance, as it recruits the histone methyltransferase EZH2, which leads to tamoxifen resistance in breast cancer.^575–577^ Moreover, lncRNAs can perform a variety of functions in response to diverse environmental stimuli through dynamic structural changes. LncRNA LINC01956 undergoes dynamic structural remodeling that enhances the recruitment of O6-methylguanine DNA methyltransferase (MGMT) mRNA expression, which promotes DNA damage repair and tumor proliferation.^578^ LncRNAs can not only be regarded as potential biomarkers for the early diagnosis of cancer patients, but their expression profiles may also assist in predicting the sensitivity of cancer cells to different chemotherapy drugs, thereby alleviating chemoresistance (Fig. 5). the identification of lncRNAs that are uniquely expressed in chemoresistant cells can offer novel therapeutic alternatives for circumventing chemoresistance and cancer recurrence. Nevertheless, further research is requisite to validate the roles of the relevant lncRNAs.

##### miRNAs

miRNAs assume a crucial and pivotal role in the context of chemoresistance. A growing body of evidence have suggested that in various types of tumors, the expression levels of specific miRNAs are intricately associated with resistance to chemotherapeutic drugs. For instance, in prostate cancer, the upregulation of miR-200b-3p, miR-375, and miR-34b-3p may be associated with resistance to paclitaxel.^579^ Studies have revealed that miRNAs have the capacity to modulate the responsiveness of tumor cells to chemotherapeutic agents by engaging specific signaling cascades. An illustrative example is miR-99b-3p, which specifically targets the PPP2CA gene, consequently augmenting the phosphorylation of the AKT/mTOR signaling pathway. This modulation of the signaling cascade can substantially influence cell migration and potentially lead to the development of resistance to paclitaxel in breast cancer cells.^580^ Likewise, in HCC, miR-122 and miR-181a may facilitate resistance to sorafenib by modulating the RAS/RAF/ERK signaling pathway.^581^ MiR-31-5p, via extracellular vesicles (EVs), modulates the Hippo signaling pathway across diverse cell types within the tumor microenvironment, thereby contributing to the chemoresistance of pancreatic cancer cells to gemcitabine.^582^ MiRNAs can also influence the expression of target genes by binding to mRNA, thereby inhibiting translation or promoting degradation, which in turn affects the responsiveness of tumor cells to chemotherapeutic agents. Specifically, miR-223 downregulates the expression of FBXW7, resulting in resistance to doxorubicin in CRC^583^ (Fig. 5). Moreover, miR-223 suppresses the expression of FOXO3, contributing to the resistance of prostate cancer to docetaxel treatment and, through related mechanisms, also inducing cisplatin resistance in pancreatic cancer.^584^ Similarly, miR-21 downregulates the human DNA MutS homolog 2 (hMSH2) in CRC cells, thereby inducing resistance to 5-FU.

Moreover, miRNAs are capable of exerting an impact on the efficacy of chemotherapy by regulating the generation of tumor stem cells. In HCC, miR-2117 is found to be downregulated, and this downregulation serves to facilitate the self-renewal of liver tumor stem cells as well as tumorigenesis, ultimately culminating in cisplatin resistance.^585^ In NSCLC, miR-3163 targets the EXO1/Polη/Polι axis, inhibiting the growth of cancer stem-like cells in NSCLC and inducing apoptosis, thereby increasing the resistance of these cells to cisplatin.^586^ In NSCLC, miR-3163 targets the EXO1/Polη/Polι axis, inhibiting cancer stem-like cell growth and inducing apoptosis, which increases resistance to cisplatin. miRNAs can regulate gene transcription and epigenetics, affecting tumor cell response to chemotherapy. miRNA-381 may target multidrug resistance proteins (ABCG2, ABCC3, ABCC5) and stemness factors, enhancing glioblastoma sensitivity to temozolomide,^587^ while reducing the molecular level of miRNA-318 can lead to drug resistance in glioblastoma. miRNAs influence intracellular processes like apoptosis and DNA repair, affecting tumor cell drug resistance. For instance, increased miR-21 inhibits apoptosis in glioblastoma, causing sunitinib resistance.^588^ In NSCLC, miR-223 targets FBXW7, which then boosts autophagy and EMT, ultimately leading to resistance to both cisplatin and doxorubicin^589^ (Fig. 5). These research findings highlight the intricate and multifunctional role of miRNAs for chemoresistance and offer promising targets for the creation of innovative therapeutics, which holds significant implications for future cancer treatment strategies.

##### CircRNAs

Accumulating evidence has underscored the essential regulatory function of circRNAs in the carcinogenesis and progression of tumors. CircRNAs also play a role in tumor chemoresistance. CircRNAs can function as miRNA sponges and regulate chemosensitivity via the competitive endogenous RNA (ceRNA) mechanism. For instance, in osteosarcoma, circ_0004674 can sponge miR-142-5p, upregulate the anti-apoptotic protein MCL1 of the Bcl-2 family, leading to osteosarcoma progression and doxorubicin resistance.^590^ In laryngeal squamous cell carcinoma (LSCC), circPARD3 sponges miR-145-5p to activate the PRKCI-Akt-mTOR pathway and inhibit autophagy, promoting tumor cell proliferation, migration, invasion, and cisplatin resistance.^591^ Moreover, circRNAs regulate the sensitivity to chemotherapy drugs by directly interacting with proteins. In bladder cancer, circPTK2 binds with PABPC1, enhancing its ability to stabilize SETDB1 mRNA, promoting SETDB1-mediated EMT, significantly increasing in vitro migration and invasion capabilities, as well as gemcitabine resistance.^592^ CircIPO7 binds with the cytoplasmic Y-box binding protein-1 (YBX1), activating AKT phosphorylation to promote YBX1 nuclear translocation, increasing nasopharyngeal carcinoma’s resistance to cisplatin treatment.^593^ CircRNAs also affect the polarization of tumor-associated macrophages, thereby regulating chemoresistance. In ovarian cancer, circITGB6 directly interacts with IGF2BP2 and FGF9 mRNA, stabilizing FGF9 mRNA and inducing TAM polarization towards the M2 phenotype, increasing ovarian cancer’s resistance to cisplatin^594^ (Fig. 5). CircRNAs promote cell proliferation and induce tumor chemoresistance by activating related signaling pathways. For example, circTRIM1 enhances MARCKS translocation and activates the PI3K/AKT/mTOR signaling pathway, promoting chemoresistance to doxorubicin in TNBC.^595^ CircRNAs also regulate chemosensitivity by affecting intracellular molecular processes, such as apoptosis, autophagy, and DNA damage repair. The novel protein encoded by circATG4B can promote autophagy, inducing oxaliplatin resistance in CRC.^596^ CircPDIA3 can inhibit proptosis, thereby promoting oxaliplatin resistance in CRC^597^ (Fig. 5). In summary, circRNAs play a complex and crucial role in tumor chemoresistance, affecting the sensitivity of tumor cells to chemotherapy drugs through various mechanisms, including miRNA sponge function, direct interaction with proteins, affecting tumor-associated macrophage polarization, and regulating intracellular molecular processes. These discoveries offer an important theoretical foundation for the formulation of new cancer treatment strategies centered around circRNAs. They are anticipated to create new paths for future tumor treatment.

##### piRNAs

The aberrant expression of piRNA has been reported to promote chemoresistance across various cancer types.^598–602^ Numerous studies have shown that piRNAs and PIWILs are linked to key cancer traits, such as cell proliferation, evasion of cell death, metastasis, invasion, cell cycle regulation, all of which are linked with chemotherapy resistance^393^ (Fig. 5). For example, piR-1919609 and PIWIL2 was observed to be significantly upregulated in platinum-resistant ovarian cancer tissues. This overexpression is significantly linked to a poor prognosis adverse prognosis and a reduced recurrence-free survival period in ovarian cancer patients. Further investigations revealed that piR-1919609 primarily confers resistance to DDP by promoting cancer cell proliferation and inhibiting apoptosis.^598^ Similarly, studies have indicated that piR-39980 facilitates cellular motility and invasiveness by the stimulation of MMP-2, while concurrently inhibiting apoptosis via the negative regulation of SERPINB1.^601,602^ This dual mechanism plays a role in the development of chemoresistance in neuroblastoma and osteosarcoma cells. Additionally, a piRNA-like small RNA, piRNA-Ls, has been shown to induce chemoresistance to DDP-based therapy by suppressing apoptosis in lung squamous cell carcinoma.^600^ A recent study found that piR-FTH1 is frequently downregulated in six human cancer cell lines, with high Fth1 expression linked to doxorubicin resistance. Introducing external piR-FTH1 reduced Fth1 mRNA via HIWI2 and HILI, increasing TNBC cells’ doxorubicin sensitivity by 20-fold.^599^ In summary, piRNAs have been shown to regulate the expression of tumorigenic genes and cancer-suppressing genes at the transcriptional or post-transcriptional level by interacting with PIWI, thereby influencing the development of chemoresistance in various cancers. In summary, piRNAs interact with PIWI to regulate cancer-inducing genes and tumor-suppressing genes, affecting cancer chemoresistance (Fig. 5). They also recruit epigenetic writers, like histone-modifying enzymes and methyltransferases, to modulate DNA and m^6^A methylation and histone modifications, influencing key signaling pathways associated with therapeutic resistance. The crosstalk between these mechanisms will be further discussed.

### Immunotherapy

Immunotherapy has emerged as a truly groundbreaking advancement in cancer treatment, for it triggers the patient’s immune system to identify and assault tumor cells. Immune checkpoint inhibitors, such as drugs targeting PD-1, PD-L1, and CTLA-4, block the immune evasion mechanisms of tumor cells, thereby enhancing the anti-tumor activity of immune cells.^84,603–606^ Additionally, technologies like adoptive cell transfer immunotherapy offer a variety of treatment options.^607^ Despite these advancements, resistance to immunotherapy has emerged as a significant focus of contemporary research. Epigenetic modifications are pivotal in this context, contributing to the tumor’s resistance to immune checkpoint inhibitors and other immunotherapies by modulating immune cell function, influencing the tumor microenvironment, and regulating gene expression.^608,609^ Acquiring a comprehensive understanding of these complex epigenetic regulatory networks is crucial for developing novel cancer treatment strategies. For instance, integrating epigenetic therapy with immunotherapy holds promise for enhancing the therapeutic response rates in cancer patients and potentially prolonging their survival, thereby offering increased hope for patient outcomes. In the subsequent text, we will provide a detailed examination of the specific effects of the aforementioned epigenetic modifications on resistance to immunotherapy.

#### Histone modifications in immunotherapy resistance

The intricate relationship between histone modification and immunotherapy resistance has been gradually explored. Recent studies have shown that histone acetylation could regulate the expression of immune checkpoints, helping tumors evade immune system surveillance and influencing the infiltration of immune cells in the tumor microenvironment. For example, HDACi can increase the levels of PD-1 ligands in melanoma, thereby enhancing the immunotherapeutic effects of PD-1 blockers.^610^ Moreover, HDACi can upregulate PD-L1 mRNA and protein expression in a time-dependent manner in TNBC cells and significantly enhance the in vivo response to PD-1/CTLA-4 blockade in the triple-negative 4T1 breast cancer mouse model.^610^ HDACi enhance the immunotherapy effect in TNBC by regulating tumor growth. As an HDAC inhibitor, SAHA effectively inhibits tumor growth and significantly extends overall survival in immunocompetent GBM intracranial xenograft mouse models. Additionally, SAHA suppresses the activity of regulatory T cells (Treg) by targeting the c-Myc/CCL1 signaling pathway in glioma stem cells, thereby enhancing the efficacy of PD-L1 blockade therapy^611^ (Fig. 6).

Histone methylation, like acetylation, plays a crucial role in the regulation of PD-L1 expression. Research has found that the histone demethylase LSD1 is a regulator of PD-L1 expression, exhibiting varying regulatory patterns across different types of cancer.^612^ In CRC cells, the expression of LSD1 can impair the TCF1^+^PD-1 precursor cell subset of CD8+ T cells in the tumor microenvironment, thereby increasing the body’s resistance to PD-1 therapy.^613,614^ Furthermore, high expression of LSD1 in tumors can also promote the expression of T-cell exhaustion markers, including PD-1, CTLA4, TIM3, and TIGIT, indicating that LSD1 expression contributes to T-cell exhaustion and leads to immune evasion. In clinical treatment, combination therapy with LSD1 inhibitors and PD-L1 has successfully helped many patients overcome resistance to PD-1 blocking antibodies.^615,616^ On the other hand, high expression of PRMT1 or PRMT5 is negatively correlated with the immune activation of CD8+ T cells and natural killer (NK) cells, but positively correlated with the infiltration of myeloid-derived suppressor cells (MDSCs) and cancer-associated fibroblasts (CAFs), suggesting an immunosuppressive microenvironment.^617^ Therefore, targeting PRMT1 and PRMT5 is expected to become a new approach to overcoming resistance to immunotherapy (Fig. 6).

Histone lactylation is also reported to be linked to immunotherapy resistance. H3K9la has been identified as a specific modification site in HNSCC. It is widely recognized that IL-11 transcriptionally activates immune checkpoint genes via JAK2/STAT3 signaling in CD8^+^T cells. In a recent study, H3K9la positively correlates with IL-11 expression and unfavorable immunotherapy responses in patients.^618^ Thus, targeting H3K9la may be favorable to activate CD8+ T cells and reduce immunotherapy resistance (Fig. 6). These research findings underscore the importance of histone modifications in tumor immune evasion and imply that aiming at histone modifications may be a promising new approach to overcome resistance to immunotherapy.

#### DNA methylation in immunotherapy resistance

DNA methylation predominantly manifests its influence on conferring resistance to immunotherapy through its regulatory mechanisms governing tumor immune evasion. Extensive studies have demonstrated that DNA methylation can exert an impact on the escape of tumor cells from the immune surveillance through modulating the transcription of immune checkpoint molecules. In esophageal malignancy (EC), excessive hypomethylation of well-recognized biomarkers, such as PD-L1 and HER2, has given rise to their overexpression within the tumor microenvironment, thereby endowing it with immunosuppressive characteristics.^619,620^ DNA methylation indeed has the capacity to influence the transcription of PD-L1, thereby fostering resistance to immunotherapy. In the context of glioblastoma, the EZH2/H3K27Me3/DNMT1 complex orchestrates the methylation of the AP-2α gene, inhibiting its transcriptional activity. This diminished expression of AP-2α is associated with elevated levels of PD-L1 in high-grade glioma tissues. Elevated expression of PD-L1 facilitates the evasion of tumor cells from T-cell-mediated immune surveillance.^621^ In melanoma, genome-wide hypomethylation induces the upregulation of PD-L1 and the secretion of inhibitory cytokines, concomitant with EMT alterations that collectively foster an immunosuppressive microenvironment.^622,623^ DNA methylation may also affect the expression of tumor-associated antigens, leading to immune evasion. Hypermethylation of the transporter 1 ATP-binding cassette subfamily B member (TAP1) has been shown to suppress TAP1 expression in immunoreactive cancer stem cells (CSCs) within murine models of breast carcinoma, these CSCs manifest a downregulation in the expression of transporter associated with antigen processing genes and co-stimulatory molecules, thereby reducing their sensitivity to T-cell-mediated immune surveillance and potentially culminating in immunotherapy resistance.^624,625^ Additionally, the methylation of chemokines can also lead to resistance to immunotherapy. In a murine model of hepatocellular carcinoma, the hypermethylation of CXCL9 and CXCL10 promoter leads to reduced expression of these chemokines. The downregulation of CXCL9 and CXCL10 reduces the count of T cells that infiltrate the tumor, thereby increasing the risk of resistance to immunotherapy.^626^ A study has developed an index of methylation-based epigenetic silencing (iMES) signature. They found that high iMES is linked to VEGF pathway silencing, endothelial cell attenuation, immune modulation, EZH2 induction, BAP1/SETD2 depletion, and resistance to immune checkpoint inhibition (ICI).^627^ Besides, the DNA methylation of related molecules may also affect the infiltration of immune cells, leading to resistance to immunotherapy. Hypermethylation of ACAP1 is negatively correlated with the infiltration level of tumor-infiltrating lymphocytes, thereby reducing the sensitivity to immunotherapy^628^ (Fig. 6). A thorough elucidation of these methylation-mediated mechanisms is essential for formulating approaches to surmount immunotherapy resistance and augment efficacy for cancer immunotherapy in the future.

#### RNA modifications in immunotherapy resistance

Recent studies have underscored the pivotal function of RNA modification regulators in regulating the response to immune checkpoint blockade therapy. Numerous investigations into m6A modification have elucidated its significant association with resistance to immune checkpoint inhibitors, particularly those targeting the PD-1/PD-L1 axis.^222,629,630^ The overexpression of METTL3 in thyroid cancer cells can enhance the efficacy of anti-PD-1 therapy. In a recent study, it was found that the downregulation of METTL3 promotes the demethylation of CD70 mRNA, leading to an increase in the number of regulatory T cells (Tregs) with immunosuppressive functions and terminally exhausted T cells, thereby enhancing the resistance to immune therapy.^631^ In addition, FTO is also a regulator of resistance to immunotherapy. Under metabolic stress, melanoma cells increase the level of FTO through autophagy and NF-κB signaling pathways, resulting in resistance to interferon-γ (IFN-γ) and anti-PD-1 treatment. In melanoma cells, knocking down the FTO gene can accelerate the RNA degradation rate of intrinsic pro-tumor genes including PD-1, CXCR4 and SOX10 through YTHDF2. Therefore, inhibiting FTO can reduce the resistance to immune therapy^632^ (Fig. 6).

Targeting m^5^C represents a novel approach in the field of mRNA-based therapeutics. m^5^C-modified mRNA can reduce immune recognition by inhibiting Toll-like receptor 3 (TLR3) in the endoplasmic reticulum, thereby promoting immune evasion.^633^ In immunologically “cold” tumors, the activation of the cGAS/STING axis (cyclic GMP-AMP synthase/STING axis) not only promotes tumor formation but also enhances resistance to PD-1 immunotherapy. NSUN2, known as an eraser of m^5^C, can suppress the cGAS/STING signaling pathway when overexpressed, thereby reducing the immunotherapy resistance of tumors.^634^ NSUN2 methylates ICAM-1 mRNA and promotes its translation, inhibiting the polarization of M2-type macrophages, which in turn reduces tumor metastasis and enhances the effectiveness of immunotherapy. Additionally, targeting the expression of NSUN2 may improve the immunotherapy outcomes in HNSCC.^635,636^ The knockout of METTL1 (m^7^G writer) significantly improves the efficacy of anti-PD-1 treatment for intrahepatic cholangiocarcinoma, indicating that modulating mRNA methylation can enhance the effects of immunotherapy^556^ (Fig. 6). Furthermore, the knockout of YBX1 reverses resistance to immunotherapy by blocking PD-L1 expression and activating T cells in the tumor microenvironment, providing a new strategy for immunotherapy.^637^

#### Non-coding RNAs in immunotherapy resistance

##### LncRNAs

LncRNAs are crucial in immunotherapy resistance, exerting an impact on a multitude of mechanisms and pathways. They are involved in the regulation of immune checkpoint molecules such as PD - L1, thereby facilitating tumor cells’ ability to evade the immune system. For example, lncRNA SNHG29 boosts PD-L1 expression by activating YAP, while LINC00460 acts as a molecular adsorbent for miR-186-3p, which results in an elevation for MYC, CD47, and PD-L1, consequently strengthening immune evasion in CRC cells.^638,639^ LncRNA MIR155HG modulates PD-L1 expression via m^6^A modification, aiding HCC’s immune escape.^640^ Additionally, lncRNAs can influence immune evasion by altering antigen expression. Cancer-associated fibroblast-derived EVs containing lncRNA RP11-161H23.5 reduce HLA-A expression in pancreatic carcinoma cells, facilitating tumor proliferation and immune evasion^641^ (Fig. 6).

Besides, lncRNAs exert a significant influence on various immune cells within the tumor microenvironment, such as T cells, myeloid-derived suppressor cells (MDSCs), and natural killer (NK) cells, thereby impacting their responsiveness to immunotherapy. Moreover, they are actively involved in multiple signaling pathways that contribute to the enhancement of regulatory T cells, ultimately creating an immunosuppressive milieu that favors tumor growth. For example, lnc-EGFR aids liver cancer cell growth by promoting Treg cell differentiation and suppressing CTL activity, facilitating immune escape.^642^ In breast cancer, increased lncRNA SNHG1 in CD4+ T cells boosts IDO expression by binding to miR-448, promoting Treg cell maturation and immune evasion.^643^ During the process of tumor immune escape, cytotoxic T lymphocytes (CTLs) may become dysfunctional or exhausted,^644^ and lncRNAs play a significant regulatory role in this process. For instance, lncRNA NEAT1 can impede the proliferation of CTLs by suppressing the cyclic GMP-AMP synthase (cGAS)/stimulator of interferon genes (STING) pathway, thereby promoting immune escape.^645^ Similarly, lncRNAs influence other innate immune cells, contributing to an immunosuppressive microenvironment and facilitating immune escape. For instance, LINC00963 can hinder the differentiation and maturation of dendritic cells by binding to miR-612, which assists gastric cancer cells in evading the immune system.^646^ LncRNAs also participate in the formation of resistance to immunotherapy by affecting various molecular pathways within tumor cells, such as regulating cell cycle, apoptosis, metabolism, and epigenetic modifications. For example, lncRNA TYMSOS promotes the growth and metastasis of breast cancer cells and leads to immune escape through the CBX3/ULBP3 or SYVN1/ULBP3 axis.^647^ Furthermore, lncRNAs partake in the formation of resistance to immunotherapy by modulating diverse molecular pathways within tumor cells, including those related to cell cycle regulation, apoptosis, metabolism, and epigenetic modifications. For instance, lncRNA TYMSOS enhances breast cancer growth and metastasis, facilitating immune evasion via the CBX3/ULBP3 or SYVN1/ULBP3 axis^648^ (Fig. 6). These studies underscore the intricate role of lncRNAs in immunotherapy resistance and point to potential molecular targets for novel treatment strategies. The aim of these strategies is to surmount therapeutic resistance and enhance clinical responses. Further studies should concentrate on delving deeper into precise mechanisms of lncRNAs to further enhance cancer therapy.

##### miRNAs

miRNAs are crucial in immunotherapy resistance, modulating immune responses and affecting tumor interactions. In HCC, hypoxic tumor-derived exosomal miR-1290 promotes the polarization of M2 macrophages, triggers apoptosis in CD8+ T cells, and enhances EMT in HCC cells. These effects collectively aid tumor cells in evading the immune system.^649^ In breast cancer, the downregulation of miR-299-3p leads to an upregulation of CD47 expression. This, in turn, inhibits the activation and infiltration of macrophages and promotes tumor growth, ultimately resulting in immune escape within breast cancer.^650^ Particularly, in TNBC, exosome-carried miR-20a-5p is internalized into CD8+ T cells, causing T-cell dysfunction and further conferring resistance to PD-1 therapy in TNBC.^651^ Moreover, miRNAs can control immune checkpoint molecules like PD-L1, aiding tumor cells in evading immune attacks. In NSCLC, an increased level of miR-142-5p suppresses PTEN while elevating the levels of PI3K, p-Akt, and PD-L1, thereby facilitating tumor immune escape.^652^ miRNAs also assist tumor cells in evading immune detection, which allows for their unrestrained growth. In CRC, exosome-derived miR-372-5p targets the PTEN/AKT/NF-κB pathway to induce immune escape^653^ (Fig. 6). Collectively, studies indicate that miRNAs play a multifaceted role in tumor immune escape by exerting an impact on both the processes of tumor cells and the functions of immune cells. As such, they represent potential targets for future tumor immunotherapy.

##### CircRNAs

CircRNAs contribute to tumor immune evasion by regulating immune responses. They enable tumor cells to elude immune detection, and one of the ways they achieve this through modulating transcription of PD-L1 via miRNA sponging. As for gastric cancer, circ_0136666 upregulates PRKDC by sponging miR-375-3p, which activates the PD-L1 phosphorylation pathway, preventing PD-L1 degradation and aiding immune evasion.^654^ In esophageal cancer, circ-VIM promotes immune evasion by increasing PD-L1 expression through miR-124 sponging.^655^ Similarly, in gastric cancer, hsa_circ_0001479 boosts DEK expression by sponging miR-133a-5p. This, in turn, activates the Wnt/β-catenin pathway, which not only promotes cell growth but also reduces the infiltration of CD8+ T cells, thus facilitating immune evasion.^656^ In ovarian cancer, circ-NFIX promotes tumor progression and immune evasion by sponging miR-647 and activating IL-6R/JAK1/STAT3 signaling.^657^ CircRNAs can also influence immune cell function in the tumor microenvironment. HCC cells release exosomal circCCAR1, which is absorbed by CD8+ T cells, stabilizing PD-1 protein and causing T-cell dysfunction, leading to immune evasion.^629^ In NSCLC, circIGF2BP3 enhances tumor immune evasion by promoting PD-L1 deubiquitination, inhibiting CD8+ T-cell responses.^658^ The human tumor virus-encoded circE7 hampers T-cell function by reducing LGALS9 transcription, aiding immune evasion in HNSCC.^659^ Additionally, circRNAs contribute to innate immunity; for instance, AML cell-derived exosomal circ_001264 fosters immune evasion by promoting M2 macrophage polarization and PD-L1 expression^660^ (Fig. 6). Additionally, exosome circUHRF1 released from cancer cells induces natural killer cell exhaustion, potentially giving rise to resistance to PD-1 therapy in HCC.^661^ CircRNAs can influence immunotherapy sensitivity by affecting tumor cell glycolysis (Fig. 6), as seen with bladder cancer circFAM13B, which inhibits glycolysis via the IGF2BP1/PKM2 pathway, thereby enhancing immunotherapy sensitivity.^662^

Overall, circRNAs contribute to tumor immune evasion by sponging miRNAs to regulate PD-L1 expression, impacting signaling pathways and immune cell functions, and modulating immune responses in the tumor microenvironment. These insights offer potential targets and a theoretical framework for future developments.

##### piRNAs

Recent investigations have shed light on the fact that piRNAs may play a role in contributing to immunotherapy resistance. Specifically, the piRNA piR-hsa-30937, found in small extracellular vesicles from pancreatic neuroendocrine neoplasms, can be released into the microenvironment and enhance CD276 expression in macrophages via the PTEN/AKT pathway, leading CD276^+^ TAMs to inhibit T-cell antitumor immunity^663^ (Fig. 6). These findings further emphasize the complexity of the mechanisms underlying immunotherapy resistance and highlight the importance of continued exploration into the roles of various non-coding RNAs like piRNAs. An in-depth understanding of these mechanisms contributes to novel strategies to overcome immunotherapy resistance and improve the effectiveness of cancer treatment in the future.

### Targeted therapy

Targeted therapy predominantly depends on advancements in tumor molecular biology and genomics to address specific molecules or signaling pathways that are integral to tumor initiation and progression. Its primary classifications include protein kinase inhibitors, agents targeting tumor metabolism, and inhibitors of DNA damage repair pathways.^664^ Epigenetic regulators frequently exhibit aberrant expression in tumors that have developed resistance to targeted therapy, highlighting their role in the emergence of resistance to such treatments.^665^ A wealth of research has demonstrated that epigenetic alterations could profoundly influence the targeted therapy resistance.^666^ These regulators facilitate therapy resistance by modulating tumor proliferation, migration, invasion, apoptosis, and other critical factors.^666^ Consequently, clinical trials are exploring the combination of small-molecule inhibitors targeting epigenetic modulators with targeted therapies as a promising strategy to surmount resistance to targeted treatments. (Table 1) In the following section, we will delve into the epigenetic factors that govern resistance to targeted therapies.

#### Histone modifications in targeted therapy resistance

Recent research indicates that histone modifications impact resistance to protein kinase inhibitors by regulating cancer cell migration, invasion, and apoptosis through the modulation of oncogene transcriptional activity.^84,667–670^ Specifically, HDAC11 enhances the self-renewal capacity of lung adenocarcinoma stem cells and contributes to resistance to EGFR inhibitors by promoting Sox2 expression.^671^ Additionally, the combined use of HDAC inhibitors and EGFR inhibitors shows potential as candidate drugs for cancer treatment by promoting apoptosis through the activation of caspase 3/7.^672^ In NSCLC, the expression level of EZH2 is negatively correlated with resistance to epidermal growth factor receptor tyrosine kinase inhibitors (EGFR-TKIs). Upregulating or promoting EZH2 expression can enhance the sensitivity of NSCLC cells to EGFR-TKIs^673^ (Fig. 7). Furthermore, targeting euchromatic histone-lysine N-methyltransferase 2 (EHMT2) can reverse EGFR-TKI resistance in NSCLC by epigenetically regulating the PTEN/AKT signaling pathway^674^ (Fig. 7). In conclusion, the targeting of histone PTMs, including HDACs and EZH2, represents a promising avenue for addressing drug resistance in cancer therapy. This approach not only augments the efficacy of current therapeutic agents but also offers innovative pathways for the development of novel anticancer drugs.

#### DNA methylation in targeted therapy resistance

DNA methylation exerts a significant impact on cellular apoptosis, consequently influencing the sensitivity towards targeted therapy. Elevated methylation in the death-associated protein kinase (DAPK) can give rise to resistance against anti-EGFR agents in NSCLC and HNSCC cell lines. This might be attributed to the fact that hypermethylation of DAPK attenuates pro-apoptotic signaling, thereby undermining the efficacy of anti-EGFR drugs^675^ (Fig. 7). DNA methylation is also capable of influencing the expression of cellular targets, thereby resulting in resistance to targeted therapy. In breast cancer, high methylation of the ESR1 promoter mediated by DNMTs leads to reduced ERα expression, resulting in tamoxifen resistance.^676,677^ Moreover, methylation can also lead to tamoxifen resistance by regulating the promoter methylation of ERα-related genes. High methylation of upstream genes of ERα suppresses ERα expression, thereby promoting tamoxifen resistance, such as p21, WT1, and miR-27b.^678–680^ High methylation of downstream genes of ERα leads to reduced expression, further leading to ERα dysfunction and inducing tamoxifen resistance, such as NAT1, ELOVL2 and PRA.^681,682^ Apart from ERα, methylation-induced inactivation of the PI3K/AKT/mTOR signaling pathway can enhance tamoxifen sensitivity.^683^ PTEN, being a crucial protein in the PI3K/AKT/mTOR signaling pathway, experiences increased AKT phosphorylation due to its hypermethylation, which consequently leads to tamoxifen resistance.^684^ However, the methylation of ERβ has an effect contrary to that of ERα. Hypomethylation of ERβ leads to tamoxifen resistance. DNA methylation can also affect the sensitivity to targeted therapy drugs by altering DNA repair related genes.^685^ For example, O-6-methylguanine-DNA methyltransferase (MGMT) is a key DNA damage repair gene, and low methylation of the MGMT promoter leads to reduced sensitivity to TMZ in GBM.^686^ Additionally, DNA methylation can impact the transport of targeted drugs into cells, thereby affecting drug sensitivity. OSCP1 encodes a widely substrate-specific organic solute carrier protein, and its high methylation restricts the transport of imatinib into nasopharyngeal cancer cells, resulting in imatinib resistance.^687^ During targeted drug therapy, DNA methylation can also affect cellular viability, giving rise to resistance to targeted therapy. In NSCLC, increased methylation of WIF promotes the cellular viability of gefitinib-resistant cells, further increasing cellular resistance^688^ (Fig. 7). Some tumor entities, like liver cancer, inherently exhibit resistance to TRAIL. In HCC, hypomethylation of Ache can impede the apoptosis of HCC cells induced by the cytokine TRAIL, leading to therapeutic resistance^689^ (Fig. 7). In summary, DNA methylation exerts its influence on the sensitivity to targeted therapy through a myriad of means. Gaining a comprehensive understanding of these mechanisms is of paramount importance for surmounting resistance and enhancing the effectiveness of cancer treatment.

#### RNA modifications in targeted therapy resistance

Recent research indicates that RNA modifications exert a crucial influence on modulating resistance to targeted therapies by influencing cellular processes such as apoptosis, proliferation, tumor migration, and invasion.^690^ Recent researches have shown METTL14 reduces the expression of HNF3γ mRNA through m^6^A modification, with HNF3γ being a key regulator of sorafenib resistance. The expression of HNF3γ not only promotes the differentiation of HCC cells, but also that of LCSCs. Therefore, the upregulation of METTL14 can lead to sorafenib resistance in liver cancer.^690^ In addition, autophagy mediated by FOXO3 results in sorafenib resistance of HCC. The deletion of METTL3 inhibits the m^6^A modification of FOXO3 mRNA via YTHDF1, thereby facilitating sorafenib resistance and cancer proliferation in HCC.^238^ Inhibiting the demethylase activity of FTO with Rhein can sensitize resistant cells to TKIs, downregulate Bcl-2 and MER proto-oncogene, tyrosine kinase (MERTK), thus regulating the PI3K-AKT-mTOR pathway and the Bcl-2 family of proteins, which affects the invasion and apoptosis of cancer cells.^691–693^ Therefore, the FTO-m^6^A axis has become a new indicator for TKI resistance. It has been reported that meclofenamic acid (MA), an FTO inhibitor, can also reverse TKI resistance.^691^ The inhibition of TSUC7 associated with m^6^A result in erlotinib resistance of lung cancer by regulating the stemness of EMT features in a Notch signaling activation-dependent manner.^694^ In A375, METTL3 augments the m^6^A modification of EGFR, boosts the RAF/MEK/ERK signaling pathway, and induces apoptosis, thereby promoting resistance to the BRAF (V600E) kinase inhibitor PLX4032^695^ (Fig. 7).

Recent studies have indicated that m^5^C modification is associated with resistance to targeted therapy.^696–698^ NSCLC with epidermal growth factor receptor (EGFR) mutations, m^5^C hypermethylation and NSUN2 are related to the intrinsic gefitinib resistance and tumor recurrence. The overexpressed NSUN2 interacts with YBX1, promoting the translation of quiescin sulfhydryl oxidase 1 (QSOX1) mRNA, thereby enhancing the resistance of non-small-cell lung cancer to gefitinib.^697^ M^7^G also shows relevant effects in the resistance to targeted therapy. In HCC, the elevation of m^7^G levels and the upregulation of METTL1 can lead to the resistance of HCC to lenvatinib.^309^ In lung adenocarcinoma, dual-specificity phosphatase 5 (DUSP5), regulated by YTHDF1-mediated m^6^A modification, promotes EMT and the resistance to epidermal growth factor receptor-tyrosine kinase inhibitor (EGFR-TKI) through the transforming growth factor-β (TGF-β)/Smad signaling pathway.^699^ The latest research on the combined strategy of RNA modification and targeted therapy covers multiple aspects, ranging from basic mechanism studies to clinical applications, demonstrating the great potential of RNA modification in cancer treatment and its synergistic effects with other therapies.

#### Non-coding RNAs in targeted therapy resistance

##### LncRNAs

LncRNAs are of vital significance in altering the sensitivity of cancer cells to targeted drugs by influencing molecule expression as well as signaling pathways. In NSCLC, decreased levels of lncRNA H19 are found in drug-resistant cells. The silencing of H19 can augment the expression of PKM2, and then contributing to erlotinib resistance in NSCLC^700^ (Fig. 7). Additionally, lncRNA UCA1 can cause erlotinib resistance by activating the AKT/mTOR pathway and inducing EMT, and it can also promote gefitinib resistance by binding to EZH2 and silencing CDKN1A epigenetically.^701^ Studies have demonstrated that LINC00665 can modulate SERPINE1 and enhance p-AKt expression within the PI3K/AKt pathway, resulting in trastuzumab resistance in gastric cancer.^702^ Additionally, lncRNAs could boost receptor signaling, which in turn heightens resistance to targeted therapies. For example, lncRNA HOTAIR is elevated in breast cancer, affecting estrogen receptor transcription and increasing tamoxifen resistance.^703^ LncRNAs can also affect the sensitivity of tumor cells to targeted therapy drugs by regulating cell cycle and apoptosis. For example, lncRNA PRNCR1 promotes the proliferation of breast cancer and inhibits apoptosis by regulating the microRNA-377/CCND2/MEK/MAPK axis, leading to resistance to tamoxifen in breast cancer^704^ (Fig. 7). LncRNAs can also influence the epigenetic modifications of specific molecules, causing changes in their levels, which affect the proliferation of cancer cells and mediate their drug sensitivity. For example, cyclin D1 is a key oncogene that promotes the proliferation of cancer cells. Studies have shown that in breast cancer, lncRNA DILA1 directly binds to cyclin D1, preventing its phosphorylation at the Thr286 site, thereby inhibiting its degradation and increasing the proliferation of breast cancer cells and promoting resistance to tamoxifen.^705^ Additionally, lncRNAs can act as competing ceRNAs, binding to specific miRNAs and thus alleviating the inhibition of their target mRNAs, which enables them to regulate the sensitivity to targeted therapy. In lung adenocarcinoma, lncRNA MALAT1 acts as a ceRNA, suppressing the expression of miR-125 and promoting the expression of Rab25, leading to resistance to EGFR-TKI (erlotinib) in lung adenocarcinoma.^706^ LncRNAs, like UCA1, can be secreted through exosomes, contributing to drug resistance in recipient cells (Fig. 7). In summary, lncRNAs are of vital importance in influencing the sensitivity of cancer cells to targeted drugs and contribute to drug resistance through multiple aspects, including molecular expression, signaling pathways, cell cycle regulation, apoptosis modulation, and epigenetic alterations. This underlines their intricate role in drug resistance and presents potential targets for novel therapies aimed at overcoming or reversing such resistance.

##### miRNAs

MiRNAs exert influence on contributing to resistance against targeted therapy by modulating the drug responsiveness of tumor cells via diverse mechanisms. They affect pathways and molecules involved in cell growth, impacting pathways like PI3K/AKT/mTOR, RAS/RAF/ERK, and STAT/JAK (Fig. 7). For instance, in gliomas, miRNA-7 exerts an inhibitory effect on IRS-1 and IRS-2, which are pivotal regulators within the IGF-1R/Akt pathway.^707^ In breast cancer, miRNA-205 could enhance tamoxifen resistance and proliferation by targeting E2F1.^708^ In CRC, miR-100 and miR-125b together activate the Wnt/β-catenin pathway, boosting resistance to cetuximab.^709^ Moreover, miRNAs have a bearing on receptor regulation and the sensitivity to therapy. In NSCLC, reduced miRNA-145 boosts EGFR, promoting cell growth and erlotinib resistance.^710^ Conversely, miR-145 inhibits platelet-derived growth factor receptor, decreasing proliferation and increasing erlotinib and gefitinib sensitivity.^711^ In breast cancer, miR-335-5p and miR-335-3p work in tandem to suppress estrogen receptor α, thereby heightening tamoxifen resistance.^712^ miRNAs influence therapy sensitivity by regulating processes like apoptosis, cell cycle, and EMT. In NSCLC, knockdown of miR-200c-3p enhances EMT, boosting resistance to erlotinib and gefitinib.^713^ MiR-146a-5p promotes migration and angiogenesis, causing trastuzumab resistance in HER2+ breast cancer^714^ (Fig. 7). In summary, miRNAs influence targeted therapy sensitivity by affecting signaling pathways, intracellular processes, and receptor regulation, highlighting their crucial role in tumor drug resistance and offering potential targets for new therapies.

##### CircRNAs

CircRNAs are increasingly for their influence in cancer treatment resistance, especially against targeted therapies. They exert an impact on biological functions through multiple modalities, such as functioning as miRNA sponges, influencing protein translation, or engaging in interactions with RBPs, thereby having a profound effect on therapy sensitivity. For example, circRNA_102481 acts as a sponge for miR-30a-5p to modulate the expression of ROR1. This regulatory action promotes cell proliferation while simultaneously inhibiting apoptosis, ultimately resulting in resistance to erlotinib and gefitinib in NSCLC^715^ (Fig. 7). In lung cancer, circRABL2B interacts with YBX1 to suppress MUC5AC, which in turn inhibits integrin β4/pSrc/p53 signaling as well as tumor stem cell characteristics, thereby augmenting erlotinib sensitivity.^716^ Additionally, circRNAs can influence targeted therapy sensitivity by binding to proteins. For instance, circ_SIRT1 binds to EIF4A3, upregulating ATG12, which enhances autophagy and imatinib resistance in chronic myeloid leukemia (CML).^717^ Reports suggest that certain circRNAs can trigger signaling pathways causing resistance to targeted therapies. In breast cancer, increased circCDYL2 stabilizes GRB7, preventing its degradation and boosting its interaction with FAK, which sustains AKT and ERK1/2 pathway activity, leading to trastuzumab resistance.^718^ Conversely, the reduction of certain circRNAs is likely to cause treatment resistance. For instance, in osimertinib-resistant lung adenocarcinoma, where decreased circFBXW7 activates the Wnt/β-catenin signaling pathway, further promoting proliferation and resistance to osimertinib^719^ (Fig. 7).

Certain circRNAs exert an influence on treatment sensitivity by modifying tumor cell metabolism as well as cell death pathways. For instance, circHIF1A enhances glycolysis changes, leading to cetuximab resistance in CRC.^720^ CircRNAs can also regulate treatment sensitivity by modulating cell death pathways. In breast cancer, the newly identified circVDAC3 confers resistance to trastuzumab by preventing the ubiquitination of HSPB1, reducing ferroptosis^721^ (Fig. 7). Similarly, circ-BGN can promote resistance to trastuzumab in breast cancer by enhancing OTUB1-mediated SLC7A11 deubiquitylation and alleviating ferroptosis.^722^ CircRNAs are of pivotal significance in modulating the sensitivity and resistance to cancer treatment, thus presenting novel therapeutic strategies and potential biomarkers for further exploration in the field of oncology.

### Crosstalk between different epigenetic systems

Building on the discussion of single epigenetic mechanisms and their roles in tumorigenesis and therapeutic resistance, we now shift our attention to the intricate interplay between these various forms of epigenetic regulation. This crosstalk is crucial, as it underlies the complex gene expression patterns that fuel cancer progression and shape the resistance to therapies. Comprehending the interplay and cooperation among various epigenetic modifications is crucial for unraveling the full spectrum of their contributions to cancer biology. Epigenetic modifications, such as DNA methylation, histone post-translational modifications, and non-coding RNAs, operate in a sophisticated network that orchestrates gene expression patterns, thereby influencing cellular processes and contributing to the hallmarks of cancer.^23,723,724^ The crosstalk between these epigenetic markers is of particular importance, since it may lead to the activation of oncogenes or the suppression of tumor suppressor genes, which in turn modulates cancer cell behavior and response to therapeutic interventions.^725,726^

The interplay between DNA methylation and histone modifications, for instance, has been shown to create a repressive chromatin environment capable of silencing tumor suppressor genes, thereby promoting oncogenic transformation.^727^ Non-coding RNAs, including miRNAs and lncRNAs, also participate in this crosstalk by regulating the expression of genes involved in the epigenetic machinery, further shaping the epigenetic landscape of cancer cells.^55^ Moreover, the tumor microenvironment (TME) is replete with cellular and molecular components that can influence epigenetic marks on cancer cells, thereby affecting their phenotype and therapy response. The communication between cancer cells and the TME can lead to changes in the epigenome that reinforce the malignant properties of cancer cells and their resistance to treatment.

In the forthcoming sections, we will explore the intricate relationships between different epigenetic modifications and their collective impact on cancer progression and therapy resistance. By examining the latest research, we aim to provide insights into how these epigenetic interactions can be targeted to improve cancer treatment outcomes.

#### Histone modifications and DNA methylation

As we began to unravel the vast and intricate network of epigenetic modifications, we decided to find the entry point according to the levels of gene expression and gene regulation.^728^ It became apparent that the histone modifications are exactly the crucial key to unlocking this door, as they regulate genes at the chromatin level with global implications, and histone modification can serve as an anchor for other types of epigenetic modification or modifying enzymes.^158^ The interactive relationship between histone modifications and DNA methylation constitutes a significant component of epigenetic crosstalk.^105^ First, histone-modifying enzymes can directly act on DNMTs to regulate methylation levels. For example, the methylation of DNMT1 mediated by SET7/9 leads to a decrease in its stability and subsequent degradation, thereby causing a reduction in methylation levels across the entire genome.^729^ Second, previous studies have indicated that histone modifications can regulate the catalytic activity of DNMTs by the recruitment or dissociation of them.^730^ For instance, DNMT3A, DNMT3B and DNMT3L process an ADD (ATRX-DNMT3-DNMT3L) domain that interacts with unmodified N-terminal tails of histone H3, but H3K4me1/me2/me3 iteratively weakens this interaction and inhibits DNMT3A/B catalytic activity.^731–733^ Similarly, H3K36me2 can recruit DNMT3A and shapes the intergenic DNA methylation landscape.^730^ The interaction between histone methylation modification and DNA methylation has been well summarized in recent reviews.^734–736^ Third, the methylated DNA and histone-modifying enzymes can form complexes through certain “intermediate” proteins. For example, the methylated DNA may be recognized and bound by proteins known as MBDs.^190,737^ MBD proteins attract other proteins to the site, such as HDACs and other chromatin remodeling proteins capable of modifying histones, thereby creating compact, transcriptionally inactive heterochromatin.^32,189^

In summary, the collaboration between histone PTMs and DNA methylation for silencing of gene expression has been well-documented,^738,739^ and Importantly, such crosstalk/collaboration between them has been shown to promote therapeutic resistance in cancer.^740^ Tumor cells can invoke these epigenetic tools to synergistically achieve drug resistance.^740^ For example, clinical evidence indicates that BRAF^V600E^-mutated CRC shows a strong correlation with high levels of DNA methylation.^740^ When BRAF-mutated CRC exhibits a CpG island hypermethylation phenotype (CIMP-H), it often presents with therapeutic resistance and poor clinical prognosis.^741^ It has been confirmed that treatment with 5-azacytidine significantly reduces the level of DNA methylation in tumor cells, but the transcriptional repression of key tumor suppressor genes was not relieved. The increase in the histone deacetylation and histone methylation (H3K4me3 and H3K27me3) around the hypomethylated regions suggested that tumor cells could mobilize histone modifications to continue suppressing genes after DNA methylation was inhibited by the 5-azacytidine.^740^ Their work suggested that compensatory epigenomic alterations predominantly triggered by H3K27me3-based silencing following treatment-induced DNA methylation reduction, laying the groundwork for a potential DNMT and EZH2 inhibitor combination therapy in BRAF^V600E^ CRC.

In recent years, it has been reported that the dysregulation of both histone modifications and DNA methylation often occurs simultaneously in tumors.^609,742^ For instance, the overexpression of DNMTs and HDACs in breast cancer tissues is positively correlated with each other and is associated with poor prognosis.^743^ Therefore, the combined use of DNMTi and HDACi shows great potential in overcoming resistance to cancer therapy. Chang et al. obtained a series of DNMT1/HDAC dual inhibitors by fusing the key pharmacophores from DNMT1 inhibitors (DNMT1i) and HDAC inhibitors (HDACi). Among them, compound (R)-23a demonstrates significant DNMT1 and HDAC inhibition both in vivo and in cells (colorectal tumor) and largely phenocopied the synergistic effects of combined DNMT1i and HDACi in reactivating epigenetically silenced tumor suppressor genes (TSGs).^744^ Huang et al. designed a dual DNMT and HDAC inhibitor (termed DNMT/HDACi) 15a, which exhibits immunomodulatory functions by increasing the intracellular level of double-stranded RNA to activate the RIG-I/MAVS pathway and enhances the effectiveness of immune checkpoint blockade therapy.^743^

#### Histone modifications and m^6^A RNA modification

Recent studies and reviews have highlighted the intricate crosstalk between histone modifications and m^6^A RNA modifications.^745–747^ Histone modifications can affect the local enrichment of m^6^A modifications by recruiting or releasing m^6^A writers, erasers, and readers.^748,749^ For example, it has been disclosed that H3K36me3 exhibited a similar CDS and 3’UTR distribution pattern to m^6^A. H3K36me3 can be directly identified and bound by METTL14. Upon meeting RNA Pol II, METTL14 then recruits other components of m^6^A MTC and mediates the deposition of m^6^A on newly synthesized RNA. Thus, the recognition of H3K36me3 by METTL14 and the specific recognition of DRACH motifs by m^6^A MTC allow for the accurate and dynamic deposition of m^6^A on the transcriptome, uncovering the importance of METTL14 in the selective and precise deposition of m^6^A.^748^ Another study found that METTL3 and METTL14 primarily bind to the promoter regions of the genome in leukemia cells and are associated with the histone H3K4me3 modification.^750^ Similarly, the m^6^A modification can also coordinately regulate histone modifications by recruiting histone-modifying enzymes and related proteins.^751^ Li et al. reported that the repressive histone mark H3K9me2 could be specifically removed by the induction of m^6^A-modified transcripts. They observed a genome-wide correlation between m^6^A and occupancy of the H3K9me2 demethylase KDM3B, and found that the m^6^A reader YTHDC1 physically interacts with and recruits KDM3B to m^6^A-associated chromatin regions, promoting H3K9me2 demethylation and gene expression.^751^ Moreover, m^6^A regulators can regulate histone modification by destabilizing histone modifying enzymes mRNAs.^747^ It is reported that METTL14 not only alters H3K27me3 modification, but also regulates H3K27ac modification by destabilizing CBP and p300 mRNAs.^747^ It is not difficult to envision that histone modifications and m^6^A RNA modifications, along with their respective writers, readers, and erasers, can engage in intricate crosstalk through mutual recognition and interaction. This complex network is anticipated to be progressively elucidated in forthcoming research.

In the past few years, it has been revealed histone modifications and m^6^A RNA modifications synergistically contribute to drug resistance in tumor, particularly in the framework of acquired resistance, through a complex crosstalk mechanism.^752^ For instance, KDM4C orchestrates ALKBH5 expression through elevating the chromatin openness, suppressing H3K9me3 and facilitating recruitment of MYB and Pol II. ALKBH5 is essential for sustaining leukemia stem cell characteristics, and mediates mRNA stability of receptor tyrosine kinase AXL by m^6^A modification (KDM4C-ALKBH5-AXL signaling axis).^753^ In glioblastoma with TMZ resistance, TMZ can induce a SOX4-dependent augmentation in chromatin openness at the region of METTL3 through enhancement of H3K27ac levels and the recruitment of RNA polymerase II. Furthermore, METTL3 decreasing perturbs the deposition of m^6^A on transcripts of genes, including EZH2, culminating in nonsense-mediated mRNA decay. This underscores the pivotal impaction for EZH2 in the modulating of METTL3 transcription through H3K27me3-independent manner.^752^

#### Histone modifications and ncRNAs

There exists a complex regulatory network among histone-modifying enzymes/histone modifications, lncRNAs, and miRNAs.^754,755^ In general, histone modifications are positioned “upstream” of lncRNAs and miRNAs, as they regulate gene expression at chromatin level, but the expression and localization of histone modifiers may also be regulated by lncRNAs and miRNAs.^756^ lncRNAs, especially those that have been well-studied for their oncogenic roles such as MALAT1, HOTAIR, H19 and so on, promote cancer development through various pathways, including interactions with histone modifications and miRNA.^757,758^ Among these three elements, miRNAs seem to be more inclined to a “downstream” position, acting as effector molecules, yet their effects can also entail changes in the levels of histone modifications and lncRNAs.^754,759^ In tumors, there is often a simultaneous dysregulation of histone modifications, lncRNAs and miRNAs, which have a synergistic effect on promoting tumor drug resistance.

Histone modifications or modifying enzymes can regulate the expression levels of lncRNAs through direct or indirect interacting, consequently affecting the levels of associated miRNAs, and ultimately leading to the induction or repression for resistance-associated pathways.^760^ For instance, as an oncogene frequently overexpressed in a variety of malignancies, *KDM4C* contributes to the progression of these cancers as well as their resistance to ionizing radiation and chemotherapy.^120^ KDM4C has been found to demethylate the *MALAT1* promoter region to boost MALAT1 expression.^761^ MALAT1 has been proved to act as a sponge of a variety of miRNAs in many cancers. Take miR-328-3p as an example, the upregulation of MALAT1 led to its downregulation in AML cells, resulting in the overexpression of Cyclin D2 (CCND2), which elevates the fraction of cells arrested in the G1 phase and diminishes the responsiveness of HL-60/A to Ara-C.^761,762^ It is essential to acknowledge that histone variants could also impact expression of lncRNA. For instance, histone H1.3 exerts a suppressive effect on the expression of H19 and inhibits the proliferative capacity of ovarian cancer cells.^763^

Moreover, lncRNAs are able to regulate histone modifications in a variety of ways, as discussed in recent reviews. In brief, lncRNAs can serve as scaffolds to bring together multiple components of chromatin-modifying complexes. For instance, the lncRNA HOTAIR interacts with the PRC2 and the LSD1/CoREST/REST complex, facilitating the coordinated regulation of histone H3K27 methylation and H3K4 demethylation.^764^ It is reported that HOTAIR mediated the switching of histone H3 lysine 27 acetylation to methylation to promote EMT in gastric cancer.^765^ lncRNAs can guide histone modification enzymes to specific genomic loci, thereby influencing the local chromatin state and gene expression. For example, lncRNA CASC9 has been demonstrated to promote esophageal squamous cell carcinoma metastasis. Mechanistically, CASC9 can interact with the transcriptional coactivator CREB-binding protein (CBP) within the nucleus. This interaction leads to an increased presence of CBP and H3K27 acetylation at the LAMC2 promoter, thereby enhancing the expression of LAMC2.^766^ The lncRNA URRCC, whose expression is upregulated in renal cell carcinoma (RCC) samples and associated with poor prognosis, leading to promote RCC cells proliferation and invasion. Mechanistically, URRCC enhances the expression of *EGFL7* via mediating histone H3 acetylation of EGFL7 promoter, activation of P-AKT signaling, and suppressing P-AKT downstream gene *FOXO3*.^767^

Compared to lncRNAs and miRNAs, other types of non-coding RNAs, such as circRNAs and piRNAs, are less frequently reported to interact with histone modifications. However, recent studies have also revealed the possibility of such crosstalk. Circ_0019435 recruited EZH2 by directly binding to suppress the expression of DKK1 and PTEN, thereby enhancing the progression of cervical cancer.^768^ In many cases, circRNA plays an anti-tumor role. Circ_SPECC1 was down-regulated in various gastric cancer cell lines. Circ_SPECC1 functions as a sponge to attach miR-526b and therefore regulates its target genes, including lysine demethylase 4A (KDM4A) and the downstream signaling target YAP1/KDM4A, and suppressing the invasion and growth of gastric cancer cells.^769^ In human bladder cancer, circXRN2, which is aberrantly downregulated in bladder cancer tissues and cell lines, suppresses tumor progression driven by H3K18 lactylation by activating the Hippo signaling pathway.^770^

## Epigenetic agents in combination with cancer therapies

**Fig. 8:**
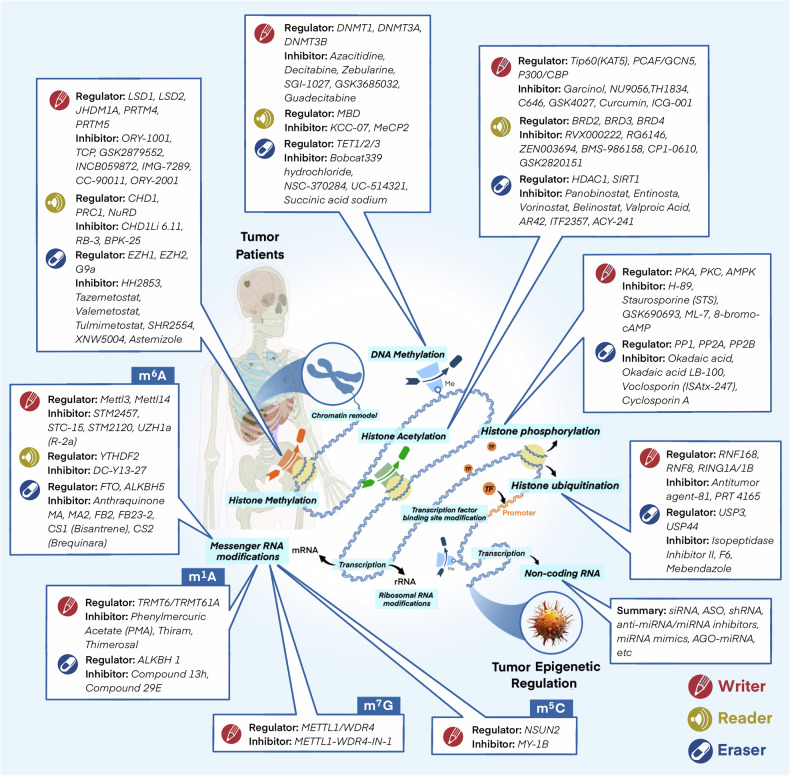
Map of various inhibitors against epigenetic regulators. It delineates the principal epigenetic pathways, succinctly outlining their functions and interconnections. Additionally, it enumerates the key epigenetic regulators and their corresponding inhibitors for each pathway mentioned. DNMT DNA methyltransferase, TET ten-eleven translocation, HDAC histone deacetylase, KDM histone demethylase, KMT histone methyltransferase, KAT histone acetyltransferase, MBD methyl-CpG binding domain

In the preceding discussion, we have systematically and comprehensively summarized the impact of epigenetics on cancer, demonstrating that epigenetic mechanisms contribute significantly to the onset and evolution of cancer. Targeting epigenetic pathways represents an innovative strategy for addressing the challenges posed by malignant tumors. Remarkably, within six-month in 2020, the FDA granted two approvals for Tazemetostat, an EZH2 inhibitor, heralding a groundbreaking new chapter in the field of epigenetic therapeutics for oncology.^771^ The utilization of epigenetic drugs in cancer therapy primarily focuses on the inhibition of aberrant DNA methylation, histone methylation, and histone acetylation.^665^ To date, several drugs targeting specific epigenetic mechanisms have been approved by FDA. These include histone deacetylase HDAC inhibitors such as vorinostat, romidepsin, and chidamide, as well as DNA methylation inhibitors like 5-azacytidine and decitabine and EZH2 inhibitor Tazemetostat above-metioned^604^ (Fig. 8). Furthermore, the combination of epigenetic drugs with other cancer treatment modalities, including radiotherapy, chemotherapy, and immunotherapy.^666^ Epigenetic drugs predominantly target regulatory mechanisms involved in the modification process to alter aberrant modification levels in cancer as described in Fig. 8. We will discuss the application of epigenetic agents in combination with other cancer therapies in clinical research subsequently.

### Targeting histone modifications

Histone modification drugs are capable of rectifying dysregulated histone modification patterns within malignant cells to suppress the aberrant gene transcriptions associated with oncogenesis. Among the pharmacological agents targeting histone modifications, histone deacetylase inhibitors (HDACi) have been the most extensively investigated and clinically utilized. Notable examples of HDACi include vorinostat, trichostatin A (TSA), panobinostat, romidepsin, and mocetinostat.^772^ HDACi can directly induce alteration of the cancer epigenome leading to reactivation of epigenetically silenced genes and consequently cancer cell growth arrest, promotion of differentiation and induction of apoptosis.^28^ At present, HDACi are employed as a therapeutic option for hematological cancers, such as cutaneous T-cell lymphoma, peripheral T-cell lymphoma, multiple myeloma, and non-Hodgkin’s T-cell lymphoma.^773,774^Furthermore, HDACi have demonstrated potential efficacy in the treatment of various solid tumors, including breast cancer, lung cancer, and glioma.^775,776^ The preceding part describes a comprehensive picture on the epigenetic agents for overcoming resistance to cancer treatments (Fig. 8). At present, the combination of HDACi with radiotherapy, chemotherapy, immunity, and targeted therapy has also made progress in clinical research (Table 2). In conjunction with cisplatin, panobinostat is utilized alongside tamoxifen to address chemoresistance in breast cancer. Furthermore, the combination of Vorinostat with 5-FU, leucovorin, and oxaliplatin has demonstrated efficacy in overcoming chemoresistance in CRC. Additionally, HDACi serve as sensitizers for both conventional fractionated radiotherapy and fractionated stereotactic radiation therapy (NCT01378481 and NCT00821951). In the field of immunotherapy, panobinostat in conjunction with the CTLA4 inhibitor ipilimumab have been incorporated into clinical research studies. Besides, vorinostat combined with the anti-PD-1 monoclonal antibody in breast cancer has achieved phase II clinical trial (NCT00258349). Pembrolizumab, have been incorporated into clinical research studies. Furthermore, the combination of HDACi with the EGFR inhibitor lapatinib and the low-dose proteasome inhibitor bortezomib has demonstrated a potential effect in overcoming resistance to targeted therapies.

**Table 2:** Epigenetic drugs combination with cancer therapy

	Regulator	Epigenetic targeting agents	Chemotherapy	Conditions	Trial lD
Histone acetylation	HDAC	Panobinostat	Tamoxifen	Breast cancer	NCT00993642
			Temozolomide	Melanoma	NCT00925132
			Melphalan	Myeloma	NCT00743288
			Bortezomib	Lymphoma	NCT00901147
			Carboplatin	Hodgkin lymphoma	NCT01169636
			Epirubicin	Ovarian cancer	NCT00878904
		AR-42	Decitabine	Acute myeloid leukemia	NCT01798901
			Pomalidomide	Relapsed multiple myeloma	NCT02569320
		ITF2357		Hodgkin’s lymphoma	NCT00496431
		Valproic Acid	Fludarabine	Chronic lymphocytic leukemia	NCT00524667
		Belinostat	Platinum	Tumors of the thymus	NCT00589290
		Entinostat	Azacitidine	Colorectal cancer	NCT01105377
			Capecitabine	High Risk Breast Cancer	NCT03473639
		Vorinostat	Ixabepilone	Breast cancer	NCT01084057
			Tamoxifen	Breast cancer	NCT01194427
			5-fluorouracil, Leucovorin and Oxaliplatin	Colorectal cancer	NCT00336141
		Romidepsin	Gemcitabine	Solid tumors	NCT00379639
Histone methylation	LSD1	SP-2577	Topotecan	Ewing sarcoma	NCT03600649
		Seclidemstat	Azacitidine	Chronic myelomonocytic leukemia	NCT04734990
		CC-90011	Cisplatin and Etoposide	Small cell lung cancer	NCT03850067
		Iadademstat	Azacitidine and Venetoclax	Acute myeloid leukemia	NCT06357182
DNA methylation	DNMT	Azacitidine	CAPOX	Colorectal cancer	NCT01193517
			Cyclophosphamide	Peripheral T-cell lymphoma	NCT05678933
			Abraxane	Breast cancer	NCT00748553
		Decitabine	Cyclophosphamide	Acute myeloid leukemia	NCT06383572
			Platinum	Ovarian cancer	NCT00477386
			Fludarabine phosphate	Myeloid malignancies	NCT06383572
			Carboplatin-Paclitaxel	Ovarian cancer	NCT02159820
		Guadecitabine	Carboplatin	Small cell lung cancer, Ovarian cancer	NCT03913455 NCT01696032
			Cladribine	Acute myeloid leukemia	NCT02096055
			Irinotecan	Colorectal cancer	NCT01896856
Histone acetylation	HDAC	Vorinostat	Pembrolizumab Tamoxifen	Breast cancer	NCT04190056
				Breast neoplasms	NCT02395627
			Trastuzumab	Breast cancer	NCT00258349
			Carboplatin Paclitaxel Bevacizumab	Non-small cell lung cancer	NCT00702572
			Pomalidomide	Relapse/refractory multiple myeloma	NCT01979276
		Belinostat	Tremelimumab Durvalumab	Urothelial Carcinoma	NCT05154994
		Panobinostat	Ipilimumab	Melanoma	NCT02032810
			Paclitaxel, Carboplatin Bevacizumab	Solid Tumors	NCT00556088
		Entinostat	Pembrolizumab	Solid Tumors	NCT02909452
			Nivolumab	Cholangiocarcinoma and Pancreatic Adenocarcinoma	NCT03250273
Histone methylation	EZH2	Tulmimetostat	Pembrolizumab	Non-small cell lung cancer	NCT05467748
		XNW5004	Pembrolizumab	Nasopharyngeal carcinoma	NCT06022757
		CPI-1205	Ipilimumab	Melanoma and Non-small cell lung cancer	NCT03525795
		SHR2554	SHR1701	B-cell Lymphomas	NCT04407741
	LSD1	ORY-1001	Atezolizumab or Durvalumab	Small cell lung cancer	NCT06287775
		IMG-7289	Atezolizumab	Small cell lung cancer	NCT05191797
		SP-2577	Pembrolizumab	Small cell ovarian cancer	NCT04611139
		CC-90011	Nivolumab	Small cell lung cancer or Non-small cell lung cancer	NCT04350463
		Bomedemstat	Atezolizumab	Small cell lung cancer	NCT05191797
DNA methylation	DNMT	Azacytidine	Pembrolizumab	Carcinoma of colon and rectum	NCT02260440 NCT02512172
				Acute myeloid leukemia	NCT02845297 NCT04284787 NCT03769532
				Non-small cell lung cancer	NCT02546986
				Hodgkin lymphoma	NCT05355051
				Ovarian cancer	NCT02900560
				Pancreatic ductal adenocarcinoma	NCT03264404
				Melanoma	NCT02816021
				Fallopian tube cancer	NCT02901899
			Nivolumab	Acute myeloid leukemia	NCT02397720 NCT03825367 NCT04913922
				Non-small cell lung cancer	NCT01928576
				Head and neck squamous-cell carcinoma	NCT05317000
				Hodgkin lymphoma	NCT05162976
				Osteosarcoma	NCT03628209
			Durvalumab	Acute myeloid leukemia	NCT02775903
				Ovarian cancer, Breast cancer	NCT02811497
				Pancreatic cancer	NCT04257448
				Non-small cell lung cancer	NCT02250326
				Peripheral T-cell lymphoma	NCT03161223
			Camrelizumab	Acute myeloid leukemia	NCT05772273
				Peripheral T-Cell Lymphoma	NCT05559008
			Avelumab	Acute myeloid leukemia	NCT03390296
			Spartalizumab	Acute myeloid leukemia	NCT03066648
			Ipilimumab	Acute myeloid leukemia	NCT02397720
			PF-04518600	Acute myeloid leukemia	NCT03390296
		Decitabine	Pembrolizumab	Acute myeloid leukemia	NCT02996474 NCT03969446
				Peripheral Triple-negative Breast Cance	NCT05673200
				HER2-negative Breast Cancer	NCT02957968
				Non-small cell lung cancer	NCT03233724
				CNS solid tumors and Lymphomas	NCT03445858
				T-cell lymphoma	NCT03240211
			Nivolumab	Mucosal melanoma	NCT05089370
				Acute myeloid leukemia	NCT04277442
				Non-small cell lung cancer	NCT02664181
				Myelodysplastic syndromes, Acute myeloid leukemia	NCT02664181
			Durvalumab	Head and neck cancer	NCT03019003
			Camrelizumab	Hodgkin lymphoma	NCT0451061, NCT0325096, NCT0451408, NCT04233294
				Primary mediastinal large B-cell lymphoma	NCT03346642
				Acute myeloid leukemia	NCT04353479
			Spartalizumab	Acute myeloid leukemia	NCT03066648
			Ipilimumab	Acute myeloid leukemia	NCT02890329
		Guadecitabine	Pembrolizumab	Lung cancer	NCT03220477
				Non-small cell lung cancer/Castration-resistant prostatic cancer	NCT02998567
				Fallopian tube cancer	NCT02901899
			Nivolumab	Melanoma, Non-small cell lung cancer	NCT04250246
			Durvalumab	Kidney cancer	NCT03308396
				Small cell lung cancer	NCT03085849
				Hepatocellular carcinoma, Gallbladder cancer, Pancreatic cancer, Intrahepatic cholangiocarcinoma	NCT03257761
			Atezolizumab	Chronic myelomonocytic leukemia and Acute myeloid leukemia	NCT02935361
				Acute myeloid leukemia	NCT02892318
				Ovarian, Fallopian tube, or Primary peritoneal cancer	NCT03206047
				Urothelial carcinoma	NCT03179943
			Tremelimumab	Small cell lung cancer	NCT03085849
			Ipilimumab	Melanoma, Non-small cell lung cancer	NCT04250246
				Melanoma	NCT02608437
	TET1/2/3	Auranofin	Sirolimus	Ovarian cancer	NCT03456700
				Solid tumors or Non-Small cell lung cancer	NCT02126527
				Non-Small cell lung cancer or Small cell lung cancer	NCT01737502
Histone acetylation	HDAC	Entinostat	Exemestane	Breast Cancer	NCT02820961
		Vorinostat	Olaparib	Breast cancer, Metastatic breast cancer	NCT03742245
			Anastrozole Letrozole Exemestane	Breast cancer	NCT01720602
			lapatinib	Breast cancer, Neoplasm, Metastasis	NCT01118975
			Bortezomib	Advanced multiple myeloma	NCT00111813
				Multiple myeloma	NCT00310024
				Metastatic or Unresectable solid tumors	NCT00227513
				Glioblastoma multiforme	NCT00641706
		Romidepsin	Flavopiridol	Solid tumors	NCT00094978
			Decitabine	Pulmonary and Pleural malignancies	NCT00037817
				Leukemia, Myeloproliferative disorders, or Myelodysplastic syndromes	NCT00114257
			Alisertib	Aggressive B-cell lymphoma and T-cell lymphoma	NCT01897012
			Bortezomib	Relapse/refractory multiple myeloma	NCT00431990
				Chronic lymphocytic leukemia/Small lymphocytic lymphoma, Indolent B-cell lymphoma, Peripheral T-cell lymphomas, and Cutaneous T-cell lymphoma	NCT00963274
			Pralatrexate	Lymphoid malignancies	NCT01947140
			CC-486	Lymphoid malignancies	NCT01998035
			Erlotinib	Solid tumors	NCT01302808
			Carfilzomib	Cutaneous T-cell lymphoma	NCT01738594
				Refractory B-cell lymphoma and T-cell lymphoma	NCT02341014
		Panobinostat	Bicalutamide	Prostate Cancer	NCT00878436
			Pemetrexed	Non-Small Cell Lung Cancer	NCT00907179
Histone methylation	EZH2	Tazemetostat	Enzalutamide	Prostate cancer	NCT04179864
		PF-06821497	Enzalutamide	Prostate cancer	NCT06629779
		Tazemetostat	Dabrafenib	Melanoma	NCT04557956
	LSD1	IMG-7289	Venetoclax	Acute Myeloid Leukemia	NCT05597306
		CC-90011	Venetoclax and Azacitidine	Acute Myeloid Leukemia	NCT04748848
DNA methylation	DNMTi	Azacitidine	Ivosidenib and Venetoclax	Hematologic malignancies	NCT03471260
			Chidamide	T-cell lymphoma	NCT04480125
			Entinostat	Non-small cell lung cancer	NCT00387465
			SAR443579	Hematological malignancies.	NCT06508489
			Epacadosta	Non-small cell lung cancer and colorectal cancer	NCT02959437
			Venetoclax	Acute myeloid leukemia	NCT05471700
		Decitabine	Avapritinib	Hematologic neoplasm	NCT06327685
			Panitumumab	Colorectal Cancer	NCT00879385
			Tagraxofusp	Chronic myelomonocytic leukemia	NCT05038592
			Enzalutamide	Prostate cancer	NCT05037500
			Venetoclax	Acute myeloid leukemia	NCT05455294
			MBG453	Melanoma/Non-small cell lung cancer	NCT02608268
			Selinexor	Ovarian cancer	NCT05983276
			Ruxolitinib	Acute myeloid leukemia	NCT02076191
		Guadecitabine	Pembrolizumab	Lung cancer	NCT03220477
				Non-small cell lung cancer Prostatic cancer	NCT02998567
				Ovarian, Primary peritoneal or Fallopian tube cancer	NCT02901899
			Nivolumab	Melanoma, Non-small cell lung cancer	NCT04250246
			Durvalumab	Advanced kidney cancer	NCT03308396
				Small cell lung cancer	NCT03085849
				Hepatocellular carcinoma, Gallbladder cancer, Pancreatic cancer, Intrahepatic cholangiocarcinoma	NCT03257761
			Atezolizumab	Chronic myelomonocytic leukemia, Myelodysplastic syndromes and Acute myeloid leukemia	NCT02935361
				Acute myeloid leukemia	NCT02892318
				Ovarian, Fallopian tube, or Primary peritoneal cancer	NCT03206047
				Urothelial carcinoma	NCT03179943
			Tremelimumab	Small cell lung cancer	NCT03085849
			Ipilimumab	Melanoma	NCT02608437
				Melanoma/Non-small cell lung cancer	NCT04250246
Histone acetylation	HDAC	Vorinostat	Standard fractionation of 3.0 Gy per day, a total dose of 30 Gy	Non-Small cell lung cancer	NCT00821951
			Dose of 50.4 Gy in 1.8 Gy fractions	Pancreatic cancer	NCT00831493
			Fractionated stereotactic radiation therapy	Glioma	NCT01378481
			1.8-Gy fractions to a total dose of 50.4 Gy	Pancreatic cancer	NCT02349867
		Panobinostat	10 fractions of stereotactic radiation therapy, over 2 weeks	Brain tumors	NCT01324635
		Vorinostat	A dose of 70 Gy	Non-Small cell lung cancer	NCT01059552
		Valproate	2 Gy fraction from Monday	Cervical cancer	NCT00404326
		Valproic Acid	2 Gy fractions to 60 Gy	Brain tumors	NCT00302159
Histone methylation	EZH2	Temozolomide	2 Gy fractions to 60 Gy	Glioblastoma	NCT04396860 NCT03514069 NCT05739942
DNA methylation	DNMT	Decitabine	2 Gy fraction 5 times per week	Lymphomas	NCT03445858

In addition to HDACi, EZH2 inhibitors are extensively utilized clinically. EZH2 inhibitors are categorized as disrupting the structural integrity of the Polycomb Repressive Complex 2 (PRC2) and targeting EZH2 methyltransferase activity, such as tazemetostat and valemetostat, function by directly reducing levels of trimethylated histone H3K27me3 through the inhibition methyltransferase activity of EZH2.^777,778^ Moreover, GSK126 the other kind of EZH2 inhibitor, which disrupts protein-protein interactions among PRC2 subunits, has achieved phase I clinical trials for the treatment of various cancers (NCT02082977). In overcoming targeted therapy, EZH2 inhibitors have also made breakthroughs. Tazemetostat, effectively inhibit prostate cancer progression when combined with enzalutamide, an androgen receptor inhibitor (NCT04179864). Part of EZH2 inhibitors is in clinical trials to verify the feasibility of combining with immune checkpoint inhibitors, such as tulmimetostat, XNW5004, CPI-1205 and SHR2554 (Table 2). Moreover, EZH2 inhibitors has been proven to be a booster radiotherapy (Table 2).

Over the past decade, substantial progress has been achieved in the domain of targeted drug research concerning histone readers. Notably, INCB054329, a BET domain inhibitor, is undergoing clinical trials for the treatment of Ewing’s sarcoma and advanced cancers (NCT03514407 and NCT02431260). Furthermore, the pharmaceutical research of BRD and BET domain protein inhibitors, specifically BMS-986158 and BMS-986378, have progressed to phase I trials in the context of pediatric cancer (NCT03936465). In general, the feasibility of targeting histone modifications in cancer therapy has been demonstrated, both as a standalone treatment and in combination with other therapeutic approaches.

### Targeting DNA methylation

DNA methylation inhibitors have garnered considerable attention in oncological research owing to their potential to revise abnormal DNA methylation patterns linked to tumorigenesis.^41,779,780^ These pharmacological agents predominantly target DNMTs, which are categorized into cytosine analog oligonucleotide drugs, SAM competitors, and DNA binders. Notably, cytosine analogs have been utilized in clinical settings. These analogs are integrated into DNA during the process of DNA synthesis. During the catalytic activity of DNMTs, these cytosine analogs can form covalent bonds with DNMTs, thereby inhibiting DNMTs dissociating from chromatin,^41^ 5-azacytidine (azacitidine) and 5-aza-2’-deoxycytidine (decitabine) have received clinical approval from the FDA for the treatment of myelodysplastic syndromes and acute leukemia. Furthermore, additional DNMT inhibitors are presented in the Table 2. DNMTs inhibitors play a role in reactivating excessively suppressed tumor suppressor genes, including p53 and p21, in cancer, thereby playing a crucial role in overcoming cancer treatment resistance (Fig. 8).^515,781,782^ Second-generation DNMT inhibitors with better pharmacokinetic properties, such as guadecitabine, have been developed. While DNMT inhibitors are established as a standard treatment for hematological malignancies, their efficacy in solid tumors remains limited. Consequently, current research is increasingly focused on their application as adjuvant therapies to enhance treatment outcomes.^41,780,783^ Notably, the integration of DNMT inhibitors with other therapeutic modalities, including radiotherapy, targeted therapy, chemotherapy, and immunotherapy, has demonstrated potential in enhancing the overall efficacy of cancer treatment in clinical trials (Table 2). For example, azacitidine, decitabine and guadecitabine combined with a variety of chemotherapy drugs, such as platinum, carboplatin and abraxane, have a significant effect on overcoming chemotherapy resistance in small cell lung cancer, acute leukemia and CRC (Table 2). Furthermore, the integration of DNMT inhibitors with widely used immune checkpoint inhibitors has progressed to clinical trial phases. Specifically, the combination of DNMT inhibitors with pembrolizumab, nivolumab, camrelizumab, and durvalumab is being investigated across a range of solid tumors (Table 2). The efficacy of DNMT inhibitors as adjuvant agents in cancer therapy has been substantiated through both preclinical and clinical studies.

### Targeting ncRNAs

In the preceding section, we provided an overview of the aberrant expression of ncRNAs in cancer, delving into the mechanisms by which resistance to various cancer treatments can be overcome, which indicates that ncRNAs may play a crucial role in cancer therapy and in surmounting resistance to other therapeutic modalities. Presently, two principal strategies are employed to target ncRNAs for cancer treatment: the inhibition of overexpressed ncRNAs and the restoration of suppressed ncRNAs within cancerous cells.^55^ Therefore, it is necessary to discovery targeted inhibitors of ncRNAs and delivery systems to inhibit ncRNAs. There are six primary types of ncRNA inhibitors: (1) Antisense oligonucleotides (ASOs) bind to complementary RNA sequences, blocking their function and promoting degradation via RNase-H cleavage; (2) Antisense miRNAs (antagomirs) bind to complementary miRNAs, leading to their degradation and preventing them from interacting with target mRNAs. (3) Artificial miRNA sponge: synthetic RNA with multiple miRNA binding sites sequesters miRNA from its target mRNA; (4) Small molecules: disrupt any stage of RNA transcription; (5) siRNAs and shRNAs: synthetic double-stranded RNAs bind to complementary non-coding RNAs in AGO2, causing target RNA degradation; (6) CRISPR/Cas9 editing: uses Cas9 nuclease and guide RNA to accurately cut target nc RNA.^784^ Common delivery methods for ncRNA therapeutics include lipid nanoparticles, exosomes, antibodies, and peptides.^785–787^The FDA has approved this approach for certain diseases. For instance, vitravene (fomivirsen sodium), the first antisense nucleotide drug, is FDA-approved for cytomegalovirus retinitis. Nearly 20 RNA drugs are on the market, but none target tumors.^788^

ncRNA drugs face challenges in achieving clinical efficacy. In clinical trials for breast cancer and NSCLC, ASO drugs have not demonstrated significant anticancer efficacy.^789,790^ Currently, ncRNAs serve primarily as molecular markers in cancer treatment. Particularly, the FDA has approved the lncRNA PCA3 as a diagnostic marker for prostate cancer.^791^ Numerous ncRNAs are under investigation as cancer markers in clinical trials. In breast cancer chemotherapy, circulating miRNA types and levels in patients’ blood are monitored to aid in prognostication and treatment planning on an individual patient basis (NCT01722851).

The other approach for targeting ncRNAs in cancer that overexpression of ncRNAs, particularly using miRNA mimics to replace down-regulated tumor suppressor miRNAs, faces challenges such as off-target side effects. For instance, the miR-34 mimic MRX34 caused severe adverse events, including cytokine release syndrome, in five patients, halting a phase I cancer trial.^792^ Furthermore, off-target effects in non-neoplastic tissues pose a challenge to RNA drug therapy. The XIAP-targeting antisense oligonucleotide AEG35156, when taken up by glial cells or neurons, leads to a reduction in XIAP levels within these cells, which can result in peripheral chemotherapy-induced neuropathy in patients.^793^

Currently, ncRNAs emerge as viable predictors for cancer therapy in clinical trials, but integrating them with existing treatments poses challenges like tolerance, toxicity, and off-target effects. Preclinical studies show a strong link between ncRNAs and treatment resistance. Advances in single-cell sequencing and understanding tumor heterogeneity are expected to enhance ncRNA’s role in cancer treatment.

### Targeting RNA modifications

Currently, there are no RNA modification inhibitors approved by the FDA for clinical application. Significant advancements have been achieved in the development of RNA modification therapeutics in recent years. Notably, FTO inhibitors are highly effective against cancer.^794,795^ Moreover, since the elucidation of the crystal structure of FTO in 2010, a range of inhibitors targeting its substrate binding sites has been developed, including anthraquinone, entacapone, meclofenamic acid (MA), MA2, as well as FB23 and FB23-2.^795,796^ The selectivity for FTO and cell membrane permeability improved with each generation.^797,798^ In preclinical investigations, FTO has demonstrated efficacy when used in conjunction with cancer therapeutic agents. MA enhances the sensitivity of leukemia cells to TKIs. Furthermore, entacapone combined with imatinib, a kind of TKIs, for leukemia treatment completed phase I clinical trials in 2022, highlighting its potential as a therapeutic enhancer in clinical settings (NCT04006769). In addition to FTO, many inhibitors of METTL3 have emerged in recent years, such as STM2457 STC-15, UZH1a (R-2a) STM2120. STC-15 for the treatment of AML has entered the clinical phase I trial stage in 2022 (NCT05584111). In thyroid cancer, the expression of METTL3 is relevant to anti-PD-1 therapy resistance.^631^ There is a paucity of studies on METTL3 inhibitor combined with other cancer treatments. METTL3 inhibitors may have the potential to enhance the efficacy of cancer therapies. This will be a new research direction for cancer treatment. It may be a novel avenue of oncology therapeutics. Furthermore, m6A reader inhibitors were demonstrated to function as radiosensitizers in 2023. The m6A reader inhibitor, DC-Y13-27, was found to directly bind to the YTHDF2 protein, thereby inhibiting its interaction with m6A-RNA in vitro, which alters the differentiation of myeloid-derived suppressor cells (MDSCs) and reduces their infiltration and suppressive activity. Consequently, DC-Y13-27 not only enhances the efficacy of radiotherapy but also augments the anti-tumor effects when radiation is combined with PD-L1 antibody therapy.

In conclusion, the challenge of achieving sustained cancer therapeutic doses with single- epigenetic agent arises from their low selectivity and broad spectrum of gene regulation. Consequently, optimizing pharmacokinetics and minimizing off-target toxicity are essential to establish a durable and tolerable therapeutic response in patients. The concept of utilizing low-dose epigenetic inhibitors to enhance therapeutic outcomes represents an innovative approach in the clinical application of these inhibitors. Over the past few years, a multitude of clinical trials have been undertaken to assess the specific approach. The integration of this therapeutic concept with the rapidly evolving techniques of single-cell sequencing is potential to become a pivotal strategy in overcoming resistance to cancer treatments.

## Perspectives and challenges

In recent years, the domain of epigenetics has undergone substantial expansion, incorporating a diverse array of mechanisms including nucleosome positioning, chromatin remodeling, histone modifications, DNA methylation, RNA modifications, alternative RNA splicing, and non-coding RNAs (such as lncRNAs, miRNAs, circRNAs, piRNAs, among others), as well as pseudogenes (non-coding DNA).^799^ These elements can drive heritable alterations in gene function, culminating in phenotypic variations without modifications to the DNA sequence. It is crucial to acknowledge that the expression and activity of modifiers and regulators responsible for epigenetic modifications remain substantially affected by mutations within genetic code. This underscores the intricate interplay between genetic and epigenetic information.^800^ A comprehensive understanding of cellular life processes can only be attained by elucidating the mechanisms underlying the organization, transmission, and expression of both genetic and epigenetic information.

A considerable body of evidence indicates that widespread epigenetic dysregulation exists in tumors and is associated with resistance to cancer therapy. The intricate interplay between various epigenetic modifications is increasingly recognized as a key driver of therapeutic resistance in cancer. This epigenetic dysregulation not only influences the behavior of cancer cells but also reshapes the tumor microenvironment, thereby profoundly impacting the efficacy of cancer treatments. The complex network of epigenetic interactions can sculpt a landscape of resistance, enabling the tumor cells to adapt and survive under treatment. Understanding and targeting these epigenetic crosstalk pathways may offer novel therapeutic strategies to overcome resistance and enhance the responsiveness of tumors to treatment. However, a considerable challenge arises from the complexity and heterogeneity of epigenetic alterations within tumors. It is now recognized that within a single type of cancer, there can be a multitude of epigenetic disruptions, and due to the heterogeneity of cellular populations within the tumor, pinpointing the core drivers is exceedingly difficult. The interplay of various epigenetic modifications, such as DNA methylation, histone modifications, and non-coding RNAs, contributes to the phenotypic and functional diversity of cancer cells, ultimately fostering therapeutic resistance. Additionally, different therapeutic approaches can elicit distinct epigenetic landscape changes within tumors. For instance, there is substantial evidence that radiation therapy can modify histone modifications and chromatin structure within tumors, and it may also affect the levels of non-coding RNAs.^801^ However, it is rarely reported whether the original network of epigenetic crosstalk undergoes significant changes throughout the treatment process, whether the core of the original epigenetic crosstalk network is altered, and what impact such changes may have on therapy. This epigenetic heterogeneity, both between intertumor and intratumor, is a hallmark of human cancers and is increasingly recognized as a critical driver of resistance to cancer therapies.

The advancement of omics technologies such as multiplexed-scAEBS, scBS-Seq and single-cell ATAC-Seq have ushered in new opportunities for characterizing the epigenetic landscape of tumors and identifying resistance-associated factors.^802^ These technologies enable a more comprehensive view of the epigenome, which includes not only DNA methylation and histone modifications but also the complex interactions between different epigenetic regulators. The capacity to profile multiple epigenetic marks across a diverse array of tumors is crucial for unraveling the heterogeneity and pinpointing potential therapeutic targets. As research advances, there is an expanding trend towards integrating these omics data to uncover the complex network of epigenetic interactions that contribute to cancer progression and drug resistance. This approach offers promise for the development of innovative therapeutic strategies that target the epigenome, potentially overcoming resistance and improving treatment outcomes in cancer patients.

Currently, epigenetic medications often exhibit diminished efficacy in solid tumors, primarily due to the inherent heterogeneity and complex crosstalk within the epigenetic landscape.^604,666,799^ Consequently, they are not typically considered first-line treatments for most cancers. However, accumulating evidence suggests that epigenetic drugs can significantly contribute to the management of solid tumors by alleviating resistance to various cancer therapies. Despite their utility, these two therapeutic approaches encounter significant resistance when applied to solid tumors. Furthermore, technological progress has updated the traditional treatment methods. For instance, FLASH radiotherapy, an avant-garde technique of radiotherapy with a dose rate exceeding 40 Gy/s, exhibits a significant protective effect on mitochondria, outperforming traditional radiotherapy methods. FLASH therapy may exert a regulatory influence on histone modifications, notably histone lactylation, because of the protection of mitochondria with FLASH radiotherapy.^803^ Moreover, FLASH irradiation does not markedly compromise tumor vasculature and maintains the integrity of drug delivery systems. This preservation of vascular function, in turn, enhances the delivery of epigenetic therapeutics to solid tumors, potentially alleviating the radiotherapy resistance.^804^Nevertheless, the involvement of epigenetic mechanisms in drug resistance remains inadequately understood, and no established combination strategies have been developed for clinical implementation. Consequently, further investigation into epigenetic resistance within the contexts of endocrine therapy and differentiation therapy represents a prospective avenue for the development of epigenetic drugs in cancer treatment.

More interestingly, recent research has identified a noteworthy phenomenon wherein skin cells retain a long-term memory of radiotherapy via epigenetic inheritance, subsequently hindering wound healing.^805^ This raises questions about whether epigenetic inheritance similarly contributes to the retention of radiotherapy memory in tumor tissues. Furthermore, it prompts an inquiry into whether other therapeutic modalities induce long- lasting memory in tumors through epigenetic mechanisms. The implications of such long-term therapeutic memory for cancer treatment, whether advantageous or detrimental, warrant further investigation.

The advancement of epigenetics-based drugs in oncology can be advanced through two primary avenues. The first involves employing single-cell epigenetics technology to identify critical regulatory elements. The second entails integrating these insights with other therapeutic modalities to address and potentially surmount challenges related to drug resistance. In summary, the exploit of epigenetic insights contributes to epigenetics-based strategies, which are expected to display benefits in cancer therapy.

In conclusion, we have provided a comprehensive overview of the widespread epigenetic dysregulation in cancer and its association with therapeutic resistance in this review. We have focused on the molecular mechanisms by which epigenetic networks drive therapeutic resistance across different therapeutic contexts, with particular emphasis on the pivotal role of epigenetic crosstalk. Importantly, targeting epigenetic regulators/modifiers represents a promising strategy to overcome therapeutic resistance. We hope that this review will serve as a foundational and comprehensive reference for future research.
